# Lipid Nanoparticle Surface Engineering with Heparosan Polysaccharides Enhances mRNA Efficacy via Reduced Endosomal Retention

**DOI:** 10.1021/acsnano.6c11865

**Published:** 2026-09-24

**Authors:** Yuxin He, Lin Wang, Abigael P. Williams, Trisha I. Valerio, Yuanhong Sun, James Bowman, Samantha D. Ricketts, Savannah Hammond, Aidan Sison, Jezan Alexandre, Zachary Niastor, Wei R. Chen, Dixy E. Green, Kar-Ming Fung, Paul L. DeAngelis, Stefan Wilhelm

**Affiliations:** † Stephenson School of Biomedical Engineering, 6187 University of Oklahoma, Norman, Oklahoma 73019, United States; ‡ Department of Pathology, University of Oklahoma Health Campus, Oklahoma City, Oklahoma 73104, United States; § Department of Biochemistry and Physiology, University of Oklahoma Health Campus, Oklahoma City, Oklahoma 73104, United States; ∥ Institute for Biomedical Engineering, Science, and Technology (IBEST), University of Oklahoma, Norman, Oklahoma 73019, United States; ⊥ Stephenson Cancer Center, University of Oklahoma, Oklahoma City, Oklahoma 73104, United States; # Harold Hamm Diabetes Center, University of Oklahoma, Oklahoma City, Oklahoma 73104, United States; ∇ Materials Science and Engineering Program, University of Oklahoma, Norman, Oklahoma 73019, United States

**Keywords:** Lipid nanoparticles, mRNA delivery, endosomal escape, PEG-free, nanomedicine, nanoparticle surface engineering, heparosan polysaccharides

## Abstract

In this study, we developed a library of mRNA lipid nanoparticles (LNPs) containing clinically used ionizable lipids. We surface-modified these nanoparticles with heparosan polysaccharides and systematically compared their performance with conventional poly­(ethylene glycol) (PEG)-modified formulations. Our heparosan (HEP)-coated LNPs increased mRNA expression in dendritic and macrophage cell lines by up to ∼10-fold. Mechanistic studies using live-cell imaging and intracellular nanoparticle trafficking analysis revealed that HEP-modified LNPs exhibited reduced colocalization with late endosomal (Rab7^+^) and lysosomal (LAMP1^+^) compartments. These findings are consistent with enhanced endosomal escape of HEP-modified formulations. Repeated subcutaneous administrations of HEP-modified firefly luciferase mRNA-LNPs demonstrated sustained expression and distinct response dynamics in mice. We further observed significantly enhanced humoral anti-spike protein immune responses in mice after subcutaneous administration of HEP-modified LNPs containing SARS-CoV-2 spike S-2P mRNA. Histopathological evaluation and spleen immune cell profiling showed no observable toxicity or adverse effects. Overall, these results demonstrate the effects of HEP surface modification on mRNA LNP expression, intracellular trafficking, and *in vivo* activity, and support HEP-based surface engineering as a PEG-free approach for nucleic acid delivery and potential future applications in nanomedicine.

## Graphical abstract

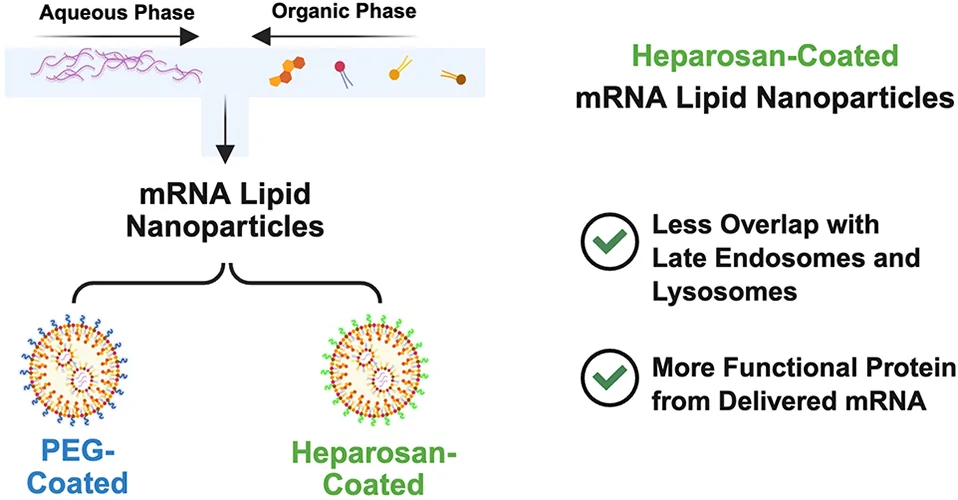

## Introduction

LNPs have been approved by the FDA and other regulatory agencies as safe and effective nanomedicine-based delivery systems for messenger RNA (mRNA) and other nucleic acid payloads.^1^ Despite this translational achievement and clinical success, the performance of LNPs is highly dependent on the nanoparticles’ lipid composition and surface chemistry, which govern colloidal stability, biodistribution, cellular interactions and uptake, and intracellular trafficking.^2^ Most clinically used liposomal and LNP-based nanomedicines use lipid components chemically modified with PEG. The PEG-lipid components are used to PEGylate the nanoparticles by displaying them on their surfaces. PEGylation of nanoparticles is typically used to improve colloidal stability and reduce nonspecific interactions, such as with proteins and other biomolecules.^3^

However, PEGylation of nanomedicines has limitations, including reduced interactions with cells and reduced cellular uptake of nanoparticles, potential PEG immunogenicity, and accelerated blood clearance (ABC) upon repeated dosing. These challenges highlight the need for alternative surface engineering strategies that maintain nanoparticle stability while enhancing the safety, effectiveness, and efficiency of these nanomedicines.^4–8^

To overcome these constraints, we previously established a post-formulation surface engineering strategy utilizing heparosan, a naturally occurring, non-immunogenic polysaccharide derived from the human glycosaminoglycan biosynthesis pathway.^9–16^ By incorporating a functional thiol-lipid such as 1,2-dipalmitoyl-*sn*-glycero-3-phosphothioethanol (16:0 PTE) into the LNP shell, orthopyridyl disulfide-functionalized HEP (OPSS-HEP) can be covalently conjugated via disulfide exchange.^9–16^ This modular approach preserves the core formulation process while enabling precise, surface-specific functionalization. Initial evaluations confirmed that HEP-modified LNPs possess excellent colloidal stability, minimal toxicity, and in vivo transfection profiles comparable to traditional PEGylated control formulations.^14^

Despite the clear advantages of HEP surface modification, two critical gaps remain: the compatibility of this strategy across diverse, clinically approved ionizable lipid classes and polymer chain lengths has not been systematically established, and the precise intracellular mechanism by which HEP enhances LNP performance remains unresolved. Efficient cellular internalization alone does not guarantee robust therapeutic efficacy; standard LNPs routinely undergo extensive endolysosomal entrapment and subsequent degradation, thereby limiting cytosolic release to a minor fraction of the total dose.^17–22^ Building upon our prior surface-engineering platform, we hypothesized that HEP functionalization fundamentally alters the intracellular trafficking pathways of mRNA-LNPs, thereby mitigating endolysosomal retention and promoting productive cytosolic entry and enhanced translation.

To test this hypothesis, we systematically evaluated HEP surface engineering across multiple clinically relevant ionizable lipids and varying HEP molecular weights, thereby establishing an optimized PEG-free LNP architecture that was directly benchmarked against 1,2-dimyristoyl-*rac*-glycero-3-methoxypolyethylene glycol-2,000 (DMG-PEG2000) control formulations. Combining live-cell confocal microscopy, quantitative colocalization analyses, and high-throughput flow cytometry with functional *in vitro* assays and *in vivo* bioluminescence tracking, we demonstrate that HEP-modified LNPs exhibit significantly reduced endosomal entrapment, yielding superior mRNA delivery efficiency and cytosolic bioactivity in antigen-presenting cells (APCs) while maintaining favorable safety profiles. By directly connecting polysaccharide surface chemistry to intracellular trafficking fate, this study elucidates the mechanistic basis of HEP-mediated nucleic acid delivery and provides rational design criteria for next-generation, non-PEGylated nanomedicines.

## Results and Discussion

### Establishing Heparosan Polysaccharide Surface Engineering for Clinically Relevant mRNA Lipid Nanoparticle Formulations

The successful synthesis and functionalization of OPSS-HEP were confirmed by chemical characterization and PAGE analysis (Figure S1). The chemical structures of OPSS-HEP and the corresponding OPSS-PEG conjugate are shown in Figure S1A,B. Alcian Blue-stained PAGE revealed well-defined bands for both HEP-NH_2_ and OPSS-HEP, indicating that conjugation with OPSS preserved the integrity of the heparosan backbone (Figure S1C). Functionalization of the polymer was further verified using activated SAMSA Fluorescein as a fluorescent thiol probe. Following incubation with activated SAMSA Fluorescein, OPSS-HEP exhibited a distinct fluorescent band, whereas the precursor HEP-NH_2_ produced no detectable fluorescence under identical reaction conditions, confirming successful incorporation of thiol-reactive OPSS groups (Figure S1D). This characterization strategy is consistent with our previously reported methodology for OPSS-HEP synthesis and functional validation.^9,10,15^

A minor higher-molecular-weight band was consistently observed in the OPSS-HEP preparations, corresponding to dimeric HEP–S–S-HEP formed by spontaneous degradation of a small fraction of OPSS groups, which exposes free thiols that subsequently form intermolecular disulfide bonds. This dimeric species typically accounts for approximately 10% of the preparation and is routinely compensated for during LNP surface modification by proportionally increasing the amount of OPSS-HEP used. As expected, the dimeric species exhibited negligible fluorescence after activation with SAMSA Fluorescein labeling because its disulfide linkage does not readily undergo thiol–disulfide exchange on the time scale of the reaction. Together, these results demonstrate that OPSS-HEP retains polymer integrity while providing reactive OPSS groups suitable for covalent conjugation to thiolated LNPs.

Having successfully prepared the surface-modifying polymers, we next developed a panel of LNP formulations using clinically validated ionizable lipids and distinct surface chemistries. Specifically, we used three benchmark ionizable lipids: (i) DLin-MC3-DMA, used in Alnylam Pharmaceuticals’ siRNA therapeutic Onpattro,^23^ (ii) SM-102, used in Moderna’s Spikevax COVID-19 vaccine,^24^ and (iii) ALC-0315, used in the Pfizer-BioNTech Comirnaty COVID-19 vaccine.^25^ In Table S1, we provide a comprehensive comparison of the lipid compositions for the nine LNP formulations established in this study. We further provide the lipid compositions of the corresponding commercial RNA-LNP systems. Figure 1A summarizes schematically the synthesis and surface engineering steps used to prepare the nine different formulations (#1-#9).

**Figure 1:**
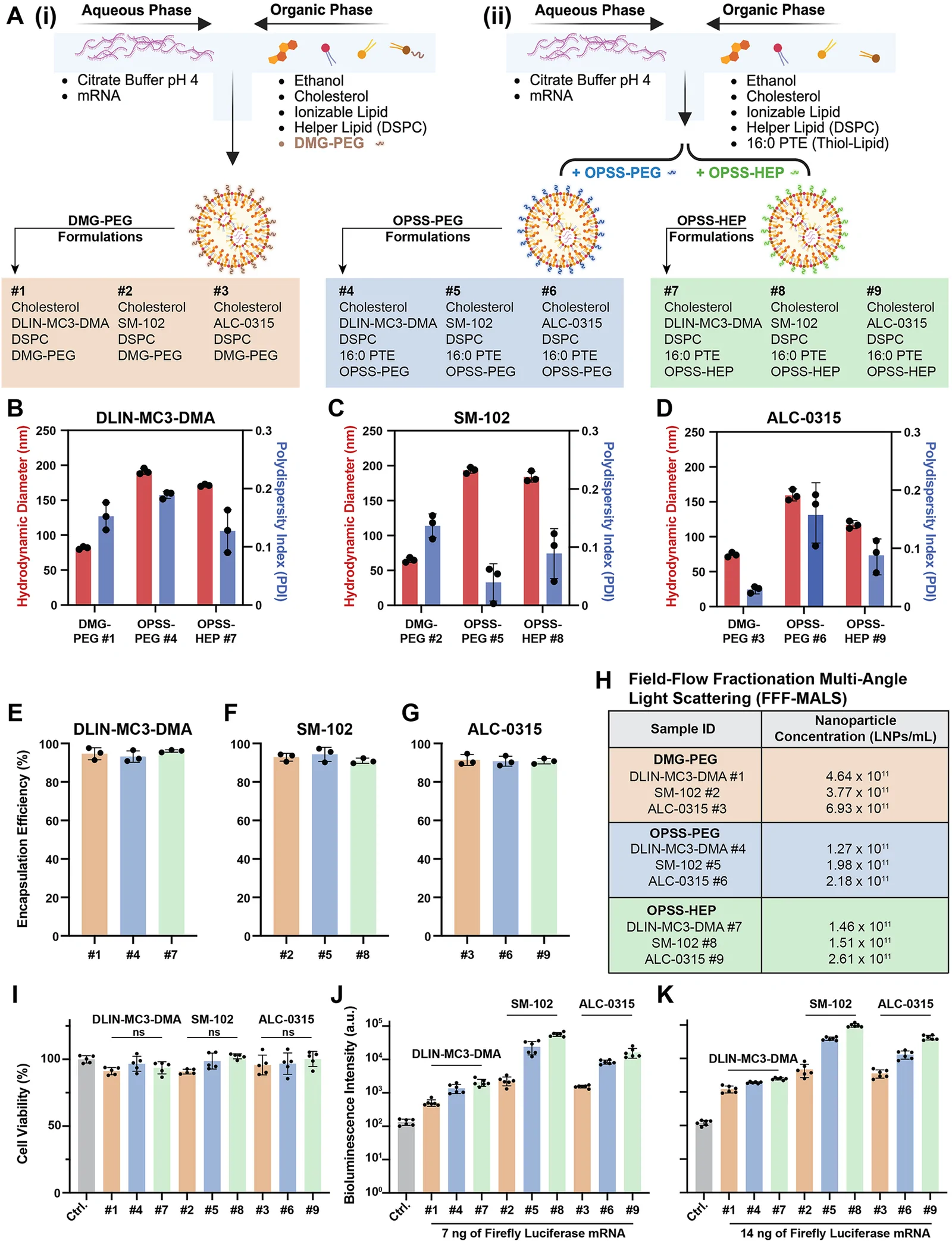
Screening of various lipid nanoparticle (LNP) formulations containing firefly luciferase mRNA. **(A)** Schematic overview of the mRNA-LNP synthesis process for: (i) DMG-PEG modified formulations, and (ii) OPSS-PEG and OPSS-HEP, respectively. A total of nine formulations (#1-#9) were prepared. **(B–D)** Hydrodynamic diameters (HDD) and polydispersity indices (PDI) of mRNA-loaded LNPs prepared with three different ionizable lipids and DMG-PEG, OPSS-PEG, or OPSS-HEP surface modifications. The dynamic light scattering (DLS) data are shown as mean ± SD (n=3). **(E–G)** Quantification of mRNA encapsulation efficiency (%EE) using RiboGreen fluorescence assays. Data are shown as mean ± SD (n=3). (H) Results of field-flow fractionation coupled with multi-angle light scattering (FFF-MALS) to quantify nanoparticle number concentrations (LNPs/mL) for all formulations. **(I)** XTT-based cell viability results of RAW 264.7 murine macrophages treated with mRNA-LNPs (14 ng mRNA) for 24 h. Data represent mean ± SD (n=5). No statistically significant differences were observed among groups (one-way ANOVA, p>0.05). **(J)** Transfection efficiencies based on bioluminescence measurements in RAW 264.7 cells after 24 h incubation with mRNA-LNPs containing 7 ng of firefly luciferase mRNA. Data represent mean ± SD (n=6). One-way ANOVA revealed statistically significant differences. Within the DLIN-MC3-DMA group: OPSS-HEP vs DMG-PEG, p=0.001; OPSS-HEP vs OPSS-PEG, p=0.015. Within the SM-102 group: OPSS-HEP vs DMG-PEG, p<0.0001; OPSS-HEP vs OPSS-PEG, p=0.002. Within the ALC-0315 group: OPSS-HEP vs DMG-PEG, p<0.0001; OPSS-HEP vs OPSS-PEG, p=0.001. **(K)** Transfection efficiencies based on bioluminescence measurements in RAW 264.7 cells after 24 h incubation with mRNA-LNPs containing 14 ng of firefly luciferase mRNA. Data represent mean ± SD (n=6). One-way ANOVA revealed significant differences. Within the DLIN-MC3-DMA group: OPSS-HEP vs DMG-PEG, p=0.0006; OPSS-HEP vs OPSS-PEG, p=0.020. Within the SM-102 group: OPSS-HEP vs DMG-PEG, p<0.0001; OPSS-HEP vs OPSS-PEG, p=0.001. Within the ALC-0315 group: OPSS-HEP vs DMG-PEG, p<0.0001; OPSS-HEP vs OPSS-PEG, p=0.0008. Parts of this Figure were created in BioRender. Wilhelm, S. (2026) https://BioRender.com/v2ur7af.

Dynamic light scattering (DLS) measurements confirmed that all nine formulations exhibited comparable hydrodynamic diameters (HDD <200 nm) and acceptable polydispersity indices (PDI <0.2), indicating uniform nanoparticle populations (Figure S2A–C and Figure 1B–D).^14,26^ While DLS provides robust measurements of hydrodynamic size and colloidal homogeneity, additional cryogenic transmission electron microscopy (cryo-TEM) might provide complementary structural information to further characterize the morphology of OPSS-HEP-modified LNPs in future studies.^1,27^ The mRNA encapsulation efficiencies remained high across all formulations (>90%), as quantified with RiboGreen fluorescence assays (Figure 1E–G). These results confirm that our surface modification process did not compromise mRNA loading efficiency. We further quantified the number concentrations of all formulations using field-flow fractionation multi-angle light scattering (FFF-MALS). The results in Figure 1H demonstrate comparable yields across all formulations. Figure 1I shows the cell viability results using a 2,3-Bis­(2-Methoxy-4-Nitro-5-Sulfophenyl)-2H-Tetrazolium-5-Carboxanilide (XTT)-based assay in RAW 264.7 macrophages. No significant differences in cell viability were observed among formulations at the tested dose (14 ng mRNA per 96-well plate), demonstrating that both PEG- and HEP-modified LNPs were well tolerated.

We next assessed the *in vitro* mRNA delivery efficiencies of these formulations. We incorporated firefly luciferase mRNA into the LNPs and measured the corresponding bioluminescence signals upon incubation with RAW 264.7 macrophages. Across all three ionizable lipid systems, OPSS-HEP-modified LNPs consistently outperformed both DMG-PEG2000 and OPSS-PEG counterparts (Figure 1J-K). This enhancement was observed at two different mRNA doses, indicating robustness across dosing conditions. Notably, while all lipid systems supported functional mRNA delivery, the SM-102 formulations showed the most consistent and robust performance across surface modifications and were selected for subsequent optimization studies.

Based on these results, we selected the SM-102 formulations (#2, #5, and #8) for the subsequent experiments. The zeta potential measurements of these three formulations are shown in Figure S2D, demonstrating a relatively strong negative (−16 mV) surface charge for the HEP-coated LNPs, i.e., formulation #8, compared to the PEGylated counterparts. This result is expected because HEP polysaccharides are polyanionic polymers, while the PEG polymers are not charged and typically exhibit near-neutral zeta potentials.

To further investigate the impact of LNP surface chemistry on mRNA delivery, we systematically varied both the molecular weight and conjugation strategy of the polymer surface ligands. We used OPSS-HEP with molecular weights (MW) of 3.6 kDa, 7 kDa, and 13 kDa; OPSS-PEG with an MW of 10 kDa; and maleimide-HEP with an MW of 13 kDa. All formulations maintained comparable hydrodynamic diameters, narrow size distributions, and minimal cytotoxicity as shown in Figure S2E–F, indicating that these surface modifications did not adversely affect nanoparticle integrity, colloidal stability, and cellular compatibility. We selected 13-kDa maleimide-HEP to investigate whether the surface-modification chemistry affected mRNA delivery performance compared to 13-kDa OPSS-HEP. The maleimide functional group typically forms a more stable linkage with thiols compared to the disulfide exchange reaction of OPSS-HEP.^14,28,29^

Despite exhibiting similar physicochemical characteristics, the formulations displayed substantial differences in functional mRNA delivery (Figure S2). Using RAW 264.7 macrophages as a model system for transfection, we observed that formulations with higher HEP polysaccharide MW exhibited a stronger bioluminescence signal. Additionally, the OPSS-HEP formulations outperformed the maleimide-HEP modifications (Figure S2G–H). Among all tested formulations, LNPs modified with 13-kDa OPSS-HEP consistently produced the relatively highest bioluminescence signals and even outperformed DMG-PEG2000 and OPSS-PEG-based formulations. These findings suggest that both polymer size and surface chemistry may contribute to the mRNA delivery efficacy.

To determine whether free high-MW polysaccharide affected delivery, we selected a 170-kDa HEP lacking a chemical functional group for conjugation to the nanoparticle surface. As shown in Figure S2I–J, the addition of free 170-kDa HEP did not improve firefly luciferase bioluminescence. These results indicate that the enhanced mRNA efficacy observed with HEP-coated mRNA-LNPs arises from the surface display of the HEP polysaccharides.

Collectively, these studies helped identify 13-kDa OPSS-HEP as the candidate surface-modification strategy for subsequent investigations. The LNPs modified with 13-kDa OPSS-HEP exhibited favorable physicochemical properties and cytocompatibility with markedly enhanced mRNA delivery efficacy in macrophages. Additionally, we selected SM-102 as the candidate ionizable lipid for all subsequent studies. To rigorously evaluate the SM-102-based formulations, we included DMG-PEG2000 mRNA-LNPs as formulation controls with clinical and commercial relevance, as they are used in Moderna’s COVID-19 vaccines. Additionally, we selected OPSS-PEG mRNA-LNPs as mechanistic controls to isolate the specific effects of the HEP polysaccharide surface coating.

### Cell-Type-Dependent Effects of Surface Modification on mRNA Delivery Efficacy

Next, we evaluated the impact of LNP surface modification on mRNA delivery across multiple cell types. We selected murine C2C12 myoblasts, DC2.4 dendritic cells, and RAW 264.7 macrophages, as mRNA-LNP-based vaccines often encounter these cell types upon subcutaneous or intramuscular administration *in vivo*.^7^

We prepared mRNA formulations, i.e., formulations #2, #5, and #8, with an N/P ratio of 8 and SM-102 as the ionizable lipid. Figure 2A summarizes the physicochemical properties of all three formulations, demonstrating consistent size and uniformity. The mRNA encapsulation efficiencies remained consistently high (>90%, Figure 2B), and the LNP concentrations measured by FFF-MALS were comparable (Figure 2C). We then assessed the cytocompatibility of the LNP formulations in myoblasts (C2C12), dendritic cells (DC2.4), and macrophages (RAW 264.7). Across all three cell lines, no significant differences in cell viability were observed following treatment with firefly luciferase mRNA-LNPs (140 ng mRNA/96-well plate, 24 h), demonstrating that both PEG- and HEP-modified formulations were well tolerated (Figure 2D–F).

**Figure 2:**
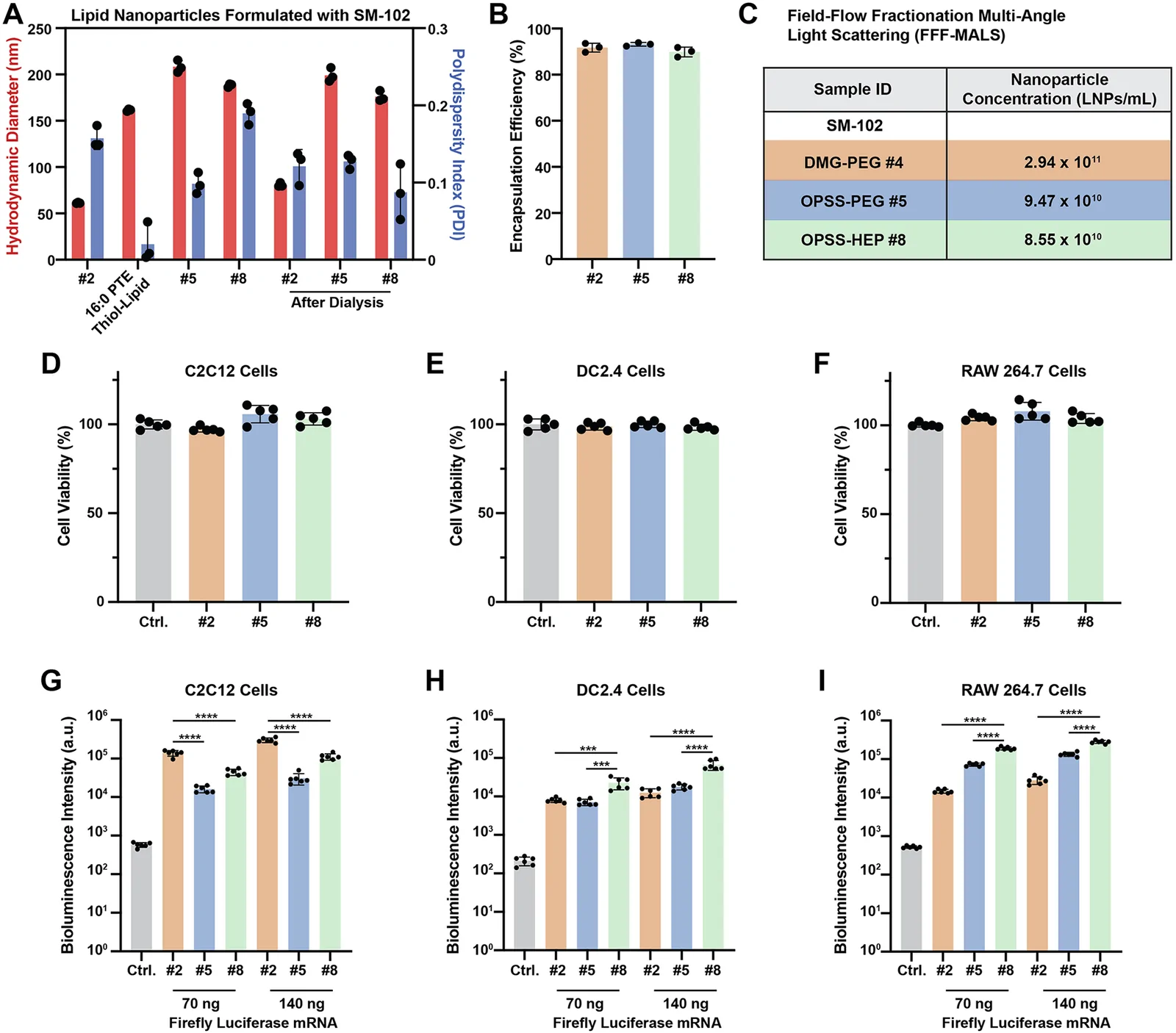
Comparison of mRNA delivery efficiency of LNPs with different surface modifications (DMG-PEG2000, OPSS-PEG, and OPSS-HEP) across three different cell lines. **(A)** HDD and PDI of mRNA-LNP formulations measured by DLS. The data are presented as mean ± SD (n=3). (B) %EE of mRNA in corresponding LNPs determined by RiboGreen assay; mean ± SD (n=3). (C) Lipid nanoparticle concentrations quantified by FFF-MALS. **(D–F)** Cell viability of C2C12, DC2.4, and RAW 264.7 cells treated with mRNA-LNPs (140 ng mRNA) for 24 h, assessed by XTT assay; mean ± SD (n=5). No statistically significant differences were observed (one-way ANOVA, p>0.05). **(G–I)** Transfection efficiency in C2C12, DC2.4, and RAW 264.7 cells following 24 h incubation with mRNA-LNPs at doses of 70 or 140 ng mRNA. Data represent mean ± SD (n=6). Statistical significance was determined by one-way ANOVA; ***p<0.001, ****p<0.0001.

To assess the functional mRNA delivery, we quantified bioluminescence after transfection at two doses, i.e., 70 and 140 ng of firefly luciferase mRNA. In immune cell models, i.e., DC2.4 and RAW 264.7, mRNA-LNPs with OPSS-HEP surface modification consistently showed significantly higher transfection efficiency than DMG-PEG2000 and OPSS-PEG (Figure 2H–I). These results suggest that HEP-based surface modification enhances functional mRNA delivery in APCs. In contrast, in C2C12 myoblasts, PEG-based formulations showed higher bioluminescence signals than HEP-modified LNPs, indicating that the effect of surface chemistry may be cell-type dependent (Figure 2G).

Our findings highlight a differential role for surface modification in regulating functional mRNA delivery across distinct cellular environments. The enhanced performance of HEP-coated LNPs in macrophages and dendritic cells suggests improved interactions with immune cells or enhanced intracellular mRNA processing. In contrast, PEGylation of LNPs appeared more favorable for functional mRNA delivery in myoblasts. Importantly, these differences are not explained by LNP size, mRNA encapsulation efficiency, or cytotoxicity. Instead, HEP-coated LNPs may exhibit distinct uptake mechanisms or intracellular trafficking pathways, thereby improving functional mRNA delivery.

Together, these results demonstrate that HEP-based LNP surface engineering selectively enhances mRNA delivery in immune-relevant cell types, providing a rationale for further mechanistic investigation into cell uptake and intracellular trafficking. Interestingly, HEP-modified LNPs enhanced mRNA expression in the APC lines DC2.4 and RAW 264.7 but not in C2C12 myoblasts, suggesting that the advantages conferred by HEP surface engineering may be cell-type dependent. APCs and myoblasts differ substantially in their cell-surface receptor repertoire, glycocalyx composition, endocytic activity, and intracellular trafficking pathways, all of which can influence nanoparticle recognition, internalization, and intracellular processing. In particular, professional APCs, including macrophages and dendritic cells, express diverse pattern-recognition and phagocytic receptors that mediate nanoparticle recognition and internalization, potentially contributing to cell-type-dependent interactions with surface-modified LNPs.^30^ Furthermore, differences in endosomal maturation dynamics and membrane composition between these cell types may alter the intracellular fate of internalized LNPs and ultimately affect productive mRNA delivery.^31,32^ While the present study demonstrates enhanced transfection of HEP-modified LNPs in APCs, additional studies directly comparing cellular uptake, endocytic pathways, and intracellular trafficking in C2C12 cells will be valuable to determine the mechanisms underlying this cell-type-specific response. This finding indicates that the biological effects of HEP surface engineering are cell-type dependent, although the underlying mechanisms remain to be determined.

### Lipid Nanoparticle Surface Modification Modulates Intracellular Trafficking Rather Than Cell Uptake Efficiency

Next, we studied whether the enhanced mRNA translation efficacy observed with HEP-modified LNPs resulted from increased cellular uptake or altered intracellular trafficking and processing. First, we prepared luciferase mRNA-LNP formulations, i.e., formulations #2, #5, and #8, containing lipophilic Vybrant 3,3’-Dioctadecyloxacarbocyanine Perchlorate (DiO) fluorescent labels. We selected these DiO labels to enable confocal laser scanning microscopy (CLSM) imaging and quantitative analysis of nanoparticle internalization in RAW 264.7 macrophages. Secondly, we prepared similar formulations without DiO, but containing enhanced green fluorescent protein (EGFP) mRNA. We characterized the physicochemical properties of the formulations as shown in Figures S3–S4). Finally, we then incubated RAW 264.7 macrophages for 24 h with the formulations and performed CLSM imaging.

As shown by the representative CLSM images in Figures 3A-B and S5A-B, the DiO-labeled luciferase mRNA-LNPs were efficiently internalized by the RAW 264.7 macrophages across a range of LNP and mRNA concentrations. We observed consistent intracellular localization patterns across all formulations, suggesting that LNP surface modification did not substantially alter nanoparticle uptake by these cells. Using quantitative image analysis, we found no statistically significant differences in DiO signals among the three LNP formulations tested, indicating comparable cellular uptake efficiencies regardless of LNP surface chemistry (Figures 3C and S5C).

**Figure 3:**
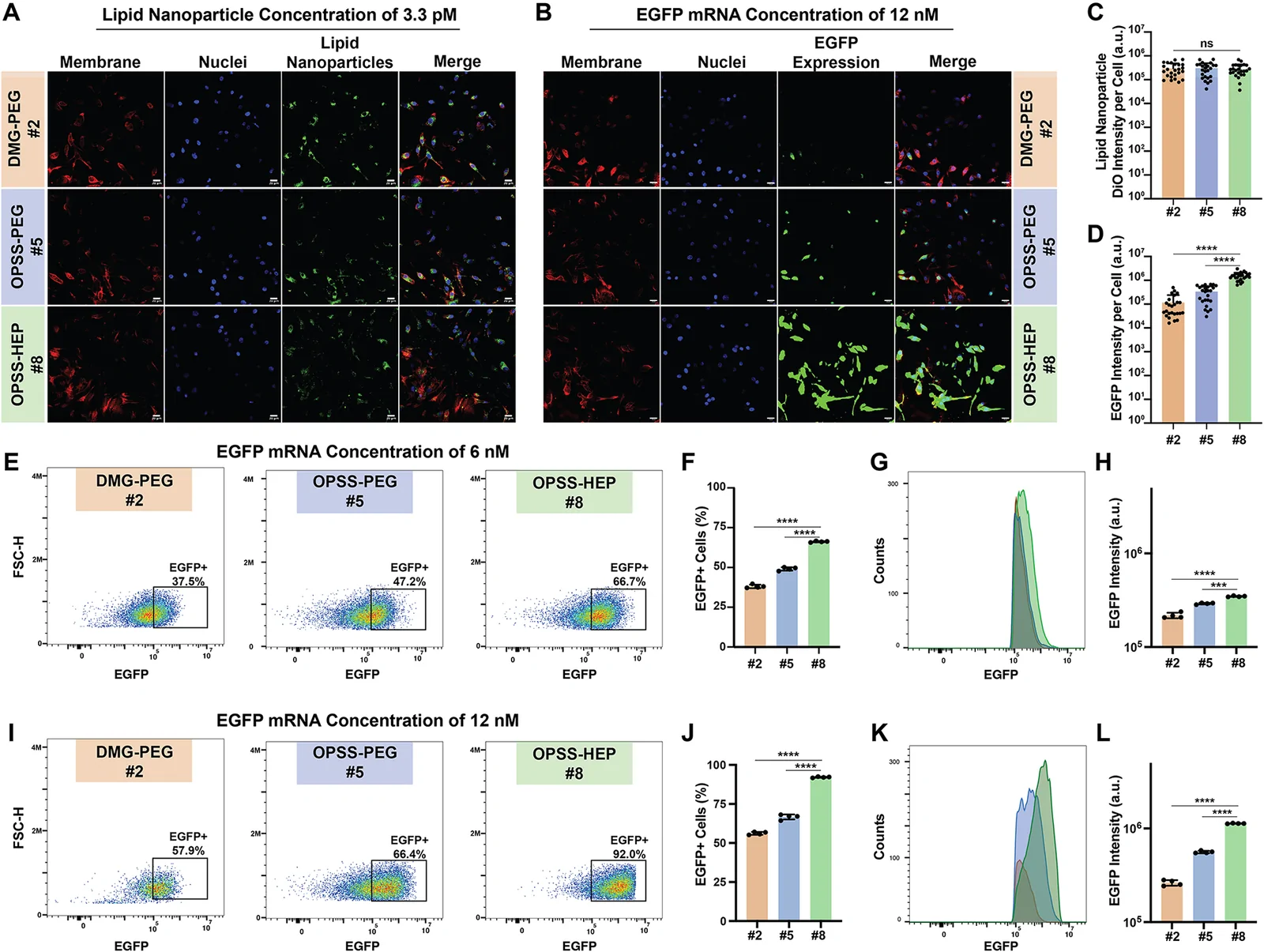
LNP surface modification affects mRNA delivery efficacy but not cellular uptake of mRNA-LNPs. **(A)** Representative confocal laser scanning microscopy (CLSM) images of RAW 264.7 murine macrophages following 24 h incubation with Vybrant DiO-labeled DMG-PEG2000-, OPSS-PEG-, or OPSS-HEP-modified mRNA-LNPs (3.3 pM LNPs). The DiO-LNPs signal is shown in green, cell membranes were stained with WGA-CF633 (red), and cell nuclei with DAPI (blue). Scale bars represent 20 μm. (B) Representative CLSM images of RAW 264.7 cells transfected for 24 h with DMG-PEG2000-, OPSS-PEG-, or OPSS-HEP-modified EGFP mRNA-LNPs (12 nM mRNA). EGFP expression is shown in green. Scale bars represent 20 μm. (C) Quantification of cellular uptake based on DiO fluorescence intensity of panel A. Data represent mean ± SD (n=25 cells; one-way ANOVA). No significant differences were observed (p>0.05). (D) Quantification of EGFP fluorescence intensity of panel B. Data represent mean ± SD (n=25 cells; one-way ANOVA; ****p<0.0001). **(E–L)** Flow cytometry results quantifying EGFP expression in RAW 264.7 cells following transfection with surface-modified EGFP mRNA-LNPs at 6 nM and 12 nM mRNA, respectively. **(E, I)** Representative flow cytometry plots showing EGFP-positive cell populations. **(F, J)** Percentage of EGFP-positive cells. (G, K) Representative overlay histograms of EGFP fluorescence intensity distributions. **(H, L)** Mean EGFP fluorescence intensity per cell population. Data represent mean ± SD (n=4). Statistical significance was determined by one-way ANOVA with multiple comparisons (***p<0.001, ****p<0.0001).

In contrast, when we evaluated the corresponding EGFP expression, we observed substantial differences in the functional delivery of mRNA. Cells incubated with OPSS-HEP-modified EGFP mRNA-LNPs exhibited greater EGFP fluorescence than DMG-PEG2000- and OPSS-PEG-modified counterparts (Figures 3B and S5B). We confirmed this qualitative observation with quantitative image analysis of EGFP fluorescence (Figures 3D and S5D).

Despite comparable nanoparticle uptake, the HEP-modified mRNA-LNPs elicited substantially greater EGFP expression, indicating that efficient cellular internalization alone is insufficient for effective mRNA delivery. Instead, our findings suggest that the superior performance of HEP-coated mRNA-LNPs may stem from enhanced intracellular processing, resulting in a larger fraction of delivered mRNA available for downstream cytosolic translation.

To determine whether the enhanced delivery observed in macrophages could be extended to other professional APCs, we transfected DC2.4 dendritic cells. Consistent with our results in RAW 264.7 macrophages, OPSS-HEP-modified mRNA-LNPs yielded substantially higher EGFP expression (Figure S6). Quantitative image analysis confirmed significantly higher EGFP fluorescence intensity in the HEP group, demonstrating that the enhanced delivery effect of HEP polysaccharide surface engineering is not limited to a single cell type but also persists in dendritic cells.

To further validate our findings, we used flow cytometry to quantify transfection efficiency in cells treated with EGFP mRNA-loaded LNPs (Figure S7). Consistent with our CLSM imaging results, OPSS-HEP-modified mRNA-LNPs showed higher percentages of EGFP-positive cells (Figure 3E–L). Furthermore, HEP-coated mRNA-LNPs produced substantially higher mean EGFP fluorescence intensity, indicating enhanced protein expression on a per-cell basis.

The discrepancy between nanoparticle uptake and protein expression is particularly noteworthy for the DMG-PEG2000-based mRNA-LNP formulations. Despite exhibiting uptake levels comparable to those of HEP-coated mRNA-LNPs (Figure 3A), the DMG-PEG2000 mRNA-LNPs generated substantially lower EGFP expression, indicating limited functional mRNA delivery. These findings suggest that, within APCs, conventional PEGylated LNPs may undergo intracellular sequestration following cell uptake.^33^

Although HEP-coated mRNA-LNPs enter cells with similar efficiency, the enhanced delivery of functional mRNA leads to significantly higher protein expression. Collectively, these findings decouple cellular uptake from functional delivery. This observation motivated us to further investigate the intracellular fate of mRNA-LNPs using live-cell imaging.

### Lipid Nanoparticles Surface-Modified with Heparosan Polysaccharides Exhibit Reduced Endosomal Retention and Enhanced Functional mRNA Delivery

To investigate the intracellular fate of the HEP-coated mRNA-LNPs, we performed live-cell imaging studies. First, we prepared EGFP mRNA-LNPs, i.e., formulations #2, #5, and #8, containing lipophilic 1,1’-dioctadecyl-3,3,3’,3’-tetramethylindodicarbocyanine, 4-Chloro-benzenesulfonate salt (DiD) fluorescent labels (Figure S8). Additionally, we transfected cells with plasmids encoding mCherry-tagged endosomal markers (Figure 4A). We verified the successful expression of endosomal markers Rab5 (early endosomes) and Rab7 (late endosomes) labeled with mCherry using live-cell CLSM imaging (Figure 4B–C).^31,32^

**Figure 4:**
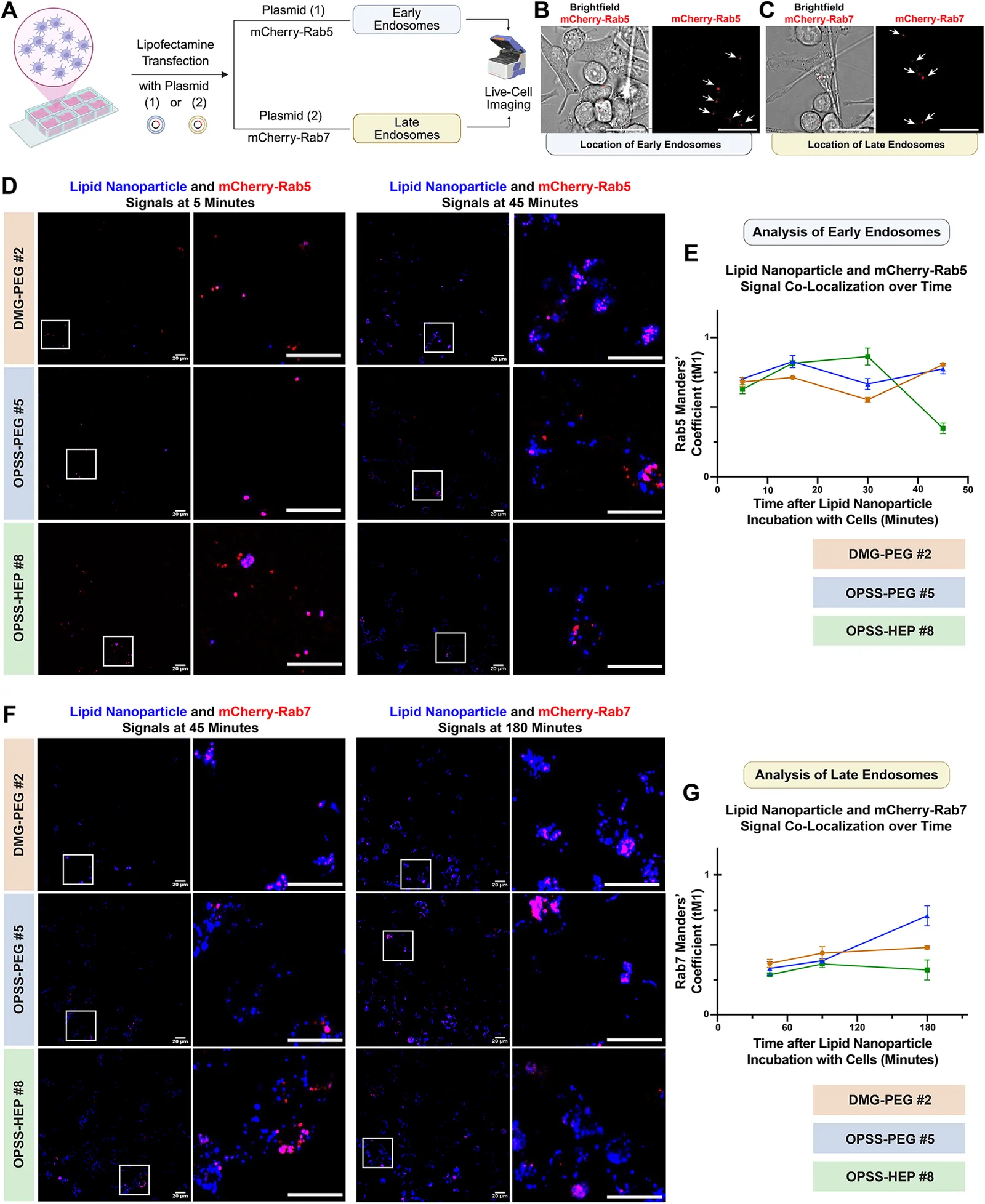
Live-cell imaging and intracellular localization of DiD-labeled EGFP mRNA-LNPs with different surface modifications in DC2.4 cells during early and late endosomal transport. (A) Schematic illustration of the live-cell imaging workflow. DC2.4 murine dendritic cells were transiently transfected with plasmids encoding Rab5-mCherry (early endosome marker) or Rab7-mCherry (late endosome marker) and cultured for 48 h prior to imaging. Cells were subsequently treated with DiD-labeled EGFP mRNA-LNPs modified with DMG-PEG2000 (#2), OPSS-PEG (#5), or OPSS-HEP (#8). Cell uptake and intracellular trafficking were monitored using live-cell spinning-disk CLSM. (B) Representative CLSM images demonstrating successful expression of Rab5-mCherry, with early endosomes visualized as discrete red puncta. Scale bars represent 20 μm. (C) Representative images demonstrating successful expression of Rab7-mCherry, with late endosomes visualized as discrete red puncta. Scale bars represent 20 μm. (D) Representative CLSM images of DC2.4 cells expressing Rab5-mCherry following treatment with DiD-labeled EGFP mRNA-LNPs. Images were acquired at 5 and 45 min post-treatment with the various LNP formulations. Scale bars represent 20 μm. (E) Quantification of colocalization between DiD-labeled mRNA-LNPs and Rab5-mCherry-labeled early endosomes from 5 to 45 min post-treatment using Manders’ colocalization coefficient (tM1). Data represent mean ± SD (n=3). The solid lines are a guide to the eye. (F) Representative CLSM images of DC2.4 cells expressing Rab7-mCherry following treatment with DiD-labeled EGFP mRNA-LNPs. Images were acquired at 45 and 180 min post-treatment. Scale bars represent 20 μm. (G) Quantification of colocalization between DiD-labeled mRNA-LNPs and Rab7-mCherry-labeled late endosomes from 45 to 180 min post-treatment using Manders’ colocalization coefficient (tM1). Data represent mean ± SD (n=3). The solid lines are a guide to the eye.

As shown in Figure 4D-E, all tested formulations exhibited rapid cellular internalization and early colocalization with mCherry-Rab5-labeled early endosomes within minutes after treatment, indicating that the LNP surface modification did not substantially alter initial cell uptake. Figures S9 and S10 show additional representative live-cell images of mCherry-Rab5- positive early endosomal trafficking at 5, 15, 30, and 45 min after cells were incubated with the various mRNA-LNP formulations. The images demonstrate that over time the nanoparticles entered the canonical endosomal maturation pathway, transitioning from mCherry-Rab5-positive early endosomes to mCherry-Rab7-positive late endosomes (Figure 4F-G).

Next, we performed quantitative image analysis with Manders’ colocalization coefficient (tM1) to assess the colocalization of DiD (label for LNPs) and mCherry (label for endosomal markers) signals, thereby studying the distinct intracellular trafficking behaviors among the formulations.^34^ As shown in Figure 4E, the OPSS-HEP-modified mRNA-LNPs exhibited rapid colocalization with mCherry-Rab5-positive early endosomes immediately after cell uptake, followed by a pronounced decline in mCherry-Rab5 overlap at ∼30–45 min (Figure 4E). Importantly, the decreased colocalization with mCherry-Rab5 was not accompanied by a corresponding increase in mCherry-Rab7 association (Figure 4G). Instead, OPSS-HEP-modified mRNA-LNPs also exhibited a marked decrease in mCherry-Rab7 colocalization ∼3 h post-treatment, resulting in significantly lower mCherry-Rab7 colocalization compared to the PEG-coated counterpart formulations (Figure 4G). This trafficking pattern suggests that a substantial fraction of HEP-coated mRNA-LNPs may escape the endosomal pathway during the critical transition between early and late endosomal maturation rather than progressing to lysosomal degradation.^32^

In contrast, the PEG-coated mRNA-LNP formulations exhibited sustained, or even progressive, colocalization with mCherry-Rab7-positive late endosomes over time (Figure 4G). The persistent accumulation of PEGylated LNPs in mCherry-Rab7-positive compartments indicates prolonged residence in the endolysosomal network, where nanoparticles are exposed to increasingly acidic conditions and enzymatic degradation. These observations suggest that although PEG-coated LNPs are efficiently internalized by dendritic cells, a substantial proportion of the nanoparticles remain trapped within endosomes, and this endosomal entrapment limits the functional delivery of mRNA into the cytosol for downstream translation. Figures S11 and S12 provide additional representative live-cell imaging data of mCherry-Rab7-positive late endosomal trafficking in DC2.4 dendritic cells.

We further verified the successful expression of LAMP1 as a lysosomal marker labeled with mCherry using live-cell CLSM imaging (Figure 5A-B). Notably, OPSS-HEP-modified mRNA-LNPs displayed significantly reduced overlap with mCherry-LAMP1-positive compartments, indicating decreased endolysosomal retention and an increased likelihood of endosomal escape (Figure 5C–D).^17,18,35^ The simultaneous reduction in mCherry-Rab5 and mCherry-Rab7 colocalization suggests that HEP-coated mRNA-LNPs may escape from the endosomal network before completion of Rab7-dependent maturation.^32^ A potential explanation may be that the HEP polysaccharides and ionizable lipids promote transient endosomal membrane destabilization and facilitate cytosolic release of the encapsulated mRNA cargo. Alternatively, the HEP polysaccharides may affect the endosomal membrane curvature and budding dynamics, potentially increasing the frequency of budding-and-collapse escape events during endosomal maturation, as suggested by the vesicle-budding and collapse (VBC) mechanism.^36,37^ Figures S13–S15 provide additional representative live-cell images of mCherry-LAMP1-positive lysosomal trafficking.^17,31,38^

**Figure 5:**
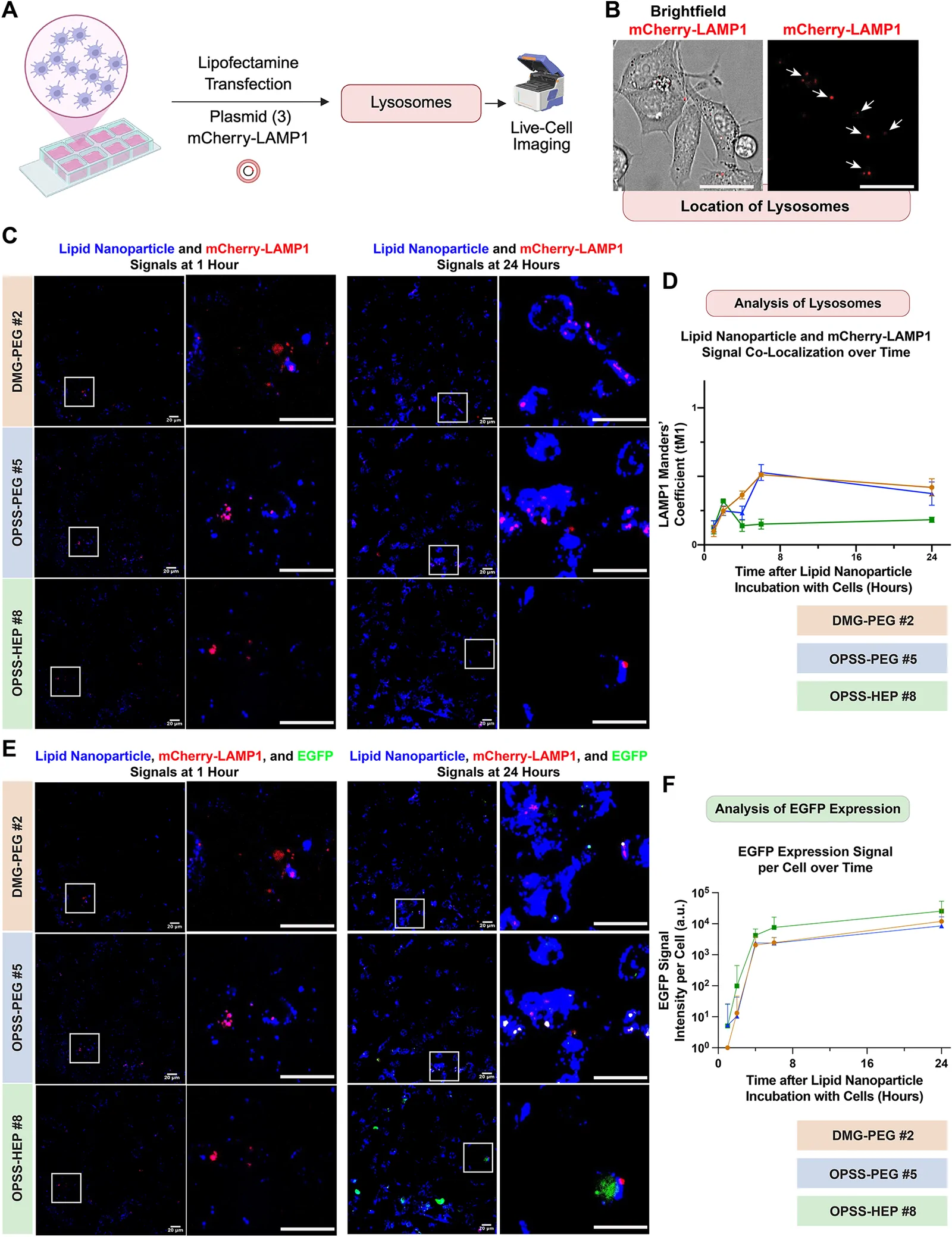
Live-cell imaging and intracellular fate of DiD-labeled EGFP mRNA-LNPs with different surface modifications in DC2.4 cells during lysosomal transport. (A) Schematic illustration of the live-cell imaging workflow. DC2.4 murine dendritic cells were transiently transfected with a plasmid encoding mCherry-LAMP1 (lysosomal marker) and cultured for 48 h prior to imaging. Cells were subsequently treated with DiD-labeled EGFP mRNA-LNPs modified with DMG-PEG2000, OPSS-PEG, or OPSS-HEP, and intracellular trafficking was monitored by live-cell CLSM for up to 24 h. (B) Representative CLSM images demonstrating successful expression of mCherry-LAMP1, with lysosomes visualized as discrete red puncta. Scale bars represent 20 μm. (C) Representative CLSM images of DC2.4 cells expressing mCherry-LAMP1 following treatment with DiD-labeled EGFP mRNA-LNPs. The CLSM images were acquired at 1 and 24 h post-treatment and show lysosomes (red), DiD-labeled mRNA-LNPs (blue), and merged channels illustrating DiD-mCherry colocalization. Scale barss represent 20 μm. (D) Quantification of colocalization between DiD-labeled mRNA-LNPs and mCherry-LAMP1-positive lysosomes from 1 to 24 h post-treatment using Manders’ colocalization coefficient (tM1). Data represent mean ± SD (n=3). (E) Representative CLSM images of DC2.4 cells expressing mCherry-LAMP1 following treatment with DiD-labeled EGFP mRNA-LNPs. The CLSM images were acquired at 1 and 24 h post-treatment and show lysosomes (red), translated EGFP (green), DiD-labeled mRNA-LNPs (blue), and merged channels illustrating the relationship between intracellular trafficking and mRNA expression. Scale bars represent 20 μm. (F) Time-dependent EGFP expression in DC2.4 cells over 24 h following transfection with DMG-PEG2000-, OPSS-PEG-, or OPSS-HEP-modified EGFP mRNA-LNPs. Data represent mean ± SD (n=20). The solid lines are a guide to the eye.

Specifically, early endosomal escape may be driven by a VBC mechanism. Upon entering early endosomes, HEP-coated LNPs may induce localized membrane curvature, resulting in the formation of LNP-containing membrane buds.^37^ Rather than continuing through the normal maturation pathway toward Rab7-positive late endosomes and LAMP1-positive lysosomes, these budded vesicles may undergo controlled destabilization or collapse, releasing the formulations and their encapsulated mRNA into the cytosol.^37^

The VBC mechanism may explain the pronounced decrease in mCherry-Rab5 colocalization at ∼45 min, the relatively low colocalization with mCherry-Rab7 at ∼3 h, and the limited lysosomal colocalization we observed throughout the experiment. While more research is needed to uncover the specific mechanistic steps, our observed behavior of HEP-coated mRNA-LNPs could be favorable for mRNA and other nucleic acid therapeutics. Early cytosolic delivery of payloads can maximize the fraction of delivered mRNA available for effective translation into protein.^39^

Although the present study demonstrates distinct intracellular trafficking behaviors between PEG- and HEP-modified LNPs, the specific endocytic pathways responsible for cellular uptake were not directly investigated. In our previous work with HEP-coated gold nanoparticles, pharmacological inhibitor studies suggested that they are primarily internalized via clathrin-mediated endocytosis and macropinocytosis.^9,10^ However, because endocytic pathways are influenced by nanoparticle composition, surface chemistry, and cell type, these findings cannot be directly extrapolated to OPSS-HEP-modified LNPs.^2,19–21^ Future studies employing complementary approaches, including pharmacological inhibition and genetic perturbation of endocytic pathways, will be valuable for defining the uptake mechanisms of HEP-modified LNPs and their relationship to intracellular trafficking and mRNA delivery.

In addition, all in vitro experiments were performed in complete medium supplemented with 10% FBS, and differences in protein corona composition may be another contributing factor. Previous studies have demonstrated that protein corona formation can influence nanoparticle recognition, intracellular trafficking, and mRNA delivery,^40^ and our prior work showed that HEP coatings reduce nonspecific serum protein adsorption relative to PEG-coated nanoparticles.^10^ Therefore, the altered trafficking observed in the present study may reflect intrinsic effects of HEP surface chemistry, protein corona-mediated interactions, or a combination of both. Future studies integrating quantitative protein corona characterization with uptake pathway analysis and intracellular trafficking measurements will be valuable for defining the relative contributions of these mechanisms.

Importantly, our observed differences in nanoparticle intracellular trafficking closely correlated with functional mRNA delivery outcomes. As shown in Figure 5E-F, EGFP expression increased over time across all treatment groups but was consistently higher in cells treated with HEP-modified EGFP mRNA-LNPs. Detectable EGFP expression emerged at ∼4 h and progressively increased over 24 h (Figure 5F). Corresponding bright-field images are provided in Figure S16 to facilitate visualization of cellular morphology and nuclear boundaries. Time-lapse CLSM imaging further revealed the dynamic intracellular redistribution of OPSS-HEP-modified LNPs throughout the 24-h observation period.

To determine whether the intracellular trafficking behavior observed in DC2.4 dendritic cells was conserved across professional APC populations, we conducted additional live-cell imaging studies using RAW 264.7 macrophages (Figures S17–S23). As in DC2.4 cells, OPSS-HEP-modified mRNA-LNPs showed reduced colocalization with mCherry-Rab5-positive early endosomes, mCherry-Rab7-positive late endosomes, and mCherry-LAMP1-positive lysosomes over time compared with DMG-PEG2000- and OPSS-PEG-modified mRNA-LNP formulations. Correspondingly, HEP-coated mRNA-LNPs produced enhanced time-dependent EGFP expression relative to PEG-modified counterpart formulations (Figure S23).

Collectively, our findings show that altered intracellular trafficking and reduced endolysosomal retention of HEP polysaccharide-coated mRNA-LNP formulations are not limited to dendritic cells but also persist in macrophages. Our live-cell imaging results demonstrate that HEP-based surface engineering improves mRNA delivery by modulating the intracellular fate of LNPs. By promoting escape during the early-to-late endosomal transition and reducing accumulation in Rab7-positive and LAMP1-positive compartments, HEP-coated LNP formulations convert cellular uptake into effective mRNA translation in professional APCs, such as dendritic cells and macrophages.

### Repeated in vivo Administration of Heparosan Polysaccharide Surface Engineering of mRNA-Lnps Maintains Robust mRNA expression

To evaluate the impact of surface modification on in vivo mRNA delivery, we conducted longitudinal bioluminescence imaging after repeated subcutaneous administration of various firefly luciferase mRNA-LNP formulations to C57BL/6 mice. The physicochemical properties of the DMG-PEG2000-, OPSS-PEG-, and OPSS-HEP-modified firefly luciferase mRNA-LNPs used for the in vivo IVIS studies were characterized at an N/P ratio of 8 (Figure S24). The mice received six subcutaneous injections over 76 days (Days 0, 3, 10, 66, 69, and 76), and whole-body bioluminescence was measured 6 h post-injection to assess acute protein expression (Figure 6A). All formulations produced detectable bioluminescence signals localized primarily at the injection site, consistent with subcutaneous delivery (Figure 6B,E). During the initial dosing cycle (Days 0–10), the total luminescence flux progressively decreased with repeated administration across all groups (Figure 6C,F), suggesting reduced expression efficiency over time. Notably, OPSS-HEP-modified mRNA-LNPs exhibited a trend toward higher luminescence signals than PEG-based formulations during the initial dosing cycle. However, these data should be interpreted as evidence of enhanced expression following repeated administration rather than as evidence of prolonged or sustained mRNA expression.

**Figure 6:**
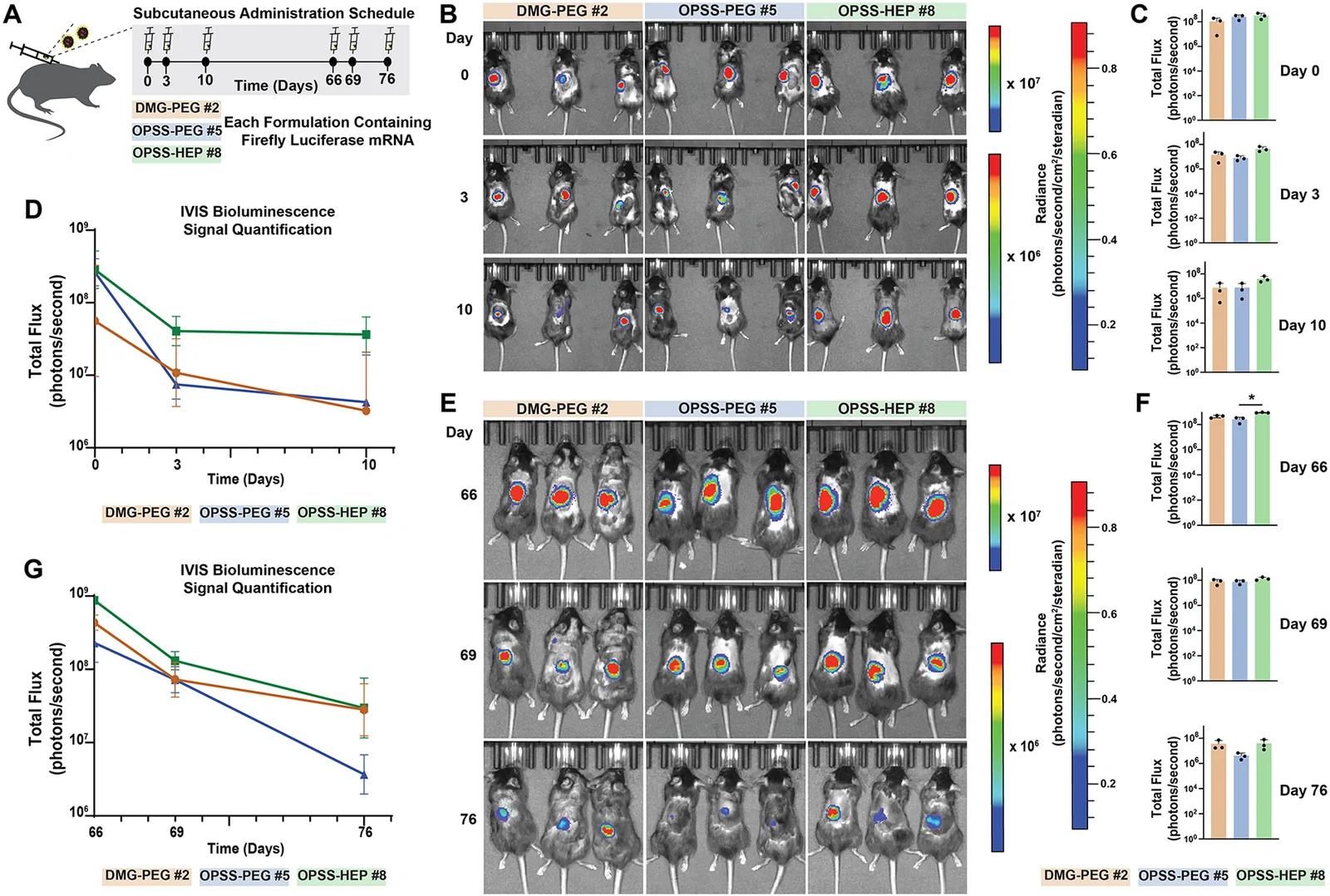
*In vivo* bioluminescence imaging using C57BL/6 mice following repeated subcutaneous administration of surface-modified firefly luciferase mRNA-LNPs over 76 days. **(A)** Subcutaneous administration and dosing schedule. Female C57BL/6 mice (n=3) received subcutaneous injections of firefly luciferase mRNA-LNPs modified with DMG-PEG2000 (#2), OPSS-PEG (#5), or OPSS-HEP (5) on Days 0, 3, 10, 66, 69, and 76 (2 μg of mRNA in 100 μL of 1xPBS). **(B, E)** Representative whole-body bioluminescence images acquired 6 h after each administration using an IVIS SpectrumCT imaging system. Color scales represent radiance intensity. **(C, F)** Quantification of total photon flux corresponding to panels B and E. Data represent mean ± SD (n=3). **(D, G)** Bioluminescence signals illustrating changes in luciferase expression following repeated administration during the first (Days 0–10) and second (Days 66–76) dosing cycles. Data represent mean ± SD (n=3). The solid lines are a guide to the eye. Image acquisition and quantitative analysis were performed using Living Image software. Statistical significance was determined by one-way ANOVA with multiple comparisons (*p<0.05).

After a prolonged interval, a second dosing cycle (Days 66–76) revealed a distinct response pattern. Upon re-administration at Day 66, bioluminescence levels recovered to values comparable to those after the initial dose (Day 0), followed again by a gradual decrease with subsequent injections (Figure 6D,G). This “reset” in expression suggests that the reduced signal observed during the initial phase is not due to a permanent loss of delivery capability, but rather reflects a transient, time-dependent modulation of LNP performance in vivo.^41–43^

OPSS-HEP-modified mRNA-LNPs exhibited bioluminescence signals comparable to those of DMG-PEG2000- and OPSS-PEG-modified mRNA-LNPs throughout the second dosing cycles, with a trend toward higher expression at several measured 6 h post-dose time points. Although all formulations produced detectable luciferase expression at the measured 6 h post-dose time point following each administration, the bioluminescence signal decreased across successive injections. OPSS-HEP-modified mRNA-LNPs consistently exhibited numerically higher bioluminescence signals than DMG-PEG2000- and OPSS-PEG-modified formulations at several measured time points during both dosing cycles. However, because bioluminescence was assessed only at a single 6 h time point after each administration, these data reflect expression at the measured post-dose time point rather than the duration of mRNA expression. The observed differences may be influenced by multiple factors, including local retention, protein corona formation, and immune recognition at the injection site, although their relative contributions remain to be determined.

Throughout the repeated-dosing study, no abnormal changes in body weight were observed in mice receiving DMG-PEG2000-, OPSS-PEG-, or OPSS-HEP-modified firefly luciferase mRNA-LNPs (Figure S25). Body weights remained stable over the 76-day period, suggesting that all formulations were well tolerated under the dosing conditions examined.

These results demonstrate that the major bioluminescence signal remained localized near the injection site and that LNP surface chemistry influences in vivo mRNA expression following repeated administration. Although overall expression levels were comparable among the formulations, HEP-modified mRNA-LNPs maintained mRNA expression levels comparable to those of PEG-modified formulations, demonstrating that replacing PEG with HEP does not compromise repeated-dose in vivo delivery.

### Heparosan Polysaccharide Surface Engineering of mRNA-Lnps Enhances Antigen Expression While Maintaining Immune Homeostasis and Tissue Safety

To evaluate the translational relevance of HEP polysaccharide-based surface engineering of mRNA-LNP formulations, we next assessed delivery of SARS-CoV-2 spike S-2P mRNA, a benchmark in the mRNA-LNP field, using both *in vitro* antigen expression and *in vivo* immune response models (Figure 7A). Prior to vaccination studies, we characterized the various SARS-CoV-2 mRNA-LNP formulations as shown in Figures S26–S27. No significant differences in RAW 264.7 cell viability were observed across formulations (Figure S26D). We then used ELISA to quantify the SARS-CoV-2 spike S-2P production after treatment with surface-modified mRNA-LNP formulations. The OPSS-HEP-coated mRNA-LNPs treated groups exhibited significantly higher spike protein expression than the DMG-PEG2000 and OPSS-PEG counterpart mRNA-LNP formulations (Figure 7B).

**Figure 7:**
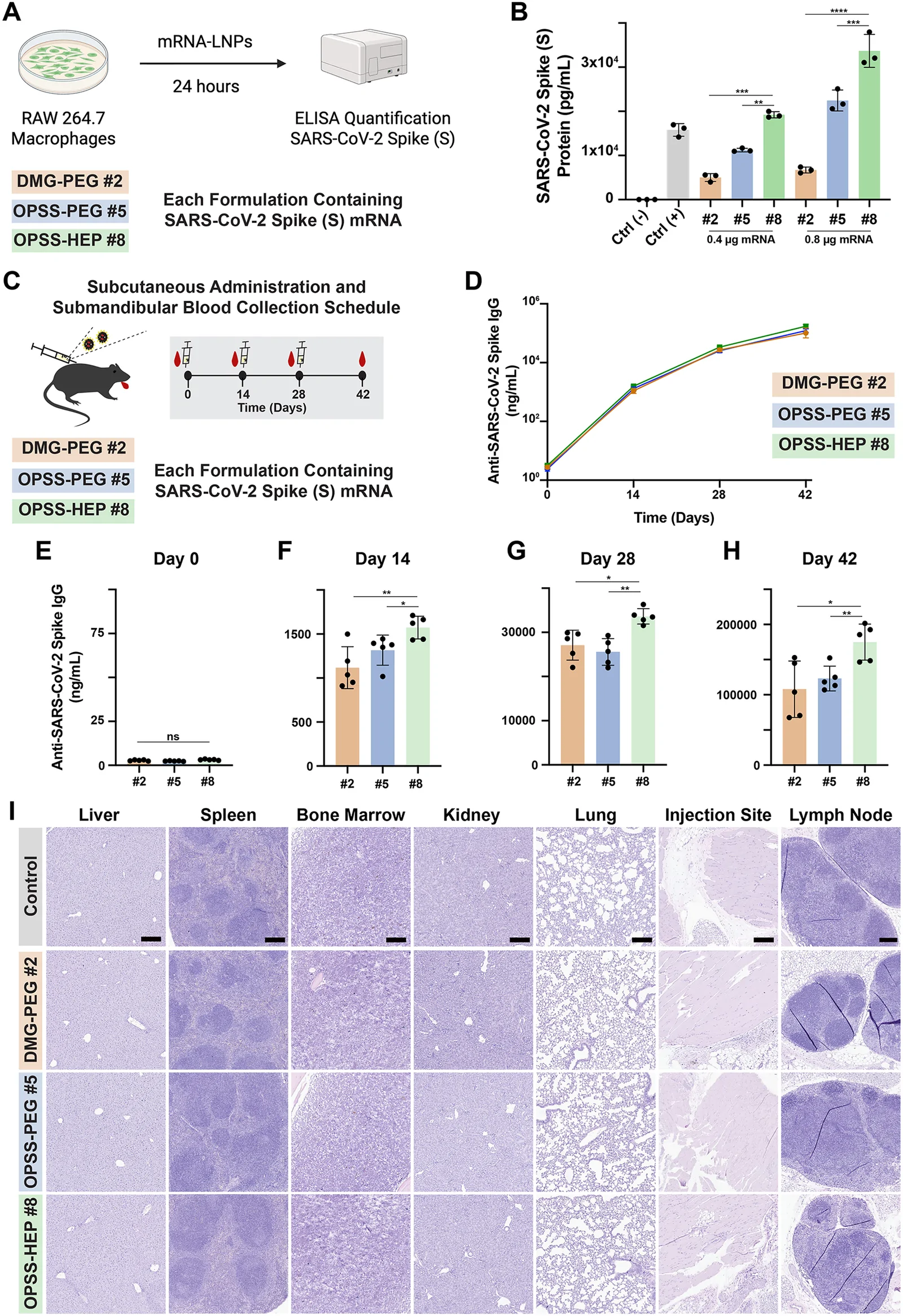
*In vitro* expression, *in vivo* immunogenicity, and histopathological evaluation of SARS-CoV-2 spike S-2P mRNA-LNPs with different surface modifications. **(A)** Schematic illustration of the experimental workflow, including *in vitro* antigen expression studies of DMG-PEG2000-, OPSS-PEG-, and OPSS-HEP-modified SARS-CoV-2 spike S-2P mRNA-LNPs. (B) *In vitro* expression of SARS-CoV-2 spike (S) protein in RAW 264.7 macrophages following 24 h incubation with mRNA-LNPs. Spike protein levels were quantified by ELISA using combined cell lysate and culture supernatant fractions. Lipofectamine-mediated transfection (2 μg mRNA) served as a positive control. Data represent mean ± SD (n=3). Statistical significance was determined by one-way ANOVA with multiple comparisons. (**p<0.01; ***p<0.001; ****p<0.0001) (C) Schematic illustration of the *in vivo* immunization and blood collection schedule. Female C57BL/6 mice (n=5) received subcutaneous injections of SARS-CoV-2 spike S-2P mRNA-LNPs (2 μg mRNA in 100 μL 1xPBS) on Days 0, 14, and 28. Blood samples were collected via the submandibular vein on Days 0, 14, 28, and 42 for serological analysis. (D) Time-dependent serum anti–SARS-CoV-2 spike IgG responses following immunization with surface-modified mRNA-LNP formulations. Data represent mean ± SD (n=5). **(E–H)** Quantification of serum anti-spike IgG levels on Days 0, 14, 28, and 42, respectively. Data represent mean ± SD (n=5). Statistical significance was determined by one-way ANOVA with multiple comparisons. (*p<0.05; **p<0.01; ns=not significant) **(I)** Representative hematoxylin and eosin (H&E)-stained sections of major tissues collected following three subcutaneous administrations of SARS-CoV-2 spike S-2P mRNA-LNPs at 2-week intervals. Mice received a cumulative dose of 6 μg mRNA (2 μg per dose, 100 μL 1xPBS) formulated as LNPs with DMG-PEG2000 (#2), OPSS-PEG (#5), and OPSS-HEP (#8), respectively. Representative tissues include liver, spleen, bone marrow, kidney, lung, injection site, and draining left axillary lymph node. Scale bars: liver, 200 μm; spleen, 200 μm; bone marrow, 100 μm; kidney, 200 μm; lung, 200 μm; injection site, 200 μm; lymph node, 100 μm.

Next, we evaluated *in vivo* immune responses following subcutaneous administration of spike mRNA-LNPs in C57BL/6 mice. We administered three subcutaneous injections (Days 0, 14, and 28) and collected serum samples longitudinally to monitor the anti-SARS-CoV-2 spike IgG production (Figure 7C). All formulations induced a time-dependent increase in antigen-specific IgG levels, demonstrating effective *in vivo* delivery and immune responses (Figure 7D).

Our quantitative analysis revealed that OPSS-HEP-modified mRNA-LNPs elicited significantly higher anti-SARS-CoV-2 spike IgG responses compared to PEG-based formulations at early time points (Day 14 and Day 28). At later time points (Day 42), differences between groups were reduced but remained statistically significant (Figure 7E–H). The repeated immunizations with SARS-CoV-2 Spike S-2P mRNA-loaded LNP formulations elicited a robust humoral immune response in all treatment groups, as evidenced by the progressive increase in anti-SARS-CoV-2 spike IgG titers following each booster dose. Specifically, antibody levels measured 14 days after the second immunization were approximately 20-fold higher than those observed 14 days after the primary vaccination, indicating successful induction of immunological memory and effective recall responses. A further ∼5-fold increase in anti-SARS-CoV-2 spike IgG titers was observed following the third immunization, demonstrating continued responsiveness to repeated mRNA-LNP administration.^44^ Notably, the HEP-coated mRNA-LNPs consistently generated higher anti-SARS-CoV-2 spike IgG titers than the PEG-coated counterpart mRNA-LNP formulations.

The superior antibody production observed with HEP-coated mRNA-LNPs may result from reduced endolysosomal sequestration and enhanced delivery efficacy. By promoting more efficient endosomal escape and increasing the fraction of functional mRNA available for cytosolic protein translation, the HEP-based surface-engineering strategy likely increases antigen production in professional APCs, thereby improving antigen presentation, T-cell priming, and subsequent B-cell activation.^44^ The sustained superiority of HEP-coated mRNA-LNPs across multiple booster doses further suggests that improved intracellular trafficking can translate into meaningful enhancement of vaccine efficacy *in vivo*.

Although OPSS-HEP-modified LNPs elicited significantly higher anti-spike IgG responses than PEG-modified formulations, the magnitude of this improvement was relatively modest compared with the substantially larger enhancement in in vitro mRNA transfection. This difference is not unexpected, as vaccine-induced humoral immunity is determined by multiple biological processes beyond intracellular mRNA delivery, including antigen expression kinetics, antigen presentation, activation of innate and adaptive immune cells, and B-cell maturation.^44^ Immune responses are often nonlinear with respect to antigen expression, particularly once sufficient antigen levels have been achieved to effectively prime adaptive immunity.^45^ Consequently, improvements in cellular transfection efficiency are not necessarily reflected by proportional increases in antibody production. While the enhanced antigen-specific antibody responses observed with OPSS-HEP-modified LNPs support the improved immunogenicity of this platform, protective efficacy was not evaluated in the present study. Future studies employing appropriate viral challenge models, together with additional characterization of cellular immune responses and neutralizing antibody activity, will be important to determine whether the enhanced immunogenicity translates into improved protection against viral infection.^44^

Our findings support the potential of HEP polysaccharides as a biologically derived alternative to PEG, capable of enhancing both intracellular mRNA delivery and downstream vaccine-induced humoral immunity. As shown in Figure S28, anti-heparosan IgM and IgG were not detectable by ELISA under the conditions evaluated. Serum samples for IgM analysis were collected 5 days after the first administration of OPSS-HEP-modified mRNA-LNPs, whereas serum samples for IgG analysis were collected 14 days after the first administration. Notably, these assays were performed using a substantially larger serum input (5 μL/well) than that used for the anti-spike ELISA (0.002 μL/well). Since HEP polysaccharides are “self” molecules, this finding aligns with previous work on other HEP-modified macromolecules, which were shown not to elicit any adverse immune responses.^35,36^

Anti-PEG IgM and IgG responses were not measured in the present study. Therefore, the expression profiles observed following repeated administration cannot be attributed directly to the induction or avoidance of anti-PEG immunity. Moreover, the PEG-containing formulations examined here differ in PEG molecular weight and surface architecture, with DMG-PEG2000 and OPSS-PEG10k presenting distinct PEG configurations at the LNP surface.^4,6,7,46^ Because PEG chain length, surface presentation, and lipid anchoring may influence both anti-PEG antibody induction and assay recognition, a rigorous comparison would require validated formulation-specific assays together with appropriate positive controls.^42,47^ Likewise, although no anti-HEP antibody response was detected under the assay conditions employed, this should not be interpreted as definitive evidence that HEP is non-immunogenic. Future studies should quantify anti-PEG IgM and IgG responses and correlate these measurements with LNP pharmacological performance following repeated administration.

No abnormal body weight changes were observed in mice receiving repeated administrations of SARS-CoV-2 spike S-2P mRNA-LNPs throughout the 42-day vaccination study, suggesting that the formulations were well tolerated under the dosing regimen employed (Figure S29).

Histopathological evaluation of major organs, including liver, spleen, bone marrow, kidney, lung, injection site, and draining lymph nodes, revealed no evidence of tissue damage, inflammation, necrosis, or pathological abnormalities across all treatment groups (Figure 7I). Bone marrow sections exhibited preserved architecture, normal hematopoietic cellularity, and age-appropriate adipocyte distribution. Lung tissues maintained intact alveolar structure without signs of edema or inflammation, while kidney sections showed normal glomerular and tubular morphology. Liver histology demonstrated preserved lobular architecture with no indication of hepatocellular injury or steatosis. At the subcutaneous injection site, minimal interstitial expansion and sparse inflammatory cell infiltration were observed, consistent with a mild, expected local response to administration. No evidence of muscle damage or significant inflammation was detected. Similarly, spleen and draining lymph nodes exhibited well-preserved architecture with organized follicles and germinal centers, indicative of physiological immune activity rather than pathological remodeling. A summary of all histopathological observations is provided in Table S2.

To further evaluate the safety profile of the surface-modified mRNA-LNP formulations, we performed flow cytometry of splenic immune populations along with histopathological assessment following repeated subcutaneous administration in C57BL/6 mice. The mice received three doses of SARS-CoV-2 spike S-2P mRNA-LNPs at two-week intervals prior to analysis. As shown by the flow cytometry data in Figure S30, there were comparable distributions of major splenic immune cell populations, including plasma cells within B cells, CD4^+^ T cells, CD8^+^ T cells, and macrophages, across all LNP formulation groups (Figure S30D–I). No statistically significant differences were observed. Although booster immunizations increased anti-SARS-CoV-2 spike S-2P IgG levels, splenic immune cell composition remained unchanged. This observation suggests that antigen-specific humoral responses may arise from relatively low abundance activated cell subsets that do not substantially alter bulk immune cell distributions.^48,49^ Both histological analysis and flow cytometry indicated preserved immune homeostasis, with no evidence of excessive lymphoid proliferation or systemic immune perturbation. These results demonstrate that repeated administration of both PEG- and HEP-modified mRNA-LNPs elicits functional humoral immune responses while maintaining normal tissue architecture and immune cell composition, supporting a favorable safety profile. The findings further support HEP polysaccharide-based surface engineering of LNP formulations as a biocompatible PEG-free strategy for functional mRNA delivery.

We further noted that HEP polysaccharide-modified mRNA-LNPs achieved up to 10-fold higher *in vitro* transfection efficiency in professional APCs, including DC2.4 and RAW264.7 cells. However, the corresponding *in vivo* anti-SARS-CoV-2 Spike IgG titers showed only a moderate, though statistically significant, increase compared with PEG-modified LNP formulation counterparts. This difference may reflect the complex relationship between cellular tropism and the efficacy of vaccine-induced humoral immune responses. For example, recent studies suggest that direct mRNA translation within professional APCs is not the sole determinant of antigen-specific immune priming. Instead, even structural cells, such as skeletal muscle cells at the injection site, can serve as significant contributors to antigen production following intramuscular mRNA-LNP administration.^50^ Clinically used PEG-coated mRNA-LNP formulations can transfect myocytes efficiently, consistent with our *in vitro* data shown in Figure 2G for C2C12 skeletal muscle cells. This behavior can lead to sustained local production of soluble Spike antigen.^50^ The prolonged antigen availability may facilitate direct B-cell receptor recognition of conformational epitopes and support CD4+ T follicular helper (TFH)-mediated humoral responses, ultimately contributing to robust IgG production.^50^

In contrast, our HEP polysaccharide-coated mRNA-LNPs appear to shift cellular tropism away from myocytes and toward professional APCs for potential MHC presentation.^51^ As a potential result, a larger fraction of the expressed antigen may be directed into intracellular antigen-processing pathways rather than released into the extracellular environment as soluble protein.^50^ While this delivery strategy may improve antigen-presentation efficiency, it may reduce antigen availability to naïve B cells within draining lymph nodes. Together, these mechanisms may help explain why the increase in serum IgG titers was less dramatic than the substantial enhancement observed in our *in vitro* transfection experiments.^50,52,53^

Additionally, our longitudinal IVIS bioluminescence studies demonstrated that HEP polysaccharide-coated mRNA-LNPs maintained in vivo mRNA expression levels comparable to those of PEG-coated formulations following repeated administration. Because bioluminescence was assessed only at a single 6 h post-dose time point after each administration, the present data do not establish differences in the duration of mRNA expression or antigen exposure kinetics. Nevertheless, these findings indicate that replacement of PEG with HEP preserves repeated-dose in vivo delivery performance. Consistent with this observation, flow cytometry analysis revealed no evidence of excessive expansion of major immune cell populations, suggesting that HEP surface modification supports effective mRNA delivery without overt perturbation of immune homeostasis under the conditions examined.

Overall, the advantages of the HEP polysaccharide surface engineering strategy may not lie solely in maximizing peak serum IgG levels, but rather in achieving efficient and selective engagement of professional APCs while maintaining favorable tolerability and immunological balance. By matching or exceeding the *in vivo* performance of clinically relevant PEG-coated mRNA-LNP formulations without the potential limitations of anti-PEG immune responses and excessive structural cell transfection,^42^ our HEP polysaccharide surface-modification strategy represents a promising approach for repeat-administrable functional mRNA delivery systems.

### Proposed Mechanism of Reduced Endolysosomal Retention for Increased mRNA Delivery Efficacy

To integrate our experimental findings, we developed a proposed mechanistic model to illustrate how nanoparticle surface chemistry may regulate intracellular trafficking and ultimately affect functional mRNA delivery in professional APCs (Figure 8). Based on the quantitative live-cell imaging data presented in Figures 4–5, we observed the time-dependent colocalization of fluorescently labeled mRNA-LNP formulations with mCherry-Rab5-positive early endosomes, mCherry-Rab7-positive late endosomes, and mCherry-LAMP1-positive lysosomes over a 24 h period. While all formulations were internalized by cells, we observed substantial differences during endosomal maturation and intracellular processing.

**Figure 8:**
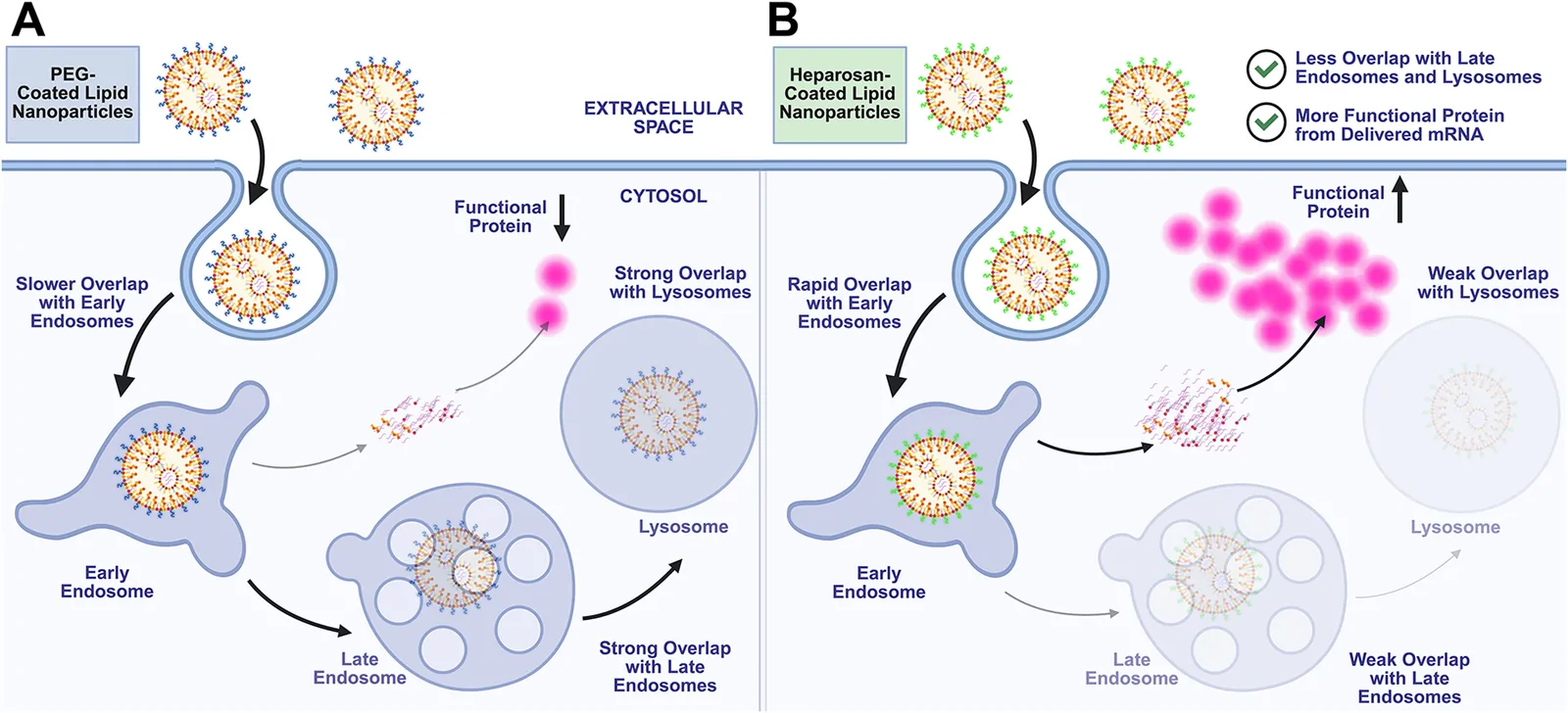
Schematic illustration of the proposed trafficking and delivery mechanisms in APCs. **(A)** PEG-coated mRNA-LNP formulations exhibit strong colocalization with late endosomes and lysosomes, limiting the efficiency of functional mRNA delivery and translation. (B) In contrast, heparosan polysaccharide-coated mRNA-LNPs exhibit relatively weaker colocalization with late endosomes and lysosomes, leading to enhanced delivery of functional mRNA and more efficient translation into functional protein. Created in BioRender. Wilhelm, S. (2026) https://BioRender.com/m65pb2j.

Our data showed that PEG-coated mRNA-LNP formulations were readily internalized by APCs but exhibited strong colocalization with mCherry-Rab7^+^ late endosomes and mCherry-LAMP1^+^ lysosomes. This observation suggests that a substantial fraction of internalized nanoparticles traffics to degradative endolysosomal compartments, where encapsulated mRNA is susceptible to enzymatic degradation before reaching the translational machinery (Figure 8A).^17,39^ Consequently, despite efficient cellular uptake, these formulations exhibit relatively low functional mRNA delivery efficiency, highlighting a critical disconnect between nanoparticle internalization and mRNA delivery efficacy.^35^

In contrast, the HEP polysaccharide-coated mRNA-LNPs demonstrated significantly reduced colocalization with both mCherry-Rab7-positive late endosomes and mCherry-LAMP1-positive lysosomes throughout the intracellular trafficking analysis (Figure 8B).^31^ LNPs coated with HEP polysaccharides appeared to redirect intracellular trafficking toward pathways that favor more efficient functional mRNA delivery for effective translation.^39^ Since PEG- and HEP-coated mRNA-LNPs exhibited similar cellular uptake, the enhanced transfection efficiency of HEP polysaccharide-coated mRNA-LNPs cannot be attributed to increased cellular internalization of these nanoparticles.

We propose that HEP polysaccharide-coated mRNA-LNPs facilitate more efficient endosomal escape during early endosomal maturation, potentially through enhanced membrane destabilization, altered vesicular membrane interactions, and increased frequency of transient budding-and-collapse escape events, thereby increasing the fraction of delivered mRNA that remains translationally competent.^1^ Collectively, our findings indicate that intracellular processing, rather than cellular uptake, is the principal determinant of mRNA delivery efficacy observed in this study.

Importantly, this mechanistic framework further helps explain the preferential increase in mRNA delivery efficacy in professional APCs. While these cells efficiently internalize nanoparticle formulations, a substantial portion may become trapped within the endolysosomal network and ultimately be eliminated via intracellular degradation pathways. The HEP polysaccharide surface engineering appears to overcome this limitation by facilitating early endosomal escape before lysosomal maturation. This shift effectively transforms professional APCs from nanoparticle scavengers into efficient mRNA-driven protein-producing cells.^53,54^

The altered intracellular trafficking observed for HEP-modified mRNA-LNPs provides a mechanistic framework that may contribute to their enhanced intracellular delivery. Specifically, the reduced localization of HEP-modified LNPs within Rab5-, Rab7-, and LAMP1-positive compartments is consistent with more efficient intracellular processing and increased opportunities for productive cytosolic mRNA delivery. Although repeated-dose bioluminescence imaging demonstrated comparable in vivo mRNA expression among the formulations at the measured post-dose time points, these findings indicate that replacing PEG with HEP preserves in vivo delivery performance while altering intracellular trafficking. Together, these results identify intracellular trafficking as an important contributor to mRNA delivery efficiency and support HEP polysaccharide-based surface engineering as a promising PEG-free strategy for the rational design of next-generation mRNA-LNPs and other nucleic acid delivery systems.

The present study extends our previous work on HEP surface-engineered mRNA-LNPs by focusing on the intracellular mechanisms that may underlie their enhanced biological performance. Whereas our previous study established the physicochemical properties, transfection efficiency, in vivo delivery, and biosafety of the HEP platform, the current work investigates the intracellular trafficking behavior of HEP-modified LNPs following cellular internalization. The reduced localization of HEP-modified LNPs within Rab5-, Rab7-, and LAMP1-positive compartments provides mechanistic evidence that HEP surface engineering alters intracellular processing, offering a biological explanation for the previously observed improved mRNA delivery. Although additional studies will be required to define the precise molecular mechanisms governing these trafficking differences, the present findings advance our understanding of how HEP surface chemistry influences the intracellular fate of mRNA-LNPs.

## Conclusions

In this study, we presented a modular strategy for the surface engineering of LNP formulations using HEP polysaccharides as PEG-free alternatives. Across multiple ionizable lipid systems, HEP polysaccharide-modified mRNA-LNP formulations maintained favorable physicochemical properties, high mRNA encapsulation efficiency, and minimal cytotoxicity, while significantly enhancing functional mRNA delivery in innate immune cell types. Importantly, this improvement was not associated with increased nanoparticle uptake by cells but instead correlated with altered intracellular trafficking and decreased endolysosomal retention. *In vivo*, HEP polysaccharide-modified mRNA-LNPs demonstrated comparable overall safety and efficacy to PEG-based systems while exhibiting more sustained expression dynamics under repeated dosing. Collectively, our findings establish HEP polysaccharide-based surface engineering as an effective strategy for improving intracellular mRNA delivery by modulating nanoparticle trafficking compared to commercially used PEGylated counterparts.

While the HEP-coated mRNA-LNPs in this study showed enhanced endosomal escape for better mRNA delivery to the cytosol, our previous studies with gold nanoparticles showed that HEP-coating can also enhance cell uptake by orders of magnitude.^9^ This behavior is primarily driven by the HEP polysaccharide surface density on gold nanoparticles.^10^ An important next step will be to determine whether these characteristics can be integrated into a single nanoparticle delivery platform that combines enhanced cellular internalization with optimized cytoplasmic transport, thereby maximizing intracellular bioavailability and therapeutic efficacy.

Several important avenues for future research remain. First, direct quantification of endosomal escape efficiency together with systematic investigation of endocytic pathways will further elucidate the intracellular mechanisms underlying HEP-mediated mRNA delivery.^37,38^ Because cellular uptake does not necessarily imply identical uptake mechanisms, complementary studies employing pharmacological or genetic perturbation of endocytic pathways will help distinguish the contributions of cellular internalization and intracellular trafficking.^1,44^

Second, comprehensive characterization of the protein corona formed on HEP- and PEG-modified LNPs under biologically relevant conditions will be important to determine how serum protein adsorption influences cellular uptake, intracellular trafficking, and transfection efficiency.^40^

Third, the distinct transfection profiles observed between APCs and C2C12 myoblasts warrant further investigation to determine whether cell-type-specific receptor expression, endocytic pathways, glycocalyx composition, or intracellular processing contribute to the biological effects of HEP surface modification. Fourth, comprehensive pharmacokinetic and biodistribution studies, together with post-dose expression kinetics, will further define the in vivo behavior of HEP-modified LNPs and determine whether HEP surface engineering prolongs functional mRNA expression following administration.^44,55^

Finally, dedicated immunogenicity studies, including quantitative assessment of anti-PEG responses, neutralizing antibody assays, and viral challenge models, will be valuable for determining whether the enhanced antigen-specific antibody responses observed in this study translate into improved protective immunity and long-term safety.^56^ In parallel, strategies to further enhance cellular uptake of HEP-modified mRNA-LNPs, including incorporation of targeting ligands or stimuli-responsive functionalities, may further improve delivery efficiency, safety, and therapeutic efficacy. Collectively, these studies will advance the rational design of next-generation PEG-free LNP systems and further define the role of polysaccharide-based surface engineering in mRNA delivery.

## Methods and Materials

### General Materials

M5-0.8 x 1 m threaded rod (Fabory, M20230.050.1000, United States, Norman, Oklahoma); M5 Nut (Ace Hardware, 5165774, United States, Moore, Oklahoma); 5x5 mm shaft coupler (Befenybay, BE-032–7-fba, United States, Norman, Oklahoma); Ender3 Kit (Amazon, United States, Norman, Oklahoma); BD PrecisionGlide needles (Fisher Scientific, BD305122, United States, Houston, Texas); Amicon Ultra-4 centrifugal filter unit, 100-kDa MWCO (Millipore Sigma, UFC 10024, United States, Missouri); Amicon Ultra-0.5 centrifugal filter unit, 100-kDa MWCO (Millipore Sigma, UFC510096, United States, Missouri); 1.6 mm OD T-mixer (Idex Health and Science, P-712, United States, Carlsbad, California); PEEK Cross mixer, 1.6 mm OD tubing (Restek, 27722, United States, Bellefonte, Pennsylvania); 25G needles (Fisher Scientific, United States, Houston, Texas) 305124; 10 mL Luerlock syringe (Fisher Scientific, B302995, United States, Houston, Texas); BD Slip Tip 1 mL syringe (Fisher Scientific, BD309659, United States, Houston, Texas).

### Lipid Nanoparticle Materials

(6Z,9Z,28Z,31Z)-Heptatriaconta-6,9,28,31-tetraen-19-yl 4-(dimethylamino)­butanoate (DLINMC3-DMA) (Medkoo Biosciences, 555308, United States, Morrisville, North Carolina); heptadecan-9-yl 8-((2-hydroxyethyl)­(6-oxo-6-(undecyloxy)­hexyl)­amino)­octanoate (SM-102) (Avanti Lipids, 792885, United States, Alabaster, Alabama); 6-((2-hexyldecanoyl)­oxy)-N-(6-((2-hexyldecanoyl)­oxy)­hexyl)-N-(4-hydroxybutyl)­hexan-1-aminium (ALC-0315) (Avanti Lipids, 890900); 1,2 distearyol-*sn*-glycero-3-phosphocholine (DSPC) (Avanti Polar Lipids, 850365); Cholesterol (Avanti Polar Lipids, LM4100); 16:0 Ptd Thioethanol (Avanti Lipids, 880151); mPEG-OPSS MW 10-kDa (Laysan Bio, 162–32, United States, Arab, Alabama); 170-kDa HEP,7-kDa OPSS-HEP, and 13-kDa OPSS-HEP were synthesized and characterized in-house, and the general conjugation processes followed our published methods.^15,16^

A 3.6-kDa OPSS-HEP was prepared in two steps: (i) an unsulfated HEP 18-mer (gift of Glycan Therapeutics, Raleigh, North Carolina, USA) was synthesized by repetitive step-wise synthesis from a p-nitrophenyl-beta-glucuronide primer using PmHS2 enzyme, UDP-GlcA and UDP-GlcNAc sugar donors, then converted to a HEP-NH_2_ derivative as described;^57^ (ii) this reagent was then coupled with SPDP (Fisher Scientific #21857) as above to make the thiol-reactive HEP-coating reagent in-house. The 13-kDa maleimide-HEP was also synthesized and characterized in-house; it was prepared in a similar fashion to the 13-kDa OSSP-derivative except that the heterobifunctional reagent 6-Maleimidocaproic acid N-succinimidyl ester (Fisher Scientific H65996-MD) was substituted for the SPDP reagent when coupling to the HEP-NH_2_.

Citrate Buffer (pH 4.0, 100 mM) (Fisher Scientific, Q2444, United States, Houston, Texas); RiboGreen Quantification Kit (Thermofisher, R11490, United States, Houston, Texas); Spectra/Por 2 Trial Kit, 12–14 kDa (Repligen, 132678T, United States, Boston, Massachusetts). 5 EZ Cap Firefly Luciferase mRNA (APExBIO, R1018, United States, Houston, Texas); EZ Cap EGFP mRNA (5-moUTP) (APExBIO, R1016, United States, Houston, Texas). Luciferase Assay System (Promega Products, E1501, United States, Madison, Wisconsin). D-Luciferin, Potassium Salt (Gold Biotechnology (U.S. Registration No 3,257,927)).

### Cell Culture Materials

RAW 264.7 mouse macrophages (ATCC, TIB-71, Manassas, VA, USA); DC2.4 murine dendritic cells (MilliporeSigma, SCC142, Burlington, MA, USA); C2C12 murine myoblasts (ATCC, CRL-1772, Manassas, VA, USA); Dulbecco’s modified Eagle’s medium (DMEM), high glucose, pyruvate (Thermo Fisher, 11995065, United States, Houston, Texas); Roswell Park Memorial Institute 1640 medium (RPMI 1640) with l-glutamine (Thermo Fisher Scientific, 11875093, Waltham, MA, USA); fetal bovine serum (Thermo Fisher, 16000044, United States, Houston, Texas); penicillin– streptomycin (Thermo Fisher, 15–140-122, United States, Houston, Texas); paraformaldehyde solution (PFA), 4% in PBS (Thermo Fisher, J19943K2, United States, Houston, Texas); wheat germ agglutinin (WGA), CF633 conjugate (Biotium, 29024, United States, Fremont, California); NucBlue Fixed Cell ReadyProbes reagent (DAPI) (Thermo Fisher, R37606, United States, Houston, Texas); 18 mm round coverslips #1 (VWR, 16004–300, United States, Missouri City, Texas); #1.5H glass bottom dishes (Fisher Scientific, 50–305-807, United States, Houston, Texas); 12-well cell culture plate (VWR, 10062–894, United States, Missouri City, Texas); μ-Slide 8-well high glass bottom (ibidi, cat# 980807, United States, Fitchburg, Wisconsin); Pierce 96-well polystyrene plates, white opaque (Thermo Fisher, 15042, United States, Houston, Texas).

### Instruments

Dynamic Light Scattering (Malvern Zetasizer Nano ZS, Enigma Business Park, United Kingdom); Field-flow fractionation (FFF) coupled with multi-angle light scattering (MALS; Wyatt Technology, United States, Santa Barbara, California); Centrifuge (Thermo Scientific, Heraeus Multifuge X3R, United States, Houston, Texas); Plate Reader (Aglient, BioTek Synergy Neo2, United States, Santa Clara, California); Andor BC43 Benchtop spinning disk confocal laser scanning microscope (Andor, Belfast, UK); Cytek NorthernLights flow cytometry (Cytek Biosciences), Data were analyzed using FlowJo v10.7.1.; PerkinElmer IVIS Spectrum CT (PerkinElmer, Inc., United States, Waltham, Massachusetts); ChemiDoc MP imager (Bio-Rad; Hercules, CA).

## Methods

### Characterization of OPSS-HEP by PAGE and SAMSA Fluorescein Labeling

13-kDa HEP-NH_2_ and OPSS-HEP were characterized by 10% TBE polyacrylamide gel electrophoresis (PAGE). For fluorescent labeling, polymers were reacted with activated SAMSA Fluorescein (Thermo Fisher Scientific, A685) at a 2:1 molar ratio of SAMSA Fluorescein to polymer in 50 mM HEPES buffer (pH 7.2) at a polymer concentration of 2 mg mL^–1^ for 1 h at room temperature. SAMSA Fluorescein was activated to form a fluorescein with a free sulfhydryl group (Fluor-SH) before use by deprotection in 0.1 M NaOH for 15 min at room temperature and subsequently neutralized with HCl. A total of 2 μg of polymer was loaded per lane, and electrophoresis was performed on a 10% TBE PAGE gel at 250 V for 20 min. Gels were first imaged for Fluorescein fluorescence and then stained with 0.05% Alcian Blue to visualize polysaccharides.

### LNPs Preparation and Surface Modification

DMG-PEG2000 coated mRNA-LNPs were prepared by microfluidic mixing.^14,26^ Lipids were dissolved in ethanol at a total lipid concentration of 3.2 mM. Three ionizable lipid formulations were prepared with the following molar compositions: DLin-MC3-DMA:DSPC:cholesterol:DMG-PEG2000, SM-102:DSPC:cholesterol:DMG-PEG2000, or ALC-0315:DSPC:cholesterol:DMG-PEG2000 at 50:10:38.5:1.5 mol %. The aqueous phase consisted of mRNA dissolved in 10 mM citrate buffer at pH 4.0–4.5. Different N/P ratios were achieved by varying the amount of mRNA in the aqueous phase while maintaining the total lipid concentration constant. The aqueous and ethanolic phases were mixed at an aqueous-to-organic flow-rate ratio of 7:1 to promote nanoparticle self-assembly and control particle size.

Following synthesis, LNP suspensions were dialyzed against 1×PBS to remove residual ethanol and equilibrate the formulations to physiological pH. Briefly, LNP suspensions were transferred into dialysis tubing with a molecular weight cutoff of 12–14 kDa and dialyzed against at least a 100-fold excess volume of 1×PBS at pH 7.4 at 4 °C with gentle stirring. The dialysis buffer was replaced 2–3 times over 12–24 h. Final LNP formulations were collected and stored at 4 °C until further characterization or use.

Similarly, OPSS-HEP– or OPSS-PEG–modified mRNA-loaded LNPs were prepared by microfluidic mixing following a previously reported method with minor modifications. Briefly, lipids were dissolved in ethanol at a total lipid concentration of 3.2 mM. For thiol-containing formulations, the thiol-functionalized lipid was first dissolved in 100% ethanol and incubated in a 37 °C water bath, followed by brief sonication as needed to ensure complete dissolution prior to mixing with the remaining lipid components. Three ionizable lipid formulations were prepared with the following molar compositions: DLin-MC3-DMA:DSPC:cholesterol:thiol-lipid, SM-102:DSPC:cholesterol:thiol-lipid, or ALC-0315:DSPC:cholesterol:thiol-lipid at 50:10:38.5:1.5 mol %. The aqueous phase consisted of mRNA dissolved in citrate buffer at pH 4.0–4.5. Different N/P ratios were achieved by varying the amount of mRNA in the aqueous phase while keeping the total lipid concentration constant. The aqueous and ethanolic phases were mixed at an aqueous-to-organic flow-rate ratio of 7:1 to facilitate nanoparticle self-assembly and control particle size.

Immediately following LNP formation, 250 μL of 10 mM HEPES buffer (pH 12) was gently added to 4 mL of LNP suspension to adjust the final formulation pH to approximately 7.0. For post-synthetic surface modification, 1 mL aliquots of the neutralized LNP suspension were transferred into microcentrifuge tubes containing OPSS-HEP or OPSS-PEG. The OPSS-functionalized polymers were added at a 1:1 molar ratio relative to the thiol-lipid, based on previous optimization studies. The mixtures were gently vortexed for 10 min to facilitate thiol–disulfide exchange between the thiol-functionalized lipids and OPSS-HEP or OPSS-PEG. This post-conjugation strategy enabled surface functionalization while preserving the core LNP composition and formulation process.

Following surface modification, LNP formulations were dialyzed against 1×PBS to remove residual ethanol and equilibrate the particles to physiological pH conditions. Briefly, LNP suspensions were transferred into dialysis tubing with a molecular weight cutoff (MWCO) of 12–14 kDa and dialyzed against at least a 100-fold excess volume of 1×PBS (pH 7.4) at 4 °C with gentle stirring. The dialysis buffer was replaced 2–3 times over a period of 12–24 h. Final LNP formulations were collected and stored at 4 °C until further characterization or experimental use.

### Physicochemical Characterization

The hydrodynamic diameter (HDD), polydispersity index (PDI), and zeta potential were measured with a Malvern ZetaSizer Nano ZS instrument. The nanoparticle number concentration and size distributions were determined by FFF-MALS (Wyatt). The mRNA encapsulation efficiency was quantified using the commonly used RiboGreen assay (Thermo Fisher, R11490, United States, Houston, Texas).

### *In Vitro* Transfection

RAW 264.7 murine macrophages, DC2.4 dendritic cells, and C2C12 myoblasts were seeded at a density of 1×10^4^ cells per well in white-walled, clear-bottom 96-well plates containing 100 μL of complete culture medium and allowed to adhere overnight at 37 °C in a humidified 5% CO_2_ incubator. The medium consisted of DMEM supplemented with 10% FBS and 1% (v/v) penicillin-streptomycin. The cells were allowed to adhere overnight in a humidified 37 °C incubator with 5% CO_2_.

The following day, firefly luciferase mRNA-loaded LNPs were diluted in fresh complete medium to achieve the desired dose in 100 μL per well. The spent medium was gently removed from each well, and cells were treated with either the diluted mRNA-LNP formulations or complete medium alone (negative control). The treated plate was returned to the incubator and incubated for 24 h.

To evaluate transfection efficiency, a luciferase assay was performed. The luciferase reagent and 1× cell lysis buffer were prepared following the manufacturer’s protocol. The 96-well plate was transferred to a biosafety cabinet. Media were aspirated from each well and cells were washed once with 1×PBS. Then, 20 μL of 1×lysis buffer was added to each well, followed by 100 μL of the luciferase assay reagent (Promega, Cat. No.E1500). The plate was protected from light and immediately analyzed using a BioTek Synergy Neo2Multi-Mode Plate Reader to measure bioluminescence. All luminescence values were exported and analyzed using GraphPad Prism. Transfection efficiency was quantified based on normalized luminescence intensity.

### Cytotoxicity

RAW 264.7 murine macrophages, DC2.4 dendritic cells, and C2C12 myoblasts were seeded at a density of 1×10^4^ cells per well in white-walled, clear-bottom 96-well plates containing 100 μL of complete culture medium and allowed to adhere overnight at 37 °C in a humidified 5% CO_2_ incubator. Complete medium consisted of DMEM supplemented with 10% fetal bovine serum (FBS) and 1% (v/v) penicillin–streptomycin. The following day, culture medium was removed and replaced with 100 μL of fresh complete medium containing mRNA-loaded LNP formulations at the indicated doses. Cells treated with complete medium alone served as negative controls. After 24 h incubation, cells were washed three times with PBS, and cell viability was evaluated using the XTT assay (2,3-bis­(2-methoxy-4-nitro-5-sulfophenyl)-2H-tetrazolium-5-carboxanilide; Sigma-Aldrich, Cat. No. 11465015001) according to the manufacturer’s instructions. Absorbance was measured at 450 nm with a reference wavelength of 650 nm using a microplate reader. Cell viability was expressed as the percentage of viable cells relative to untreated control cells.

### ELISA Assays

#### In Vitro SARS-CoV-2 Spike Protein Expression

RAW 264.7 cells were seeded in 24-well plates and treated with SARS-CoV-2 spike S-2P mRNA-loaded LNPs formulated at an N/P ratio of 8. After 24 h of incubation, both cell culture supernatants and cell lysates were collected for analysis. Spike protein levels were quantified using a commercial SARS-CoV-2 Spike Detection ELISA Kit (Sino Biological, Cat. #KIT40591) according to the manufacturer’s instructions. Absorbance was measured at 450 nm with 630 nm as the reference wavelength, and spike protein concentrations were calculated based on a linear standard curve. The total spike protein expression for each sample was determined by summing the concentrations measured in the corresponding culture supernatant and cell lysate.

### Serum Anti-Spike IgG Analysis

Twenty-two-week-old C57BL/6J mice (Jax #000664) were purchased from Jackson Laboratories. The investigators followed the “Guide for the Care and Use of Laboratory Animals” by the Committee on Care of Laboratory Animal Resources Commission on Life Sciences, National Research Council. The animal facilities at the University of Oklahoma are fully accredited by the American Association for Accreditation of Laboratory Animal Care (AAALAC). All studies were conducted in accordance with protocols approved by the University of Oklahoma IACUC (protocol #2024–0301).

Female C57BL/6 mice (n = 5 per group) were immunized by subcutaneous injection with SARS-CoV-2 spike (S-2P) mRNA-LNPs (2 μg mRNA in 100 μL of 1× PBS) on Days 0, 14, and 28. Serum anti-SARS-CoV-2 spike IgG titers were evaluated in mice immunized with SARS-CoV-2 spike S-2P mRNA-LNP formulations. Blood samples were collected by submandibular bleeding on Days 0, 14, 28, and 42 prior to vaccine administration. Whole blood samples were allowed to clot at room temperature and subsequently centrifuged to isolate serum. Anti-spike S1 IgG titers were quantified (0.002 μL sera/well) using a Mouse Anti-SARS-CoV-2 Antibody IgG Titer Serologic Assay Kit (Spike S1) (ACROBiosystems, Cat. RAS-T045-96tests) according to the manufacturer’s protocol. Absorbance was measured at 450 nm with 630 nm as the reference wavelength, and antibody titers were calculated based on a linear standard curve.

### Serum Anti-HEP IgM and IgG Analysis

Potential anti-HEP antibody responses were evaluated using custom-prepared ELISA plates and anti-mouse IgG probes. Briefly, HEP-amine (23-kDa; 2 mg/mL in 50 mM HEPES, pH 7.2; 100 ml/well; synthesized by the same method as the 13-kDa HEP-NH_2_ reagent used to make the LNP-coating reagent) or 50 mM Tris, pH 7.2, was used to coat amine-binding, maleic anhydride-activated 96-well plates (Cat. #15100, Thermo Scientific Pierce). After an overnight incubation at room temperature, the amine solution was aspirated, wells washed with water thrice, and then flooded with 50 mM Tris, pH 7.2, followed by 0.1% BSA in 50 mM Tris, pH 7.2. After incubating for 1.5 h, the BSA blocking solution was aspirated, and the wells were rinsed with water once and stored at 4 °C in a sealed bag until use in the ELISA. The HEP coating on the wells was verified by radiolabeling with recombinant HEP synthase and UDP-[^3^H]­GlcNAc.^15^

The HEP-coated wells were used to detect HEP-specific antibodies, while Tris-coated wells served as background and negative controls. Serum samples were collected from mice treated with OPSS-HEP-coated SARS-CoV-2 spike S-2P mRNA-LNPs on Days 0, 5, and 14 following subcutaneous administration on Day 0. Mouse serum samples (5 μL sera/well) were added to the coated wells and incubated for 1 h to allow antibody binding. After extensive washing, bound antibodies were detected using HRP-conjugated goat anti-mouse IgG and IgM secondary antibodies (MilliporeSigma, Cat. AP130P). The HRP signal was developed using a chromogenic substrate solution, and absorbance was measured at 450 nm with 630 nm as the reference wavelength. Antibody levels were determined following subtraction of background absorbance values obtained from Tris-coated control wells.

### Confocal Laser Scanning Microscopy (CLSM)

Cellular uptake, intracellular distribution, and EGFP expression were analyzed by CLSM using an Andor BC43 spinning-disk confocal microscope (Oxford Instruments). RAW 264.7 macrophages or DC2.4 dendritic cells were seeded at a density of 1×10^4^ cells per well in μ-Slide 8-well high glass-bottom chambers (Ibidi, Cat. 80807) and incubated overnight prior to treatment.

For nanoparticle uptake studies, LNPs were fluorescently labeled with Vybrant DiO Cell-Labeling Solution (Thermo Fisher Scientific, Cat. V22886, Eugene, OR, USA). DiO, a lipophilic fluorescent membrane dye, was dissolved in 100% ethanol at a concentration of 0.044 mg/mL and incorporated into the lipid phase prior to microfluidic mixing during LNP formulation. Following nanoparticle preparation, formulations were dialyzed overnight against 1×PBS to remove unincorporated free dye.

Cells were treated with DiO-labeled EGFP mRNA-LNP formulations and incubated for 24 h prior to imaging. Before microscopy, nuclei and plasma membranes were stained using appropriate fluorescent dyes according to the manufacturers’ protocols. Confocal images were acquired under identical imaging settings for all experimental groups to allow direct comparison of cellular uptake and EGFP expression levels.

### Flow Cytometry

RAW 264.7 cells were seeded in 12-well plates at a density of 2×10^5^ cells per well in complete medium and incubated overnight at 37 °C with 5% CO_2_ to allow cell adhesion. The following day, cells were treated with LNPs encapsulating either 0.5 or 1 μg of EGFP mRNA and surface-modified with OPSS-HEP, DMG-PEG2000, or OPSS-PEG. As controls, a negative control group (untreated; no mRNA or transfection reagent) and a positive control group (transfected with 2 μg EGFP mRNA using Lipofectamine according to the manufacturer’s instructions) were included.

After 24 h, cells were washed once with 1× PBS, detached using a sterile cell scraper, and immediately transferred to ice to halt further endocytosis and exocytosis. All subsequent steps were performed on ice. Cells were pelleted by centrifugation (500 xg, 3 min), and the supernatant was removed. The pellets were washed once with ice-cold 1×PBS and centrifuged. The resulting cell pellets were resuspended in 300 μL PBS, and 5 μL of ViaDye Red Fixable Viability Dye (Cytek, SKU R7-60008), pre-diluted 1:500, was added to each sample. Staining was performed on ice for 15 min to assess membrane integrity and viability. Following staining, cells were washed once more with PBS by centrifugation and resuspended in 300 μL PBS for flow cytometry. Controls included unstained cells, single-stained cells (ViaDye Red only), and cells treated with LNPs but not stained, to facilitate spectral unmixing and compensation. Samples were analyzed using a Cytek Northern Lights spectral flow cytometer (Cytek Biosciences). A minimum of 20,000 events per sample was collected per sample. Data acquisition was followed by analysis using FlowJo software (v10.7.1).

### Live-Cell Trafficking Analysis

RAW 264.7 macrophages or DC2.4 dendritic cells were seeded at a density of (1x10^4^) cells per well in μ-Slide 8-well high glass-bottom chambers (ibidi, Cat. 80807) and allowed to attach overnight. Plasmids encoding mCherry-labeled Rab5, Rab7, or LAMP1 were purchased from GenScript (Piscataway, NJ, USA)­and used to label early endosomes, late endosomes, and lysosomes, respectively. Cells were transfected with 100 ng/well of mCherry-Rab5, mCherry-Rab7, or mCherry-LAMP1 plasmid using Lipofectamine 3000 (Thermo Fisher Scientific) according to the manufacturer’s instructions and incubated for 48 h prior to the live-cell imaging experiments.

For live-cell trafficking studies, EGFP mRNA-loaded LNPs were fluorescently labeled with 1,1′-dioctadecyl-3,3,3′,3′-tetramethylindodicarbocyanine perchlorate (DiD; Thermo Fisher Scientific, Vybrant DiD Cell-Labeling Solution, Cat. V22887) 0.05 mol %, a lipophilic far-red fluorescent membrane dye incorporated into the lipid phase during LNP formulation to enable visualization of nanoparticle localization and intracellular trafficking. Prior to nanoparticle treatment, transfected cells were washed with PBS and incubated with DiD-labeled EGFP mRNA-LNP formulations. Each well was treated with mRNA-LNPs containing 1 μg of EGFP mRNA.

Live-cell confocal imaging was performed at designated time points corresponding to the dynamics of individual intracellular compartments. Rab5 trafficking was imaged at 5, 15, 30, and 45 min post-treatment; Rab7 trafficking at 0.75, 1.5, and 3 h; and LAMP1 trafficking at 1, 2, 4, 6, and 24 h. Before imaging, culture medium was replaced with Live Cell Imaging Solution (Thermo Fisher Scientific, Cat. A14291DJ) to maintain physiological conditions during microscopy. Time-dependent EGFP expression was simultaneously monitored as an indicator of successful cytosolic mRNA delivery and translation. Confocal images were acquired using an Andor BC43 spinning-disk confocal microscope (Oxford Instruments). Colocalization analysis between DiD-labeled LNPs and mCherry-tagged intracellular compartment markers was performed using ImageJ software and Manders’ colocalization coefficients were calculated to quantify nanoparticle trafficking behavior over time. Manders’ colocalization coefficients were calculated to quantify the intracellular trafficking behavior of mRNA-LNPs over time. For each condition, regions of interest (ROIs) containing 10–15 cells expressing mCherry-Rab5, mCherry-Rab7, or mCherry-LAMP1 were analyzed per image, and a total of three independent images were quantified for each group.

### *In Vivo* Bioluminescence Imaging

Eight-week-old C57BL/6J mice (Jax #000664) were purchased from Jackson Laboratories. To evaluate the *in vivo* transfection efficiency of surface-modified LNPs, C57BL/6 mice (8 weeks old, n=3 per group) were subcutaneously injected with 2.0 μg of luciferase mRNA-LNPs coated with DMG-PEG200, OPSS-PEG, or OPSS-HEP surface coatings in 100 μL of 1× PBS at day 0, 3, 10, 66, 69, 76, delivered into the flank region under sterile conditions. Bioluminescence imaging was performed at 6 h post-injection using the IVIS SpectrumCT Imaging System (PerkinElmer). At each time point, mice were anesthetized with 2.5% isoflurane and injected subcutaneously with 150 mg/kg D-luciferin potassium salt (GoldBio, Cat# LUCK).

After a 10 min incubation to stabilize the bioluminescent signal, whole-body luminescence signals were captured using the IVIS system with auto-exposure settings, automatically adjusting exposure time, binning, and f/stop to optimize signal quality and avoid saturation. Bioluminescence intensity was visualized using a pseudo-color scale and quantified as total flux (photons/sec) using Living Image Software (PerkinElmer). Regions of interest (ROIs) were drawn around the injection site to quantify signal intensity. All image acquisition and analysis were performed using Living Image Software.

### Mice Spleen Flow Cytometry Analysis

Twenty 22-week-old female C57BL/6J mice (C57BL/6J mouse; Jackson Laboratory, Cat. #000664) were used in this study. Mice were randomly divided into 4 groups (n=5 per group), including one PBS control group and three experimental groups receiving SARS-CoV-2 spike S-2P mRNA-LNP formulations surface-modified with DMG-PEG2000, OPSS-PEG, or OPSS-HEP. Mice received subcutaneous injections on Days 0, 14, and 28, with each injection containing 2 μg of mRNA formulated in the respective LNPs. Blood samples were collected by submandibular bleeding on Days 0, 14, 28, and 42 prior to each injection or terminal analysis.

On Day 42, following blood collection, mice were euthanized by CO_2_ asphyxiation and spleens were harvested for flow cytometric analysis. Spleens were mechanically dissociated into single-cell suspensions and processed using standard flow cytometry procedures. Cells were washed using wash buffer. Wash buffer consisted of RPMI 1640 medium supplemented with 5% newborn calf serum (NCS), and FACS buffer consisted of 1×PBS supplemented with 2% NCS.

Cell viability staining was performed using ViaDye Red viability dye (Cytek Biosciences, Cat. R0-00008) diluted 1:10,000 in PBS. Fc receptors were blocked using anti-mouse CD16/32 Fc Block (Tonbo Biosciences, Cat. 70–0161-U500, clone 2.4G2) diluted 1:200 in FACS buffer.

Cells were subsequently stained with fluorophore-conjugated antibodies against CD45 (BV421, clone 30-F11, BioLegend), CD4 (FITC, clone RM4-5, Tonbo Biosciences), F4/80 (PE-Dazzle 594, clone BM8, BioLegend), CD3e (PerCP-Cy5.5, clone 145–2C11, Tonbo Biosciences), CD19 (PE-Cy7, clone 1D3/eBio1D3, Invitrogen), CD138 (PE-Fire 810, clone 281–2, BioLegend), CD8a (APC, clone 53–6.7, Tonbo Biosciences), and CD11b (redFluor 710, clone M1/70, Tonbo Biosciences). All antibodies were diluted 1:100 in FACS buffer prior to staining.

Following staining and washing, samples were analyzed using a spectral flow cytometer (Cytek Biosciences), and data were processed using FlowJo software. Live single cells were gated prior to immune cell population analysis.

### Histopathological Analysis

To evaluate the in vivo biocompatibility of the formulations, mice received three subcutaneous administrations of DMG-PEG2000-, OPSS-PEG-, or OPSS-HEP-coated SARS-CoV-2 spike S-2P mRNA-LNPs at two-week intervals. Each injection contained 2 μg of mRNA formulated in the respective LNPs. Fourteen days after the third immunization, blood samples were collected by submandibular bleeding prior to euthanasia. Mice were subsequently euthanized by CO_2_ asphyxiation, and major organs and tissues were harvested for histopathological evaluation.

The major organs, including the liver, spleen, kidney, lung, injection site, tibia bone, and draining lymph nodes, were collected at the designated study end points. Lungs were perfused with 10 mLs perfusion buffer (1×PBS without Mg^2+^ /Ca^2+^ supplemented with 1 mM EDTA) prior to harvest. Tissues were immediately fixed in 10% neutral-buffered formalin (VN-FF0266-1L, VWR, VION) for a minimum of 72 h, dehydrated, and embedded in paraffin. Bone specimens were subjected to decalcification by immersion in EDTA/Sucrose solution (Decalcifying Solution, 1048C, Newcomer Supply) for 14 days prior to embedding.

Paraffin blocks were sectioned at 4–5 μm using a rotary microtome and mounted on glass slides. For routine histopathology, sections were stained with H&E according to standard protocols. Slides were examined under light microscopy (Leica DM5000B or equivalent) by a board-certified pathologist blinded to treatment groups. Histological evaluation focused on necrosis, inflammation, fibrosis, immune cell infiltration, and other pathological alterations. Positive and negative controls were included in all staining procedures. Histopathological findings were recorded semi-quantitatively as incidence and severity scores following established criteria. Data were summarized as incidence (a/b), where “a” represents the number of affected animals and “b” the total number examined. Representative images were acquired using a digital pathology scanner (Aperio AT2, Leica Biosystems) at 20× magnification.

### Statistical Analysis

Data are presented as mean ± SD unless otherwise stated. Statistical significance was determined using GraphPad Prism, with p<0.05 considered significant.

## Supplementary Material



## Notes

It includes additional characterization data, including the chemical structure and characterization of OPSS-HEP, supplementary nanoparticle characterization, additional in vitro mRNA expression data, intracellular trafficking images and analyses, immune response measurements, histopathological evaluation, and spleen immune cell profiling (PDF)

## References

[1] Hou X., Zaks T., Langer R., Dong Y. (2021). Lipid Nanoparticles
for MRNA Delivery. Nat. Rev. Mater..

[2] Donahue N. D., Acar H., Wilhelm S. (2019). Concepts of
Nanoparticle Cellular
Uptake, Intracellular Trafficking, and Kinetics in Nanomedicine. Adv. Drug Delivery Rev..

[3] Donahue N. D., Francek E. R., Kiyotake E., Thomas E. E., Yang W., Wang L., Detamore M. S., Wilhelm S. (2020). Assessing
Nanoparticle
Colloidal Stability with Single-Particle Inductively Coupled Plasma
Mass Spectrometry (SP-ICP-MS). Anal. Bioanal.
Chem..

[4] Yang Q., Lai S. K. (2015). Anti-PEG Immunity: Emergence, Characteristics,
and
Unaddressed Questions. Wiley Interdiscip. Rev.:
Nanomed. Nanobiotechnol..

[5] Grenier P., Viana I. M. D. O., Lima E. M., Bertrand N. (2018). Anti-Polyethylene Glycol
Antibodies Alter the Protein Corona Deposited on Nanoparticles and
the Physiological Pathways Regulating Their Fate in Vivo. J. Controlled Release.

[6] Senti M. E., de Jongh C. A., Dijkxhoorn K., Verhoef J. J. F., Szebeni J., Storm G., Hack C. E., Schiffelers R. M., Fens M. H., Boross P. (2022). Anti-PEG Antibodies
Compromise the
Integrity of PEGylated Lipid-Based Nanoparticles via Complement. J. Controlled Release.

[7] Deuker M. F. S., Mailänder V., Morsbach S., Landfester K. (2023). Anti-PEG Antibodies
Enriched in the Protein Corona of PEGylated Nanocarriers Impact the
Cell Uptake. Nanoscale Horiz..

[8] Yang W., Wang L., Mettenbrink E. M., DeAngelis P. L., Wilhelm S. (2021). Nanoparticle Toxicology. Annu.
Rev. Pharmacol. Toxicol..

[9] Yang W., Wang L., Fang M., Sheth V., Zhang Y., Holden A. M., Donahue N. D., Green D. E., Frickenstein A. N., Mettenbrink E. M., Schwemley T. A., Francek E. R., Haddad M., Hossen M. N., Mukherjee S., Wu S., DeAngelis P. L., Wilhelm S. (2022). Nanoparticle Surface Engineering with Heparosan Polysaccharide
Reduces Serum Protein Adsorption and Enhances Cellular Uptake. Nano Lett..

[10] Yang W., Frickenstein A. N., Sheth V., Holden A., Mettenbrink E. M., Wang L., Woodward A. A., Joo B. S., Butterfield S. K., Donahue N. D., Green D. E., Thomas A. G., Harcourt T., Young H., Tang M., Malik Z. A., Harrison R. G., Mukherjee P., Deangelis P. L., Wilhelm S. (2022). Controlling Nanoparticle
Uptake in Innate Immune Cells with Heparosan Polysaccharides. Nano Lett..

[11] Sheth V., Chen X., Mettenbrink E. M., Yang W., Jones M. A., M’saad O., Thomas A. G., Newport R. S., Francek E., Wang L., Frickenstein A. N., Donahue N. D., Holden A., Mjema N. F., Green D. E., DeAngelis P. L., Bewersdorf J., Wilhelm S. (2023). Quantifying Intracellular Nanoparticle
Distributions with Three-Dimensional Super-Resolution Microscopy. ACS Nano.

[12] Frickenstein A. N., Mukherjee S., Harcourt T., He Y., Sheth V., Wang L., Malik Z., Wilhelm S. (2023). Quantification of Monodisperse
and Biocompatible Gold Nanoparticles by Single-Particle ICP-MS. Anal. Bioanal. Chem..

[13] Frickenstein A. N., Means N., He Y., Whitehead L., Harcourt T., Malik Z., Sheth V., Longacre L., Taffe H., Wang L., McSpadden I., Baroody C., Yang W., Zhao Y. D., Wilhelm S. (2024). The Predictive
Synthesis of Monodisperse and Biocompatible Gold Nanoparticles. ACS Appl. Nano Mater..

[14] He Y., Wang L., Velayutham R., Valerio T. I., Ricketts S. D., Sun Y., Annan A. C., Bowman J., Tran T., Mohammadnejad M., Liu K., Chen W. R., Green D. E., Fung K. M., DeAngelis P. L., Wilhelm S. (2026). Lipid Nanoparticle Surface Engineering with Heparosan
Polysaccharides for Safe and Effective MRNA Delivery In Vitro and
In Vivo. ACS Appl. Mater. Interfaces.

[15] Lane R. S., Haller F. M., Chavaroche A. A. E., Almond A., Deangelis P. L. (2017). Heparosan-Coated
Liposomes for Drug Delivery. Glycobiology.

[16] Deangelis P. L. (2015). Heparosan,
a Promising “naturally Good” Polymeric Conjugating Vehicle
for Delivery of Injectable Therapeutics. Expert
Opin. Drug Delivery.

[17] Gilleron J., Querbes W., Zeigerer A., Borodovsky A., Marsico G., Schubert U., Manygoats K., Seifert S., Andree C., Stöter M., Epstein-Barash H., Zhang L., Koteliansky V., Fitzgerald K., Fava E., Bickle M., Kalaidzidis Y., Akinc A., Maier M., Zerial M. (2013). Image-Based Analysis
of Lipid Nanoparticle-Mediated SiRNA Delivery, Intracellular Trafficking
and Endosomal Escape. Nat. Biotechnol..

[18] Ndeupen S., Qin Z., Jacobsen S., Bouteau A., Estanbouli H., Igyártó B. Z. (2021). The MRNA-LNP
Platform’s Lipid
Nanoparticle Component Used in Preclinical Vaccine Studies Is Highly
Inflammatory. iScience.

[19] He Y., Wang Y., Wang L., Jiang W., Wilhelm S. (2024). Understanding
Nanoparticle-Liver Interactions in Nanomedicine. Expert Opin. Drug Delivery.

[20] Wang L., Quine S., Frickenstein A. N., Lee M., Yang W., Sheth V. M., Bourlon M. D., He Y., Lyu S., Garcia-Contreras L. (2024). Exploring and Analyzing
the Systemic Delivery
Barriers for Nanoparticles. Adv. Funct. Mater..

[21] Mohammadnejad M., Haddad M., Frickenstein A. N., Dambold A., Sheth V., Alexandre J., Means N., Bowman J., Belligund K., Moss H., Park J., He Y., Wilhelm S. (2026). Label-Free
and High-Throughput Quantification of Nanoparticle–Cell Interactions
at the Single-Cell Level with Flow Cytometry. Anal. Chem..

[22] Chatterjee S., Kon E., Sharma P., Peer D. (2024). Endosomal Escape: A Bottleneck for
LNP-Mediated Therapeutics. Proc. Natl. Acad.
Sci. U. S. A..

[23] Adams D., Gonzalez-Duarte A., O’riordan W. D., Yang C.-C., Ueda M., Kristen A. V., Tournev I., Schmidt H. H., Coelho T., Berk J. L., Lin K.-P., Vita G., Attarian S., Planté-Bordeneuve V., Mezei M. M., Campistol J. M., Buades J., Brannagan T. H., Kim B. J., Oh J., Parman Y., Sekijima Y., Hawkins P. N., Solomon S. D., Polydefkis M., Dyck P. J., Gandhi P. J., Goyal S., Chen J., Strahs A. L., Nochur S. V., Sweetser M. T., Garg P. P., Vaishnaw A. K., Gollob J. A., Suhr O. B. (2018). Patisiran,
an RNAi Therapeutic, for Hereditary Transthyretin Amyloidosis. N. Engl. J. Med..

[24] Corbett K. S., Flynn B., Foulds K. E., Francica J. R., Boyoglu-Barnum S., Werner A. P., Flach B., O’connell S., Bock K. W., Minai M., Nagata B. M., Andersen H., Martinez D. R., Noe A. T., Douek N., Donaldson M. M., Nji N. N., Alvarado G. S., Edwards D. K., Flebbe D. R., Lamb E., Doria-Rose N. A., Lin B. C., Louder M. K., O’dell S., Schmidt S. D., Phung E., Chang L. A., Yap C., Todd J.-P. M., Pessaint L., Van Ry A., Browne S., Greenhouse J., Putman-Taylor T., Strasbaugh A., Campbell T.-A., Cook A., Dodson A., Steingrebe K., Shi W., Zhang Y., Abiona O. M., Wang L., Pegu A., Yang E. S., Leung K., Zhou T., Teng I.-T., Widge A., Gordon I., Novik L., Gillespie R. A., Loomis R. J., Moliva J. I., Stewart-Jones G., Himansu S., Kong W.-P., Nason M. C., Morabito K. M., Ruckwardt T. J., Ledgerwood J. E., Gaudinski M. R., Kwong P. D., Mascola J. R., Carfi A., Lewis M. G., Baric R. S., McDermott A., Moore I. N., Sullivan N. J., Roederer M., Seder R. A., Graham B. S. (2020). Evaluation of the
MRNA-1273 Vaccine against SARS-CoV-2 in Nonhuman Primates. N. Engl. J. Med..

[25] Polack F. P., Thomas S. J., Kitchin N., Absalon J., Gurtman A., Lockhart S., Perez J. L., Marc G. P., Moreira E. D., Zerbini C., Bailey R., Swanson K. A., Roychoudhury S., Koury K., Li P., Kalina W. V., Cooper D., Frenck R. W., Hammitt L. L., Türeci Ö., Nell H., Schaefer A., Ünal S., Tresnan D. B., Mather S., Dormitzer P. R., Şahin U., Jansen K. U., Gruber W. C. (2020). Safety and Efficacy
of the BNT162b2MRNA Covid-19 Vaccine. N. Engl.
J. Med..

[26] Young H., He Y., Joo B., Ferguson S., Demko A., Butterfield S. K., Lowe J., Mjema N. F., Sheth V., Whitehead L., Ruiz-Echevarria M. J., Wilhelm S. (2024). Toward the Scalable, Rapid, Reproducible,
and Cost-Effective Synthesis of Personalized Nanomedicines at the
Point of Care. Nano Lett..

[27] Luozhong S., Liu P., Li R., Yuan Z., Debley E., Chen Y., Hu Y., Cao Z., Cui M., McIlhenny K., McCurdy C., Bhashyam D., Wilkens S., Zhang P., Kwan A., Grossman M., Lai R., Ma Y., Lipkin S., Jiang S. (2025). Poly (Carboxybetaine)
Lipids Enhance
MRNA Therapeutics Efficacy and Reduce Their Immunogenicity. Nat. Mater..

[28] Lee J. C., Donahue N. D., Mao A. S., Karim A., Komarneni M., Thomas E. E., Francek E. R., Yang W., Wilhelm S. (2020). Exploring
Maleimide-Based Nanoparticle Surface Engineering to Control Cellular
Interactions. ACS Appl. Nano Mater..

[29] Dutta K., Das R., Medeiros J., Thayumanavan S. (2021). Disulfide Bridging Strategies in
Viral and Nonviral Platforms for Nucleic Acid Delivery. Biochemistry.

[30] Haghighi E., Abolmaali S. S., Dehshahri A., Shaegh S. A. M., Azarpira N., Tamaddon A. M. (2024). Navigating the Intricate
In-Vivo Journey of Lipid Nanoparticles
Tailored for the Targeted Delivery of RNA Therapeutics: A Quality-by-Design
Approach. J. Nanobiotechnology.

[31] Huotari J., Helenius A. (2011). Endosome Maturation. EMBO J..

[32] Rink J., Ghigo E., Kalaidzidis Y., Zerial M. (2005). Rab Conversion as a
Mechanism of Progression from Early to Late Endosomes. Cell.

[33] Wilhelm S., Tavares A. J., Dai Q., Ohta S., Audet J., Dvorak H. F., Chan W. C. W. (2016). Analysis of Nanoparticle
Delivery
to Tumours. Nat. Rev. Mater..

[34] Manders E. M. M., Verbeek F. J., Aten J. A. (1993). Measurement
of Co-localization of
Objects in Dual-colour Confocal Images. J. Microsc..

[35] Wittrup A., Ai A., Liu X., Hamar P., Trifonova R., Charisse K., Manoharan M., Kirchhausen T., Lieberman J. (2015). Visualizing Lipid-Formulated SiRNA Release from Endosomes
and Target Gene Knockdown. Nat. Biotechnol..

[36] Patel S., Kim J., Herrera M., Mukherjee A., Kabanov A. V., Sahay G. (2019). Brief Update
on Endocytosis of Nanomedicines. Adv. Drug Delivery
Rev..

[37] Pei D. (2025). Endosomal
Escape of Lipid Nanoparticles: A Perspective on the Literature Data. ACS Nano.

[38] Herrera M., Kim J., Eygeris Y., Jozic A., Sahay G. (2021). Illuminating Endosomal
Escape of Polymorphic Lipid Nanoparticles That Boost MRNA Delivery. Biomater. Sci..

[39] Johansson J. M., Du Rietz H., Hedlund H., Eriksson H. C., Blenke E. O., Pote A., Harun S., Nordenfelt P., Lindfors L., Wittrup A. (2025). Cellular and Biophysical
Barriers
to Lipid Nanoparticle Mediated Delivery of RNA to the Cytosol. Nat. Commun..

[40] Voke E., Arral M. L., Squire H. J., Lin T. J., Zheng L., Coreas R., Lui A., Iavarone A. T., Pinals R. L., Whitehead K. A. (2025). Protein Corona Formed
on Lipid Nanoparticles
Compromises Delivery Efficiency of MRNA Cargo. Nat. Commun..

[41] Simberg D., Moghimi S. M. (2024). Anti-Poly­(Ethylene Glycol) (PEG) Antibodies: From Where
Are We Coming and Where Are We Going. J. Nanotheranostics.

[42] Fu S., Zhu X., Huang F., Chen X. (2025). Anti-PEG Antibodies and Their Biological
Impact on PEGylated Drugs: Challenges and Strategies for Optimization. Pharmaceutics.

[43] Pardi N., Tuyishime S., Muramatsu H., Kariko K., Mui B. L., Tam Y. K., Madden T. D., Hope M. J., Weissman D. (2015). Expression
Kinetics of Nucleoside-Modified MRNA Delivered in Lipid Nanoparticles
to Mice by Various Routes. J. Controlled Release.

[44] Pardi N., Hogan M. J., Porter F. W., Weissman D. (2018). MRNA Vaccines-a New
Era in Vaccinology. Nat. Rev. Drug Discovery.

[45] Aung A., Irvine D. J. (2024). Modulating Antigen
Availability in Lymphoid Organs
to Shape the Humoral Immune Response to Vaccines. J. Immunol..

[46] Ju Y., Lee W. S., Pilkington E. H., Kelly H. G., Li S., Selva K. J., Wragg K. M., Subbarao K., Nguyen T. H. O., Rowntree L. C., Allen L. F., Bond K., Williamson D. A., Truong N. P., Plebanski M., Kedzierska K., Mahanty S., Chung A. W., Caruso F., Wheatley A. K., Juno J. A., Kent S. J. (2022). Anti-PEG Antibodies Boosted in Humans
by SARS-CoV-2 Lipid Nanoparticle MRNA Vaccine. ACS Nano.

[47] Kozma G. T., Shimizu T., Ishida T., Szebeni J. (2020). Anti-PEG Antibodies:
Properties, Formation, Testing and Role in Adverse Immune Reactions
to PEGylated Nano-Biopharmaceuticals. Adv. Drug
Delivery Rev..

[48] Goel R. R., Apostolidis S. A., Painter M. M., Mathew D., Pattekar A., Kuthuru O., Gouma S., Hicks P., Meng W., Rosenfeld A. M. (2021). Distinct Antibody and
Memory B Cell Responses
in SARS-CoV-2 Naïve and Recovered Individuals after MRNA Vaccination. Sci. Immunol..

[49] Lederer K., Bettini E., Parvathaneni K., Painter M. M., Agarwal D., Lundgreen K. A., Weirick M., Muralidharan K., Castaño D., Goel R. R. (2022). Germinal Center Responses
to SARS-CoV-2 MRNA Vaccines in Healthy and Immunocompromised Individuals. Cell.

[50] Marks A., Siu S., Bianchini F., Wang C., Lakshmi A., Phelan M., Zhu A., Moon C., Morla-Folch J., Teunissen A. J. P. (2026). mRNA Vaccine Immunity Is Enhanced by Hepatocyte Detargeting
and Not Dependent on Dendritic Cell Expression. Nat. Biotechnol..

[51] Liang F., Lindgren G., Lin A., Thompson E. A., Ols S., Röhss J., John S., Hassett K., Yuzhakov O., Bahl K., Brito L. A., Salter H., Ciaramella G., Loré K. (2017). Efficient Targeting and Activation of Antigen-Presenting
Cells In Vivo after Modified MRNA Vaccine Administration in Rhesus
Macaques. Mol. Ther..

[52] Kim S., Jeon J. H., Kim M., Lee Y., Hwang Y. H., Park M., Li C. H., Lee T., Lee J. A., Kim Y. M. (2024). Innate Immune Responses
against MRNA Vaccine Promote
Cellular Immunity through IFN-β at the Injection Site. Nat. Commun..

[53] Turner J. S., O’halloran J.
A., Kalaidina E., Kim W., Schmitz A. J., Zhou J. Q., Lei T., Thapa M., Chen R. E., Case J. B., Amanat F., Rauseo A. M., Haile A., Xie X., Klebert M. K., Suessen T., Middleton W. D., Shi P. Y., Krammer F., Teefey S. A., Diamond M. S., Presti R. M., Ellebedy A. H. (2021). SARS-CoV-2 MRNA
Vaccines Induce Persistent
Human Germinal Centre Responses. Nature.

[54] Gustafson H. H., Holt-Casper D., Grainger D. W., Ghandehari H. (2015). Nanoparticle
Uptake: The Phagocyte Problem. Nano Today.

[55] Sandelius Å., Naseer H., Lindqvist J., Wilson A., Henderson N. (2024). Biodistribution
of Lipid Nanoparticle, EGFP MRNA and Translated Protein Following
Subcutaneous Administration in Mouse. Bioanalysis.

[56] Pardi N., Hogan M. J., Naradikian M. S., Parkhouse K., Cain D. W., Jones L., Moody M. A., Verkerke H. P., Myles A., Willis E., LaBranche C. C., Montefiori D. C., Lobby J. L., Saunders K. O., Liao H. X., Korber B. T., Sutherland L. L., Scearce R. M., Hraber P. T., Tombácz I., Muramatsu H., Ni H., Balikov D. A., Li C., Mui B. L., Tam Y. K., Krammer F., Karikó K., Polacino P., Eisenlohr L. C., Madden T. D., Hope M. J., Lewis M. G., Lee K. K., Hu S. L., Hensley S. E., Cancro M. P., Haynes B. F., Weissman D. (2018). Nucleoside-Modified
MRNA Vaccines Induce Potent T Follicular Helper and Germinal Center
B Cell Responses. J. Exp. Med..

[57] Arnold K., Xu Y., Sparkenbaugh E. M., Li M., Han X., Zhang X., Xia K., Piegore M., Zhang F., Zhang X. (2020). Design of Anti-Inflammatory Heparan Sulfate to Protect
against Acetaminophen-Induced Acute Liver Failure. Sci. Transl. Med..

