# Supplementary material for: Lipid Nanoparticle Surface Engineering with Heparosan Polysaccharides Enhances mRNA Efficacy via Reduced Endosomal Retention
Source: ACS Nano. 2026 Sep 24;20(39):26664–86. doi: 10.1021/acsnano.6c11865 (PMC13648266; doi:10.1021/acsnano.6c11865)
Supplement: Supplementary file 1 [file nn6c11865_si_001.pdf]

Supporting Information

Lipid Nanoparticle Surface Engineering with Heparosan Polysaccharides Enhances mRNA Efficacy via Reduced Endosomal Retention

Yuxin He[1], Lin Wang[1], Abigael P. Williams[1], Trisha I. Valerio[1], Yuanhong Sun[1], James Bowman[1], Samantha D. Ricketts[2], Savannah Hammond[1], Aidan Sison[1], Jezan Alexandre[1], Zachary Niastor[1], Wei R. Chen[1], Dixy E. Green[3], Kar-Ming Fung[2,5], Paul L. DeAngelis[3], Stefan Wilhelm[1,4,5,6,7,*]

[1] Stephenson School of Biomedical Engineering, University of Oklahoma, Norman, Oklahoma, 73019, USA

[2] Department of Pathology, University of Oklahoma Health Campus, Oklahoma City, Oklahoma, 73104, USA

[3] Department of Biochemistry and Physiology, University of Oklahoma Health Campus, Oklahoma City, Oklahoma, 73104, USA

[4] Institute for Biomedical Engineering, Science, and Technology (IBEST), University of Oklahoma, Norman, Oklahoma, 73019, USA

[5] Stephenson Cancer Center, University of Oklahoma, Oklahoma City, Oklahoma, 73104, USA

[6] Harold Hamm Diabetes Center, University of Oklahoma, Oklahoma City, Oklahoma, 73104, USA

[7] Materials Science and Engineering Program, University of Oklahoma, Norman, Oklahoma, 73019, USA

*Corresponding author:
Stefan Wilhelm, Ph.D.
Email: stefan.wilhelm@ou.edu
ORCID: 0000-0003-2167-6221

Table of Contents

Supporting Information Figures and Tables..........5

Table S1: Lipid nanoparticle (LNP) compositions of formulations developed in this study and clinically approved LNPs..........6

Figure S1. Chemical structures and characterization of OPSS-modified heparosan (OPSS-HEP). 7

Figure S2. Screening of ionizable lipids and surface modifications for mRNA-loaded lipid nanoparticles (LNPs). 8

Figure S3: Physicochemical characterization of DMG-PEG2000-, OPSS-PEG-, and OPSS-HEP-modified Vybrant™ DiO-labeled luciferase mRNA-LNPs at an N/P ratio of 8. 9

Figure S4: Physicochemical characterization of DMG-PEG2000 (#2) -, OPSS-PEG (#5) -, and OPSS-HEP (#8) - modified EGFP mRNA-LNPs at an N/P ratio of 8. 10

Figure S5. Surface modification enhances mRNA delivery efficiency without altering cellular uptake of EGFP mRNA-loaded LNPs. 11

Figure S6. Cellular transfection efficiency of surface-modified EGFP mRNA-loaded lipid nanoparticles in DC2.4 dendritic cells. 12

Figure S7. Flow cytometry gating strategy for analyzing EGFP-positive RAW 264.7 cells. 13

Figure S8: Physicochemical characterization of DMG-PEG2000-, OPSS-PEG-, and OPSS-HEP-modified with lipophilic carbocyanine fluorescent dye DiD-labeled EGFP mRNA-LNPs used for live-cell imaging at an N/P ratio of 8. 14

Figure S9: Live-cell imaging and intracellular localization of DiD-labeled EGFP mRNA-LNPs with different surface modifications in DC2.4 cells during early endosomal transport (5 minutes and 15 minutes). 16

Figure S10: Live-cell imaging and intracellular localization of DiD-labeled EGFP mRNA-LNPs with different surface modifications in DC2.4 cells during early endosomal transport (30 minutes and 45 minutes). 18

Figure S11: Live-cell imaging and intracellular localization of DiD-labeled EGFP mRNA-LNPs with different surface modifications in DC2.4 cells during late endosomal transport (45 minutes and 90 minutes). 20

Figure S12: Live-cell imaging and intracellular localization of DiD-labeled EGFP mRNA-LNPs with different surface modifications in DC2.4 cells during late endosomal transport (180 minutes). 21

Figure S13: Live-cell imaging and intracellular localization of DiD-labeled EGFP mRNA-LNPs with different surface modifications in DC2.4 cells during lysosome transport (1 hour and 2 hours). 22

Figure S14: Live-cell imaging and intracellular localization of DiD-labeled EGFP mRNA-LNPs with different surface modifications in DC2.4 cells during lysosome transport (4 hours and 6 hours). 24

Figure S15: Live-cell imaging and intracellular localization of DiD-labeled EGFP mRNA-LNPs with different surface modifications in DC2.4 cells during lysosome transport (24 hours). 26

Figure S16: Time-lapse live-cell imaging of intracellular trafficking and localization of OPSS-HEP-modified mRNA-LNPs in DC2.4 dendritic cells. 28

Figure S17: Live-cell imaging and intracellular localization of DiD-labeled EGFP mRNA-LNPs with different surface modifications in RAW 264.7 cells during early endosomal transport (5 minutes and 15 minutes). 30

Figure S18: Live-cell imaging and intracellular localization of DiD-labeled EGFP mRNA-LNPs with different surface modifications in RAW 264.7 cells during early endosomal transport (30 minutes and 45 minutes). 32

Figure S19: Live-cell imaging and intracellular localization of DiD-labeled EGFP mRNA-LNPs with different surface modifications in RAW 264.7 cells during late endosomal transport (45 minutes and 90 minutes). 34

Figure S20: Live-cell imaging and intracellular localization of DiD-labeled EGFP mRNA-LNPs with different surface modifications in RAW 264.7 cells during late endosomal transport (180 minutes). 35

Figure S21: Live-cell imaging and intracellular localization of DiD-labeled EGFP mRNA-LNPs with different surface modifications in RAW 264.7 cells during lysosome transport (1 hour and 2 hours). 37

Figure S22: Live-cell imaging and intracellular localization of DiD-labeled EGFP mRNA-LNPs with different surface modifications in RAW 264.7 cells during lysosome transport (4 hours and 6 hours). 38

Figure S23: Live-cell imaging and intracellular localization of DiD-labeled EGFP mRNA-LNPs with different surface modifications in RAW 264.7 cells during lysosome transport. 40

Figure S24: Physicochemical characterization of DMG-PEG2000-, OPSS-PEG-, and OPSS-HEP-modified firefly luciferase mRNA-LNPs used for IVIS studies at an N/P ratio of 8. .41

Figure S25: Body weight monitoring of mice following repeated administration of firefly luciferase mRNA-LNPs modified by DMG-PEG2000-, OPSS-PEG-, or OPSS-HEP. 42

Figure S26: Physicochemical characterization and *in vitro* cytotoxicity assessment of SARS-CoV-2 mRNA-LNPs at an N/P ratio of 8. 43

Figure S27: Physicochemical characterization of SARS-CoV-2 spike S-2P mRNA mRNA-LNPs used for three-dose *in vivo* studies at an N/P ratio of 8. 44

Figure S28: Quantification of potential anti-HEP antibodies in serum from SARS-CoV-2 spike S-2P mRNA OPSS-HEP (#8) mRNA-LNPs-treated mice. 45

Figure S29: Body weight monitoring of mice following repeated administration of SARS-CoV-2 spike S-2P mRNA-LNPs. 46

Figure S30: Spleen immune profiling of C57BL/6 mice following repeated subcutaneous administration of SARS-CoV-2 spike S-2P mRNA-LNPs with different surface modifications. 47

Table S2: Summary of Histopathological Findings. 48

Summary of histopathological findings in SARS-CoV-2 mRNA-LNP–treated mice. 48

Supporting Information Figures and Tables

Table S1: Lipid nanoparticle (LNP) compositions of formulations developed in this study and clinically approved LNPs.

LNP Formulation Name	Cargo	Ionizable Lipid (mol%)	Phospholipid (mol%)	Cholesterol (mol%)	Other Lipid* (mol%)	Molar Ratio (Ionizable Lipid : Phospholipid : Cholesterol : Other Lipid*)
OPSS-HEP-LNP (This Study)	mRNA	DLIN-MC3-DMA (50.0)	DSPC (10.0)	Cholesterol (38.5)	Thiol-lipid (1.5) + OPSS-HEP	50:10:38.5:1.5
OPSS-HEP-LNP (This Study)	mRNA	SM-102 (50.0)	DSPC (10.0)	Cholesterol (38.5)	Thiol-lipid (1.5) + OPSS-HEP	50:10:38.5:1.5
OPSS-HEP-LNP (This Study)	mRNA	ALC-0315 (50.0)	DSPC (10.0)	Cholesterol (38.5)	Thiol-lipid (1.5) + OPSS-HEP	50:10:38.5:1.5
OPSS-PEG-LNP (This Study)	mRNA	DLIN-MC3-DMA (50.0)	DSPC (10.0)	Cholesterol (38.5)	Thiol-lipid (1.5) + OPSS-PEG	50:10:38.5:1.5
OPSS-PEG-LNP (This Study)	mRNA	SM-102 (50.0)	DSPC (10.0)	Cholesterol (38.5)	Thiol-lipid (1.5) + OPSS-PEG	50:10:38.5:1.5
OPSS-PEG-LNP (This Study)	mRNA	ALC-0315 (50.0)	DSPC (10.0)	Cholesterol (38.5)	Thiol-lipid (1.5) + OPSS-PEG	50:10:38.5:1.5
DMG-PEG-LNP (This Study	mRNA	DLIN-MC3-DMA (50.0)	DSPC (10.0)	Cholesterol (38.5)	DMG-PEG2000 (1.5)	50:10:38.5:1.5
DMG-PEG-LNP (This Study)	mRNA	SM-102 (50.0)	DSPC (10.0)	Cholesterol (38.5)	DMG-PEG2000 (1.5)	50:10:38.5:1.5
DMG-PEG-LNP (This Study)	mRNA	ALC-0315 (50.0)	DSPC (10.0)	Cholesterol (38.5)	DMG-PEG2000 (1.5)	50:10:38.5:1.5
Onpattro® (Patisiran)	siRNA	DLin-MC3-DMA (50.0)	DSPC (10.0)	Cholesterol (38.5)	DMG-PEG2000 (1.5)	50:10:38.5:1.5
Comirnaty® (Pfizer-BioNTech, BNT162b2)	mRNA	ALC-0315 (46.3)	DSPC (9.4)	Cholesterol (42.7)	ALC-0159 (1.6)	46.3:9.4:42.7:1.6
Spikevax® (Moderna, mRNA-1273)	mRNA	SM-102 (50.0)	DSPC (10.0)	Cholesterol (38.5)	DMG-PEG2000 (1.5)	50:10:38.5:1.5

OPSS-HEP: Orthopyridyl disulfide conjugated heparosan-13,000 Da
OPSS-PEG: Orthopyridyl disulfide conjugated poly(ethylene glycol)-10,000 Da
DLIN-MC3-DMA: (6Z,9Z,28Z,31Z)-heptatriaconta-6,9,28,31-tetraen-19-yl 4-(dimethylamino)butanoate
DSPC: 1,2-distearoyl-sn-glycero-3-phosphocholine
SM-102: heptadecan-9-yl 8-((2-hydroxyethyl)(6-oxo-6-(undecyloxy)hexyl)amino)octanoate
ALC-0315: 6-((2-hexyldecanoyl)oxy)-N-(6-((2-hexyldecanoyl)oxy)hexyl)-N-(4-hydroxybutyl)hexan-1-aminium
*Other Lipid: 16:0 PTE (Thiol-Lipid), DMG-PEG, or other PEG-Lipid components
16:0 PTE: 1,2-Dipalmitoyl-sn-Glycero-3-Phosphothioethanol
DMG-PEG2000: 1,2-dimyristoyl-rac-glycero-3-methoxypolyethylene glycol-2,000 Da
ALC-0159: Methoxypolyethyleneglycoloxy(2,000 Da)-N,N-ditetradecylacetamide

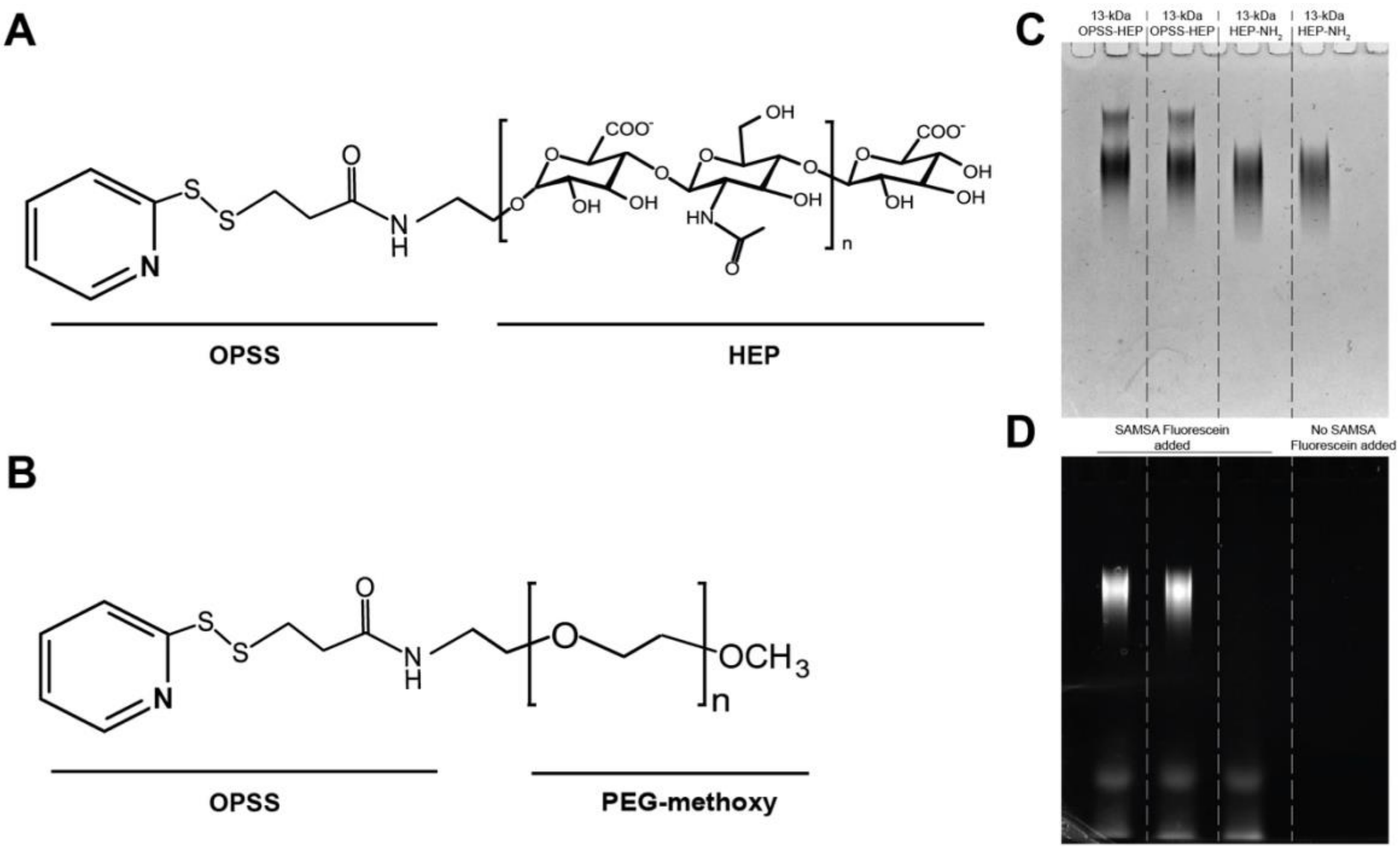

Figure S1. Chemical structures and characterization of OPSS-modified heparosan (OPSS-HEP). (A) Chemical structures of OPSS-HEP. **(B)** Chemical structure of OPSS-PEG. **(C)** Alcian Blue-stained 10% polyacrylamide gel electrophoresis (PAGE) analysis of 13-kDa OPSS-HEP and HEP-NH_2 (amine-functionalized 13-kDa HEP). HEP-NH_2 is the precursor of the OPSS-HEP compound. The upper Alcian bands in the OPSS-HEP lanes correspond to the dimeric HEP-S-S-HEP species that forms when a small amount of the OPSS-moiety degrades, unveiling a sulfhydryl group that can react with another intact OPSS-HEP; typically, the dimer is ~10% of the preparation, thus an extra 10% is added to compensate for the LNP-coating. **(D)** Fluorescence PAGE analysis following reaction with activated SAMSA Fluorescein. OPSS-HEP samples exhibited fluorescent bands after reaction with activated SAMSA Fluorescein (Fluor-SH), whereas HEP-NH_2 showed no detectable fluorescence under the same reaction conditions, confirming successful OPSS functionalization. The HEP-dimer does not appreciably react with the Fluor-SH on short time scales; thus, no glow is observed.

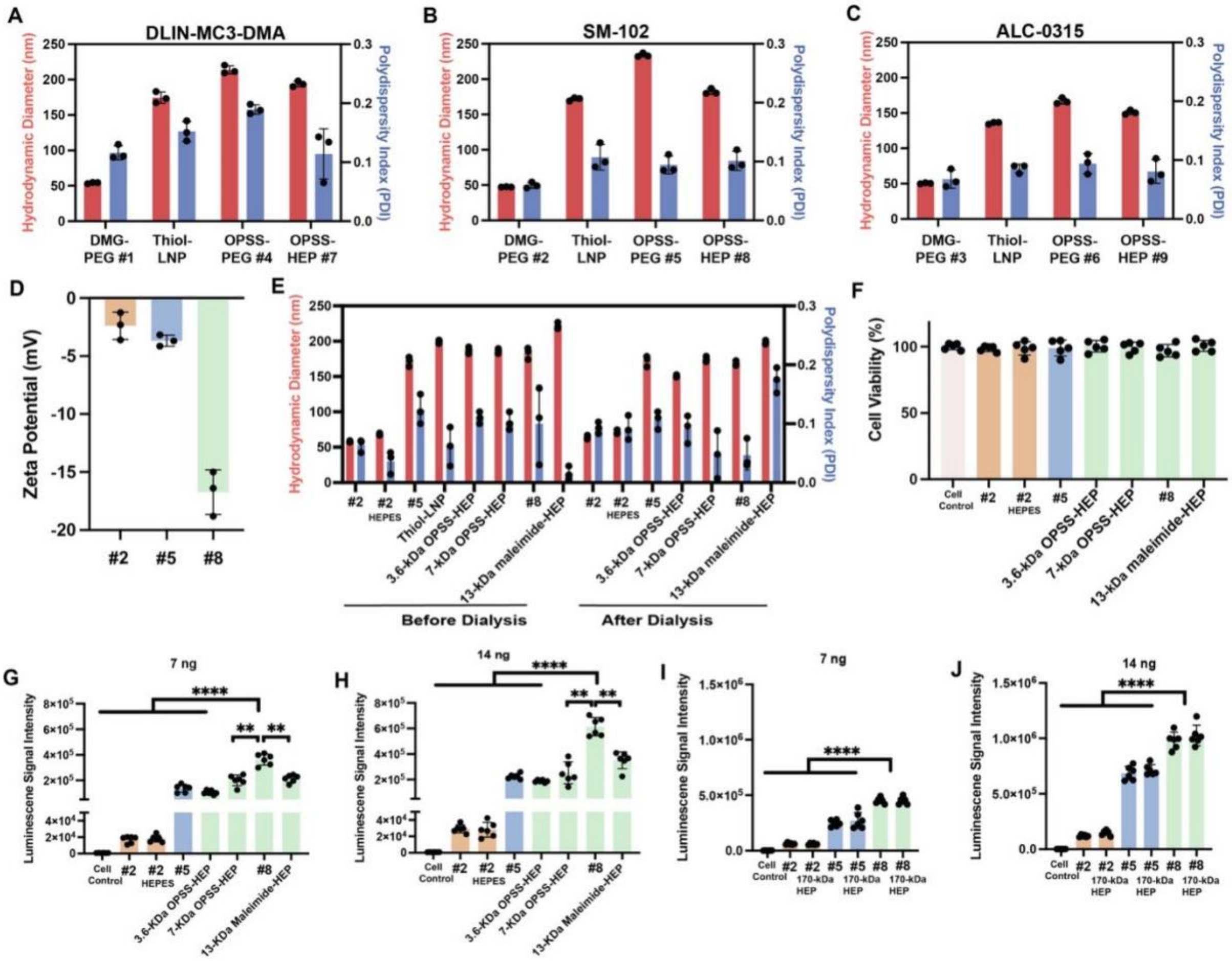

Figure S2. Screening of ionizable lipids and surface modifications for mRNA-loaded lipid nanoparticles (LNPs). (A–C) Hydrodynamic diameter (HDD, red) and polydispersity index (PDI, blue) of mRNA-LNPs formulated with (A) DLIN-MC3-DMA, (B) SM-102, or (C) ALC-0315 prior to dialysis, measured by dynamic light scattering (DLS). Data are presented as mean ± SD (n=3). **(D)** Zeta potentials of DMG-PEG2000-, OPSS-PEG-, and OPSS-HEP-modified mRNA-LNPs made with SM-102 ionizable lipid were measured by electrophoretic mobility in 10 mM HEPES buffer (pH 7.4). Data are presented as mean ± SD (n=3). **(E)** HDD and PDI of mRNA-LNPs with different surface modifications, including DMG-PEG2000, OPSS-PEG (10 kDa), OPSS-HEP (3.6, 7, or 13 kDa), and maleimide-HEP (13 kDa), measured by DLS. Data are presented as mean ± SD (n=3). **(F)** Cell viability of RAW 264.7 macrophages following 24-h incubation with the indicated LNP formulations, determined by XTT assay. Data are presented as mean ± SD (n=5). No significant differences in cell viability were observed (one-way ANOVA, p>0.05). **(G, H)** Luciferase bioluminescence in RAW 264.7 macrophages 24 h after treatment with mRNA-LNPs containing 7 ng or 14 ng firefly luciferase mRNA (N/P ratio = 100). Data are presented as mean ± SD (n=6). **(I, J)** Luciferase bioluminescence in RAW 264.7 macrophages treated with mRNA-LNPs containing 7 ng or 14 ng firefly luciferase mRNA (N/P ratio = 100) following addition of free 170-kDa HEP. Data are presented as mean ± SD (n=6). Addition of free 170-kDa HEP did not significantly alter transfection efficiency. Statistical significance was determined by one-way ANOVA with multiple comparisons; ***p<0.001, ****p<0.0001.

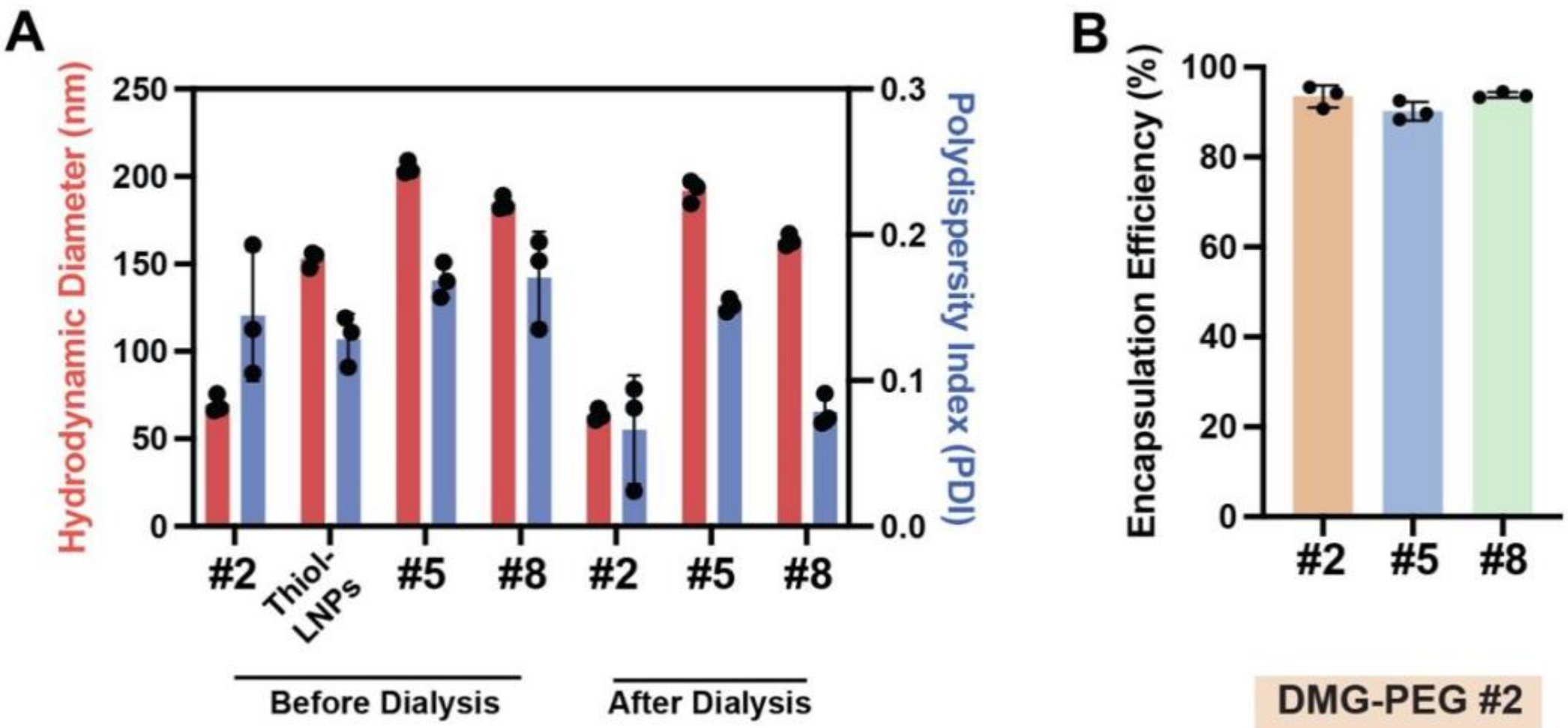

C Field Flow Fractionation Multi-Angle Light Scattering (FFF-MALS)

Sample ID	Nanoparticle Concentration (LNPs/mL)
DMG-PEG #2	4.39×10^{11}
OPSS-PEG #5	1.79×10^{11}
OPSS-HEP #8	1.22×10^{11}

Figure S3: Physicochemical characterization of DMG-PEG2000-, OPSS-PEG-, and OPSS-HEP-modified Vybrant™ DiO-labeled luciferase mRNA-LNPs at an N/P ratio of 8. (A) HDD and PDI of the indicated mRNA-LNP formulations measured by DLS. **(B)** Encapsulation efficiency (%EE) of mRNA within the LNPs. Data are presented as mean ± SD (n=3). **(C)** LNP concentration of mRNA-LNPs determined by field-flow fractionation coupled with multi-angle light scattering (FFF-MALS).

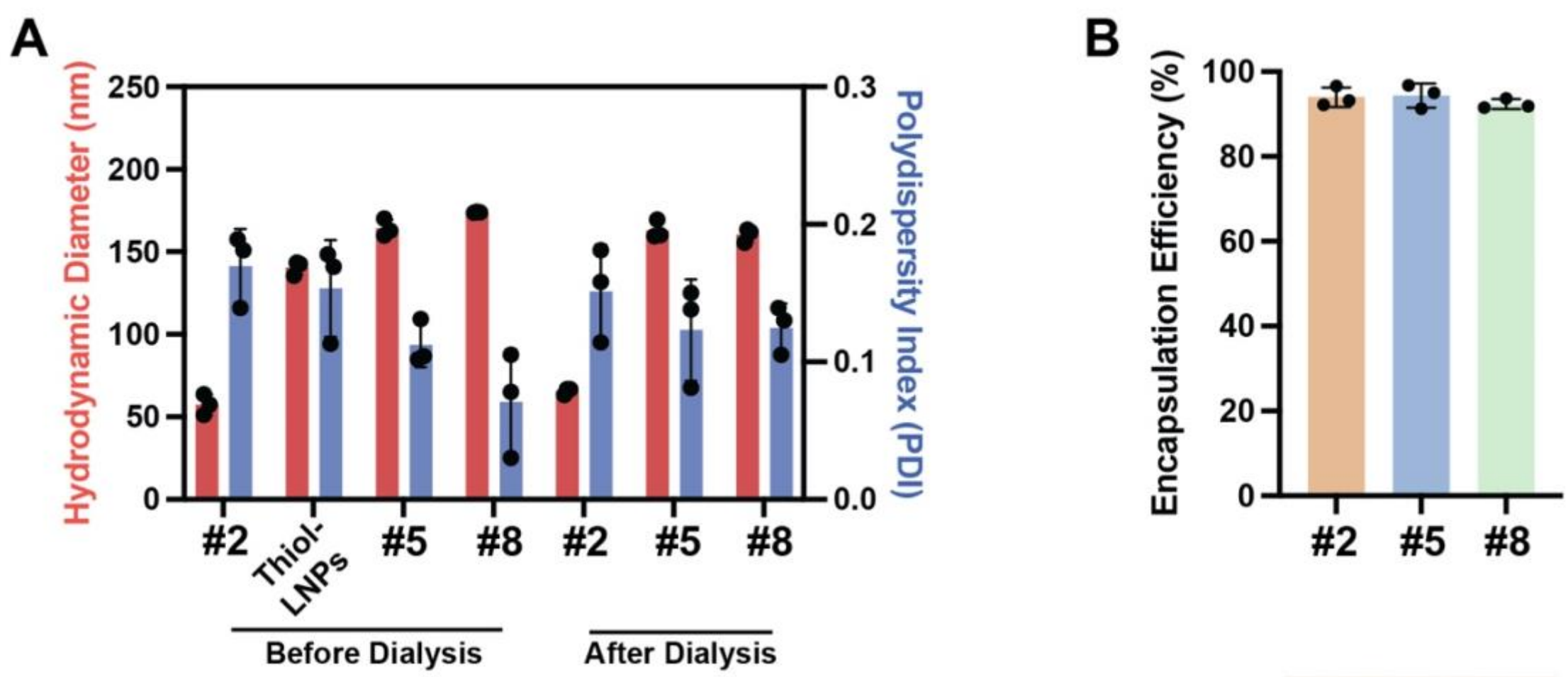

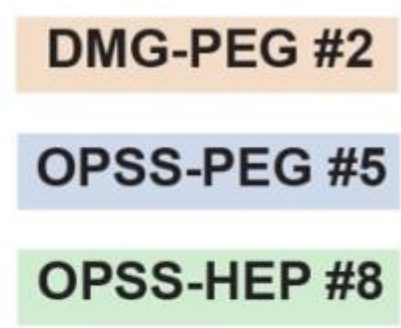

C Field Flow Fractionation Multi-Angle Light Scattering (FFF-MALS)

Sample ID	Nanoparticle Concentration (LNPs/mL)
DMG-PEG #2	3.61×10^{11}
OPSS-PEG #5	9.57×10^{10}
OPSS-HEP #8	1.24×10^{11}

Figure S4: Physicochemical characterization of DMG-PEG2000 (#2) -, OPSS-PEG (#5) -, and OPSS-HEP (#8) - modified EGFP mRNA-LNPs at an N/P ratio of 8. (A) HDD and PDI of the indicated mRNA-LNP formulations measured by dynamic light scattering (DLS). **(B)** %EE of mRNA. Data represent mean ± SD (n=3). **(C)** LNP concentration of mRNA-LNPs determined by FFF-MALS.

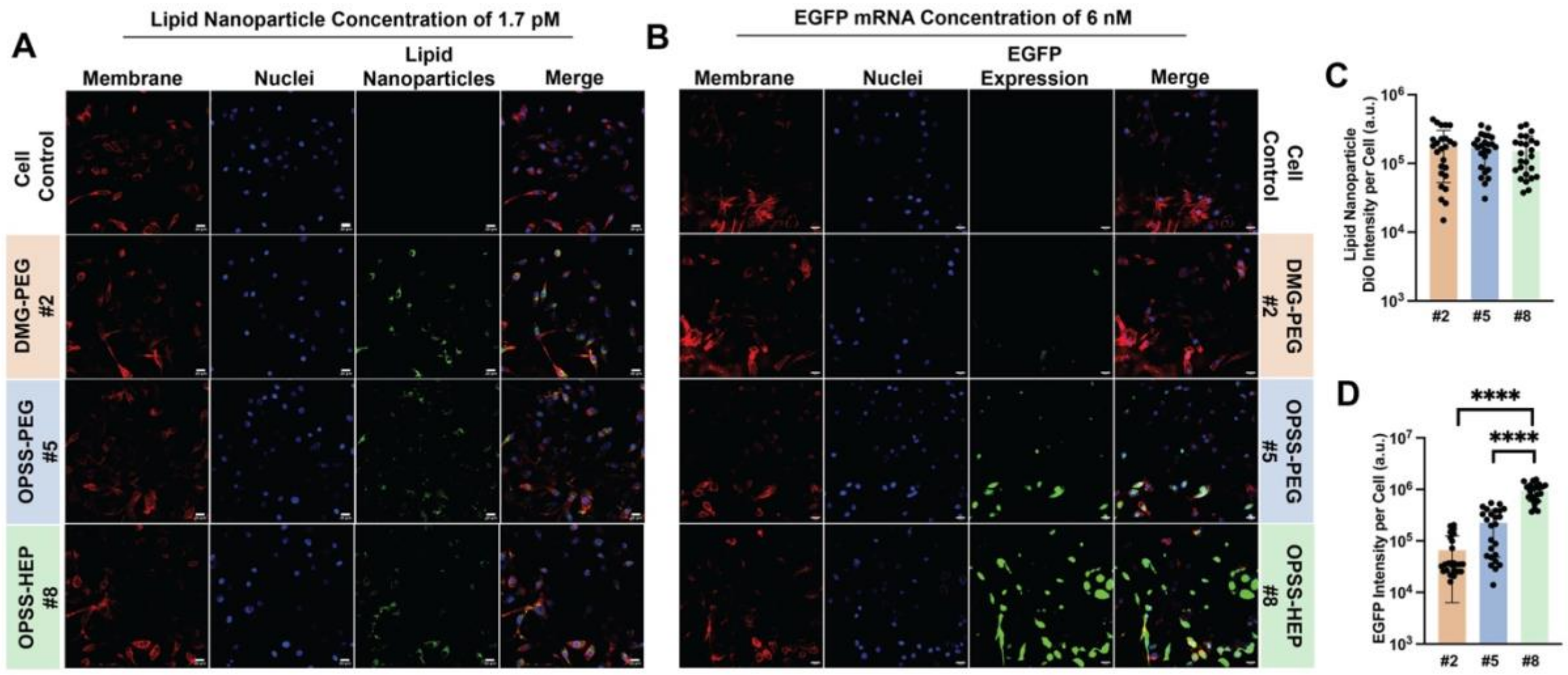

Figure S5. Surface modification enhances mRNA delivery efficiency without altering cellular uptake of EGFP mRNA-loaded LNPs. (A) Representative confocal laser scanning microscopy (CLSM) images of RAW 264.7 macrophages following 24-h incubation with Vybrant™ DiO-labeled DMG-PEG2000 (#2)-, OPSS-PEG (#5)-, or OPSS-HEP (#8)-modified mRNA-LNPs (1.7 pM LNPs per well, normalized based on FFF-MALS nanoparticle number quantification). The LNPs are shown in green, cell membranes in red (WGA-CF633), and cell nuclei in blue (DAPI). Scale bar represents 20 µm. **(B)** Representative CLSM images of RAW 264.7 macrophages 24 h after transfection with DMG-PEG2000 (#2)-, OPSS-PEG (#5)-, or OPSS-HEP (#8)-modified EGFP mRNA-LNPs (6 nM mRNA). EGFP expression is shown in green. Scale bar represents 20 µm. **(C)** Quantification of cellular uptake based on DiO fluorescence intensity measured from the CLSM images shown in panel A. Data are presented as mean ± SD (n=25 cells). No statistically significant differences in cellular uptake were observed among formulations (one-way ANOVA, $p>0.05$). **(D)** Quantification of EGFP fluorescence intensity measured from the CLSM images shown in panel B. Data represent mean ± SD (n=25). The OPSS-HEP-modified mRNA-LNPs exhibited significantly greater EGFP expression than the PEGylated controls (one-way ANOVA, ****$p<0.0001$).

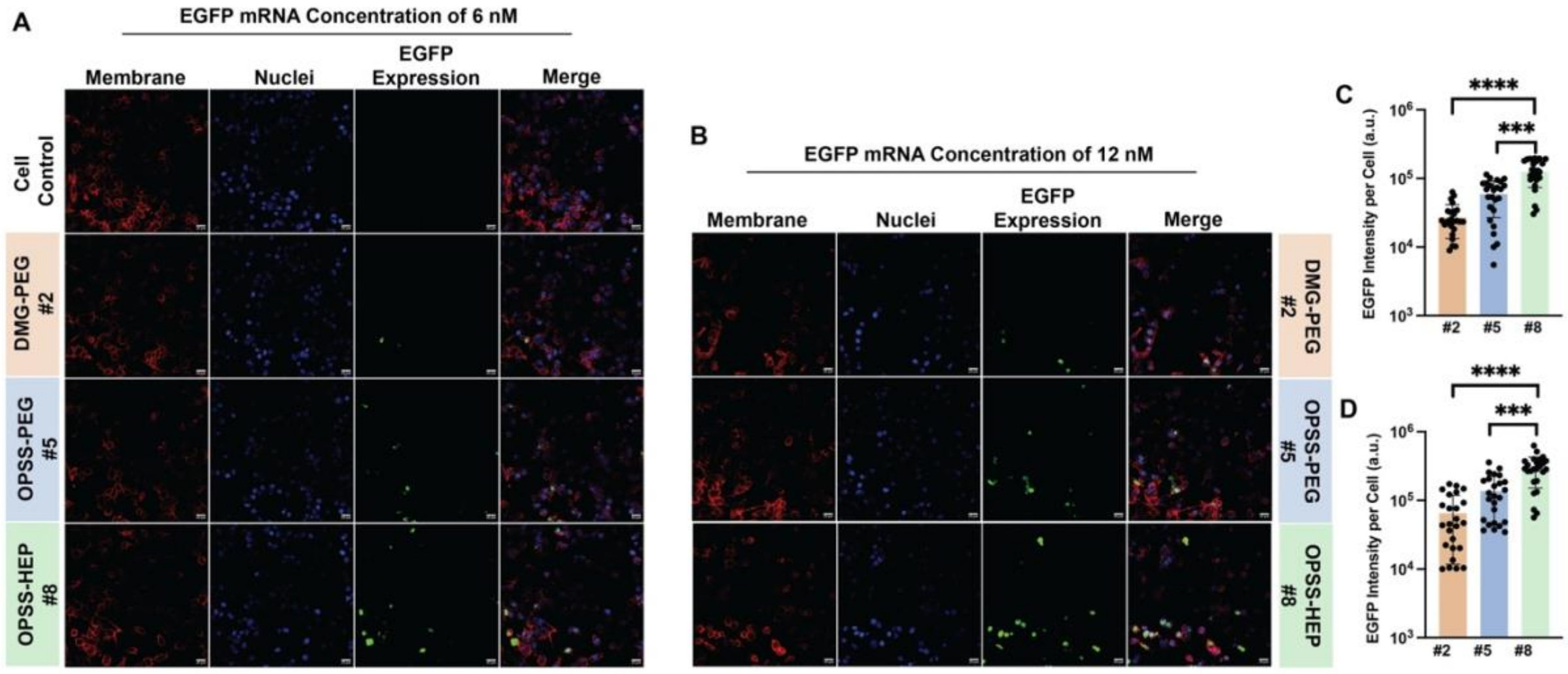

Figure S6. Cellular transfection efficiency of surface-modified EGFP mRNA-loaded lipid nanoparticles in DC2.4 dendritic cells. (A) Representative CLSM images of DC2.4 murine dendritic cells 24 h after transfection with DMG-PEG2000 (#2) -, OPSS-PEG (#5) -, or OPSS-HEP (#8) - modified EGFP mRNA-LNPs at an mRNA concentration of 6 nM. The EGFP fluorescence is shown in green. Scale bars represent 20 µm. **(B)** Representative CLSM images of DC2.4 cells 24 h after transfection with DMG-PEG2000 (#2) -, OPSS-PEG (#5) -, or OPSS-HEP (#8) - modified EGFP mRNA-LNPs at an mRNA concentration of 12 nM. The EGFP fluorescence is shown in green. Scale bars represent 20 µm. **(C)** Quantification of EGFP fluorescence intensity measured from panel A. Data represent mean ± SD (n=25). The OPSS-HEP-modified mRNA-LNPs exhibited significantly greater EGFP expression than DMG-PEG2000- and OPSS-PEG-modified counterparts. **(D)** Quantification of EGFP fluorescence intensity measured from panel B. Data represent mean ± SD (n=25). The OPSS-HEP-modified mRNA-LNPs exhibited significantly greater EGFP expression than DMG-PEG2000- and OPSS-PEG-modified counterparts. Statistical significance was determined by one-way ANOVA with multiple comparisons. ***$p<0.001$; ****$p<0.0001$.

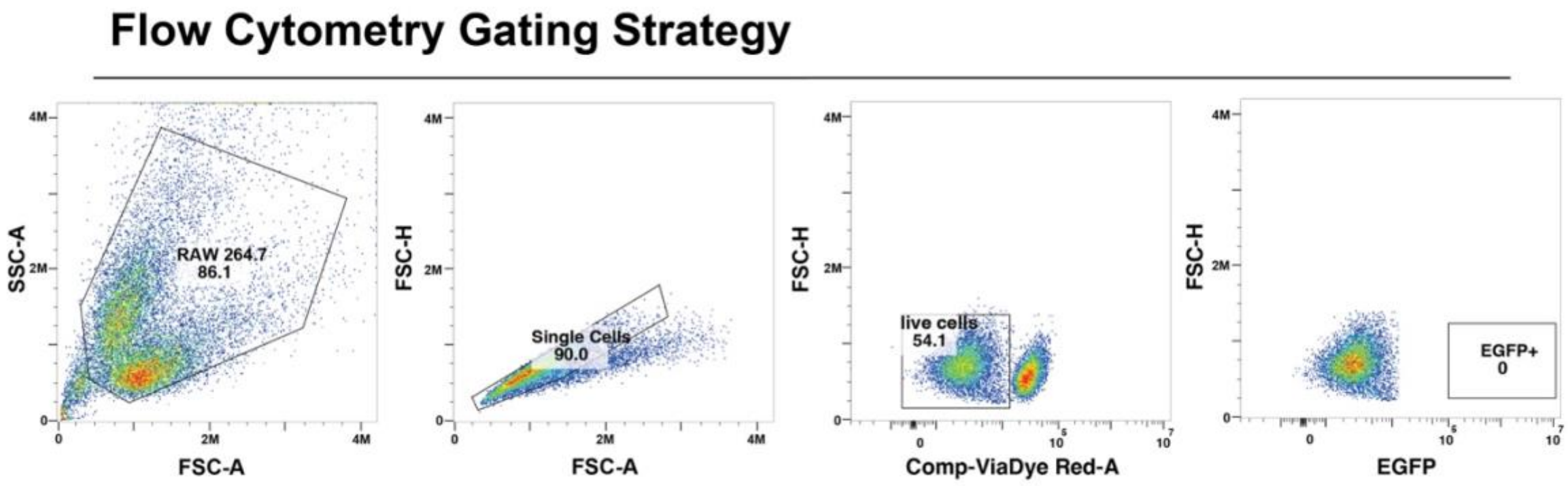

Figure S7. Flow cytometry gating strategy for analyzing EGFP-positive RAW 264.7 cells. Sequential gating was performed to identify RAW 264.7 cells, followed by singlet discrimination, exclusion of nonviable cells, and identification of EGFP-positive cell populations. The resulting EGFP-positive populations were used to quantify transfection efficiencies (% EGFP-positive cells) and mean EGFP fluorescence intensities.

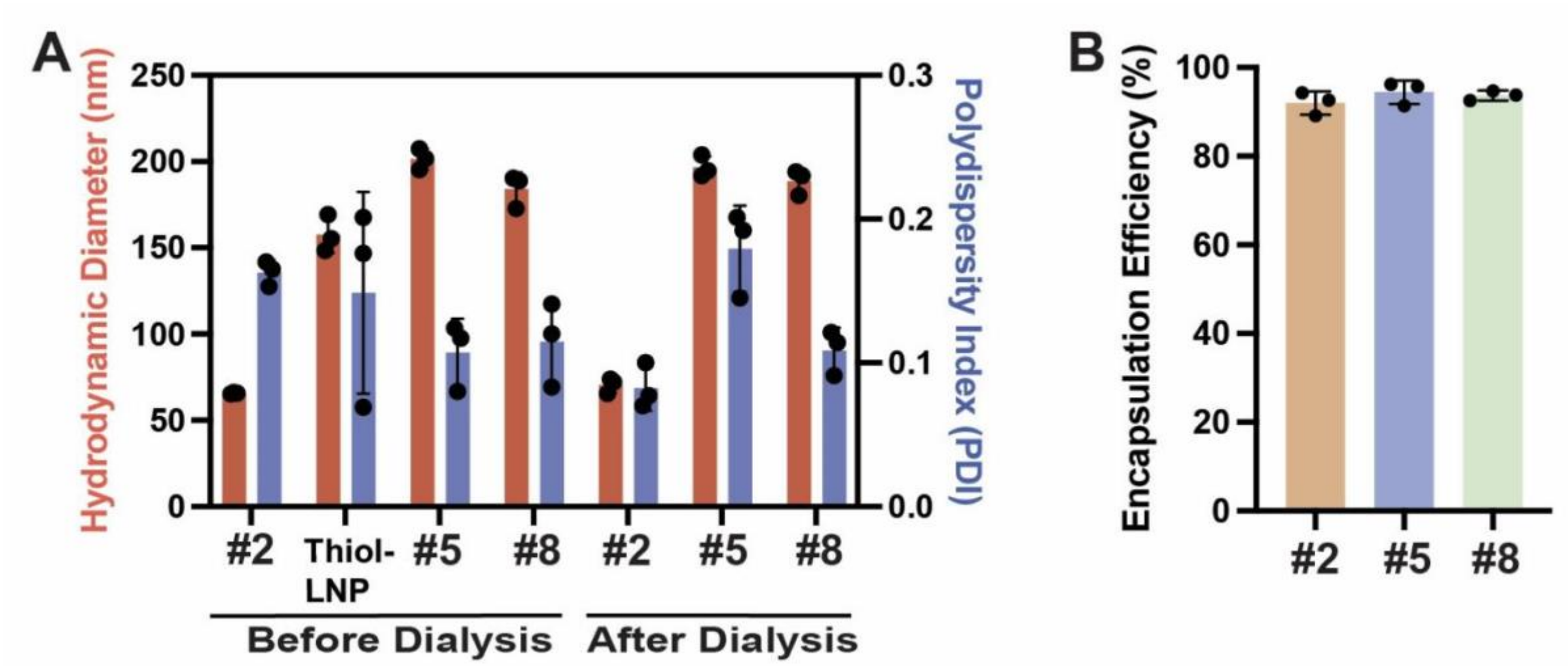

Figure S8: Physicochemical characterization of DMG-PEG2000-, OPSS-PEG-, and OPSS-HEP-modified with lipophilic carbocyanine fluorescent dye DiD-labeled EGFP mRNA-LNPs used for live-cell imaging at an N/P ratio of 8. (A) HDD and PDI of the indicated mRNA-LNP formulations measured by DLS. **(B)** %EE of mRNA. Data represent mean ± SD (n=3).

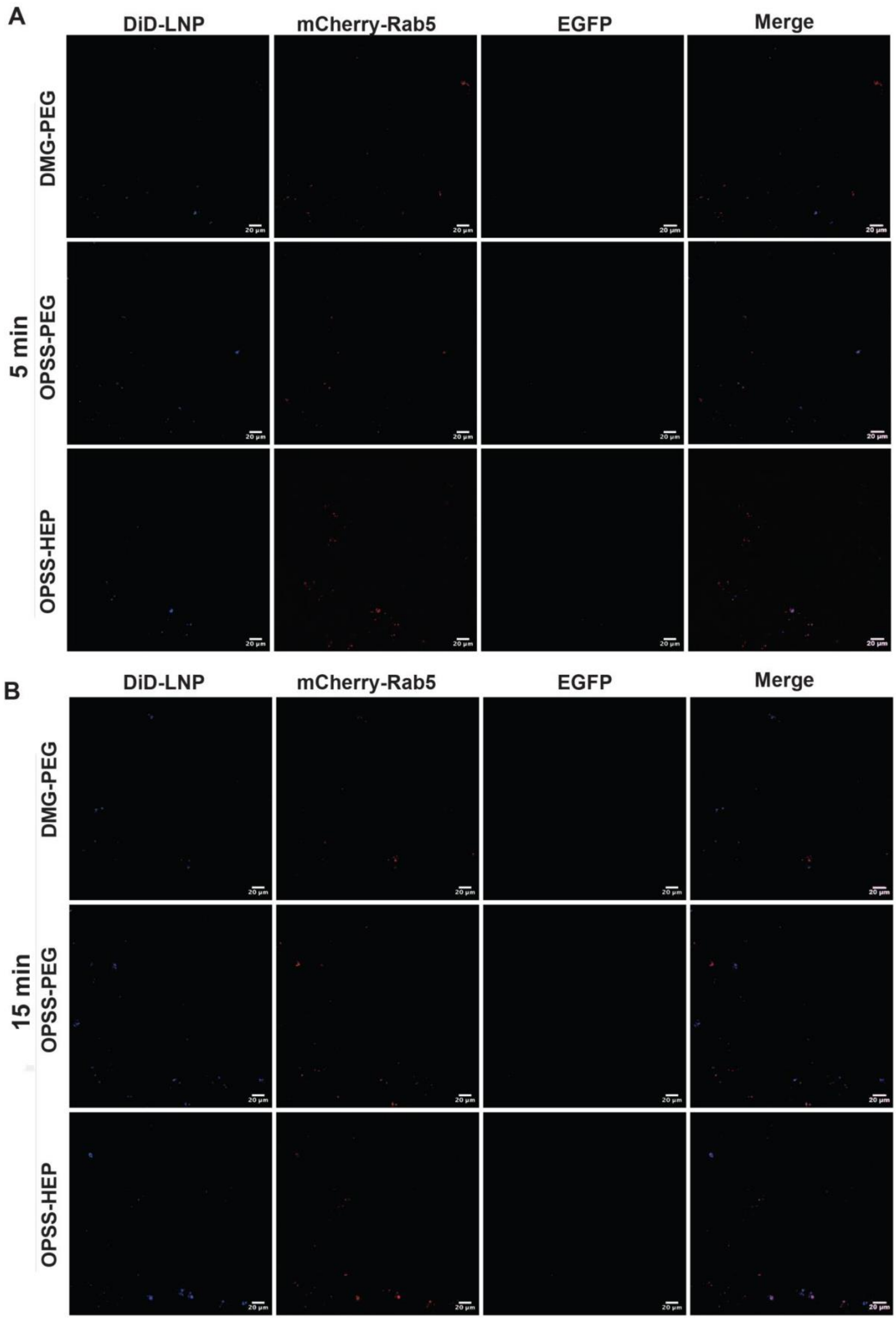
A
DiD-LNP
mCherry-Rab5
EGFP
Merge
5 min
DMG-PEG
OPSS-PEG
OPSS-HEP
B
DiD-LNP
mCherry-Rab5
EGFP
Merge
15 min
DMG-PEG
OPSS-PEG
OPSS-HEP
20 μm

Figure S9: Live-cell imaging and intracellular localization of DiD-labeled EGFP mRNA-LNPs with different surface modifications in DC2.4 cells during early endosomal transport (5 minutes and 15 minutes). (A) Representative CLSM images of DC2.4 murine dendritic cells expressing mCherry-Rab5 (early endosome marker) at 5 min following treatment with DiD-labeled EGFP mRNA-LNPs coated with DMG-PEG2000, OPSS-PEG, or OPSS-HEP. Scale bars represent 20 μm. **(B)** Representative CLSM images showing mCherry-Rab5-associated intracellular trafficking at 15 min post-treatment. Scale bars represent 20 μm.

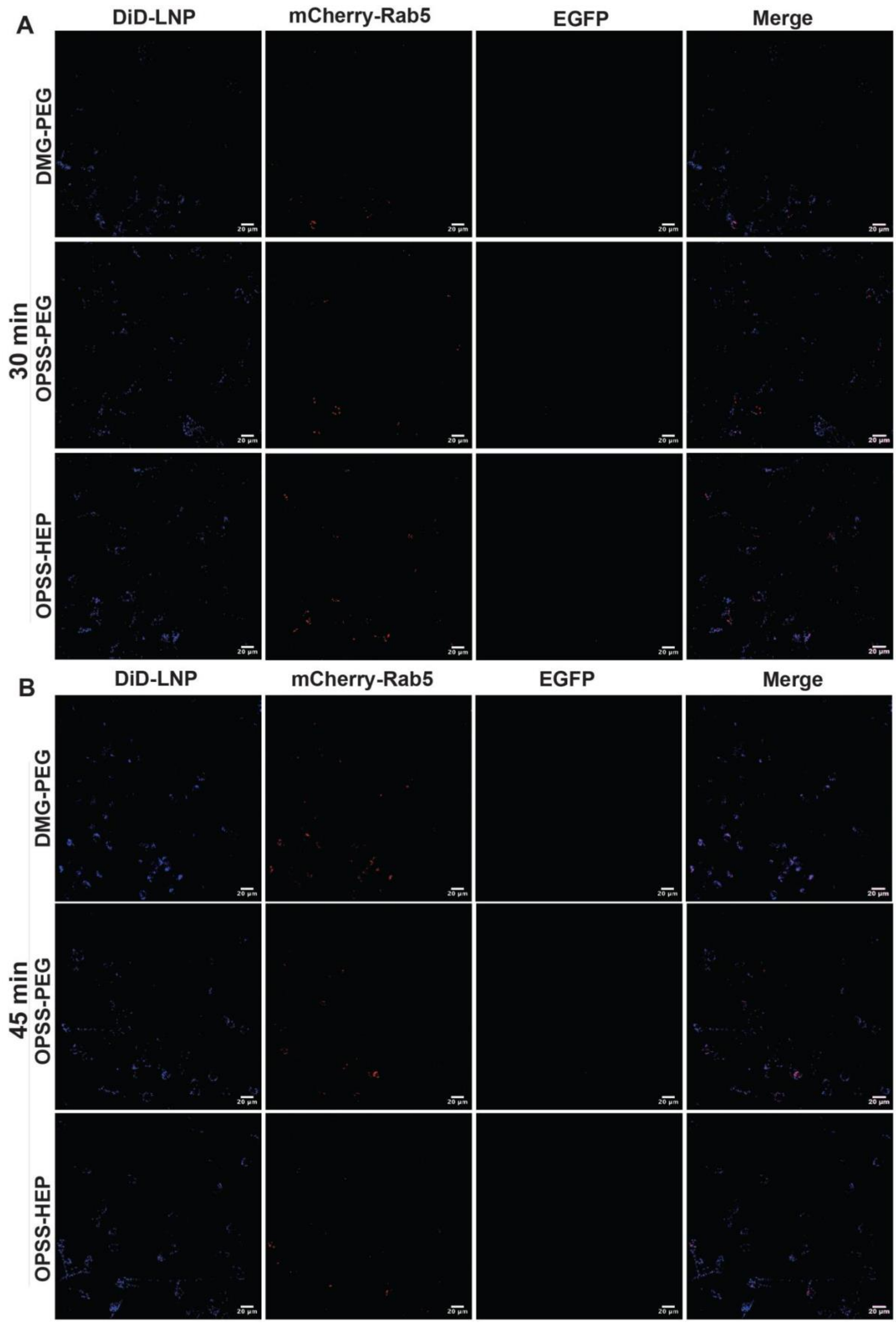
A
DiD-LNP
mCherry-Rab5
EGFP
Merge
DMG-PEG
30 min
OPSS-PEG
OPSS-HEP
20 μm
B
DiD-LNP
mCherry-Rab5
EGFP
Merge
DMG-PEG
45 min
OPSS-PEG
OPSS-HEP
20 μm

Figure S10: Live-cell imaging and intracellular localization of DiD-labeled EGFP mRNA-LNPs with different surface modifications in DC2.4 cells during early endosomal transport (30 minutes and 45 minutes). (A) Representative CLSM images showing mCherry-Rab5-associated intracellular trafficking at 30 min following treatment with DiD-labeled EGFP mRNA-LNPs coated with DMG-PEG2000, OPSS-PEG, or OPSS-HEP. Scale bars represent 20 µm. **(B)** Representative CLSM images showing mCherry-Rab5-associated intracellular trafficking at 45 min post-treatment. Scale bars represent 20 µm.

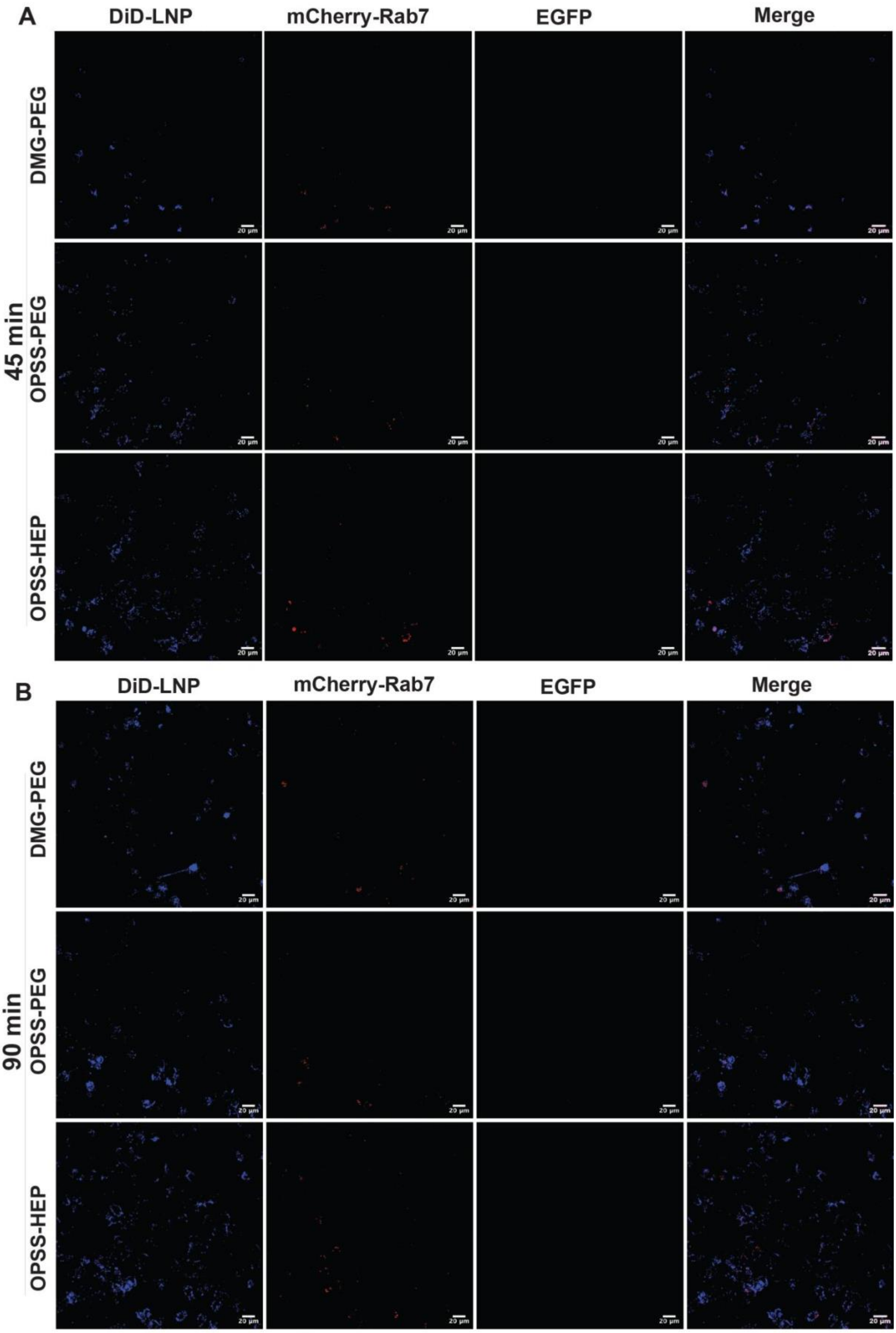
A
DiD-LNP
mCherry-Rab7
EGFP
Merge
45 min
DMG-PEG
OPSS-PEG
OPSS-HEP
20 μm
B
DiD-LNP
mCherry-Rab7
EGFP
Merge
90 min
DMG-PEG
OPSS-PEG
OPSS-HEP
20 μm

Figure S11: Live-cell imaging and intracellular localization of DiD-labeled EGFP mRNA-LNPs with different surface modifications in DC2.4 cells during late endosomal transport (45 minutes and 90 minutes). (A) Representative CLSM images of DC2.4 cells expressing mCherry-Rab7 (late endosome marker) at 45 min following treatment with DiD-labeled EGFP mRNA-LNPs coated with DMG-PEG2000, OPSS-PEG, or OPSS-HEP. Scale bars represent 20 µm. **(B)** Representative CLSM images showing mCherry-Rab7-associated intracellular trafficking at 90 min post-treatment. Scale bars represent 20 µm.

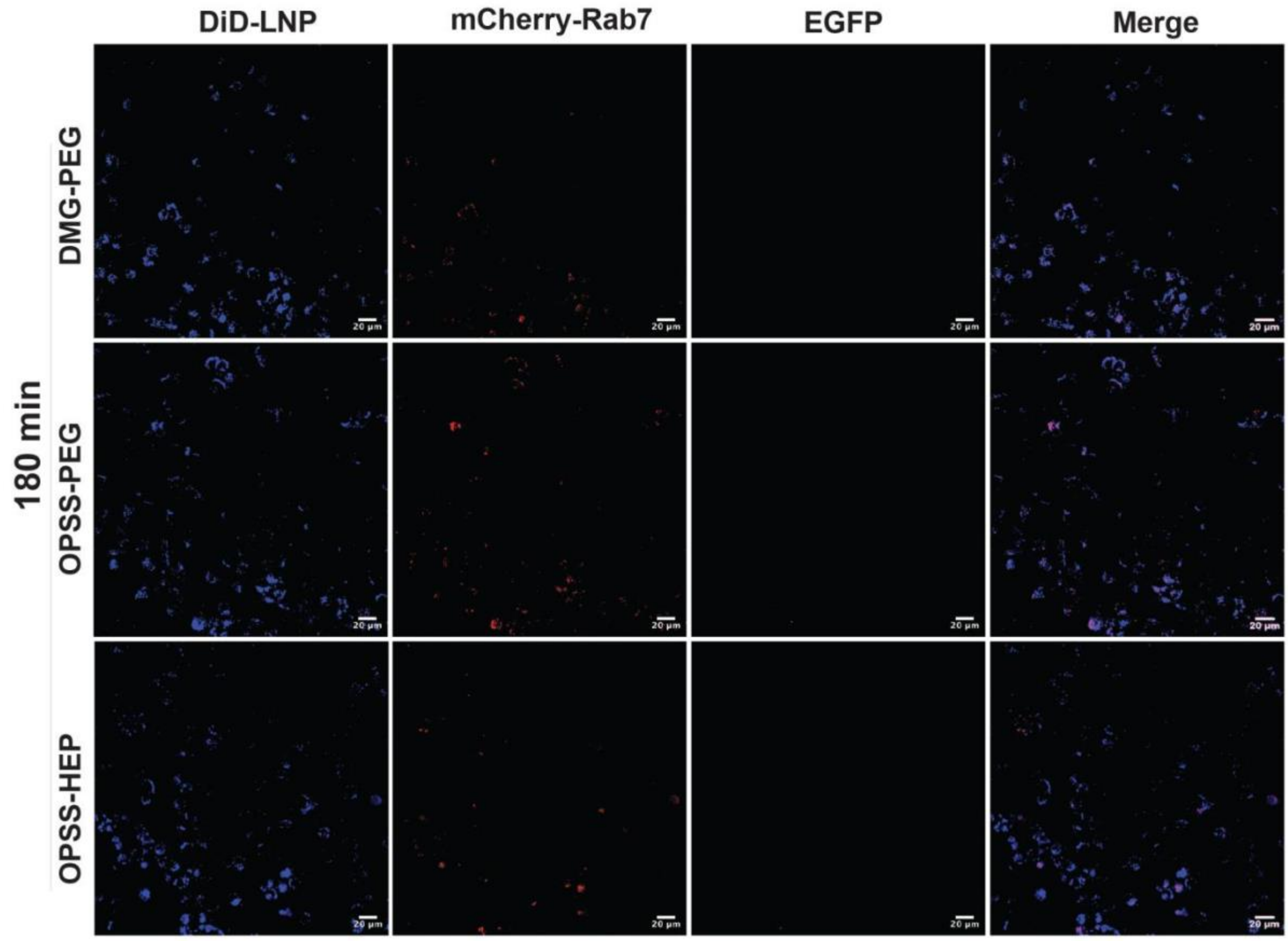

Figure S12: Live-cell imaging and intracellular localization of DiD-labeled EGFP mRNA-LNPs with different surface modifications in DC2.4 cells during late endosomal transport (180 minutes). Representative CLSM images showing mCherry-Rab7-associated intracellular trafficking at 180 min following treatment with DiD-labeled EGFP mRNA-LNPs coated with DMG-PEG2000, OPSS-PEG, or OPSS-HEP. Scale bars represent 20 µm.

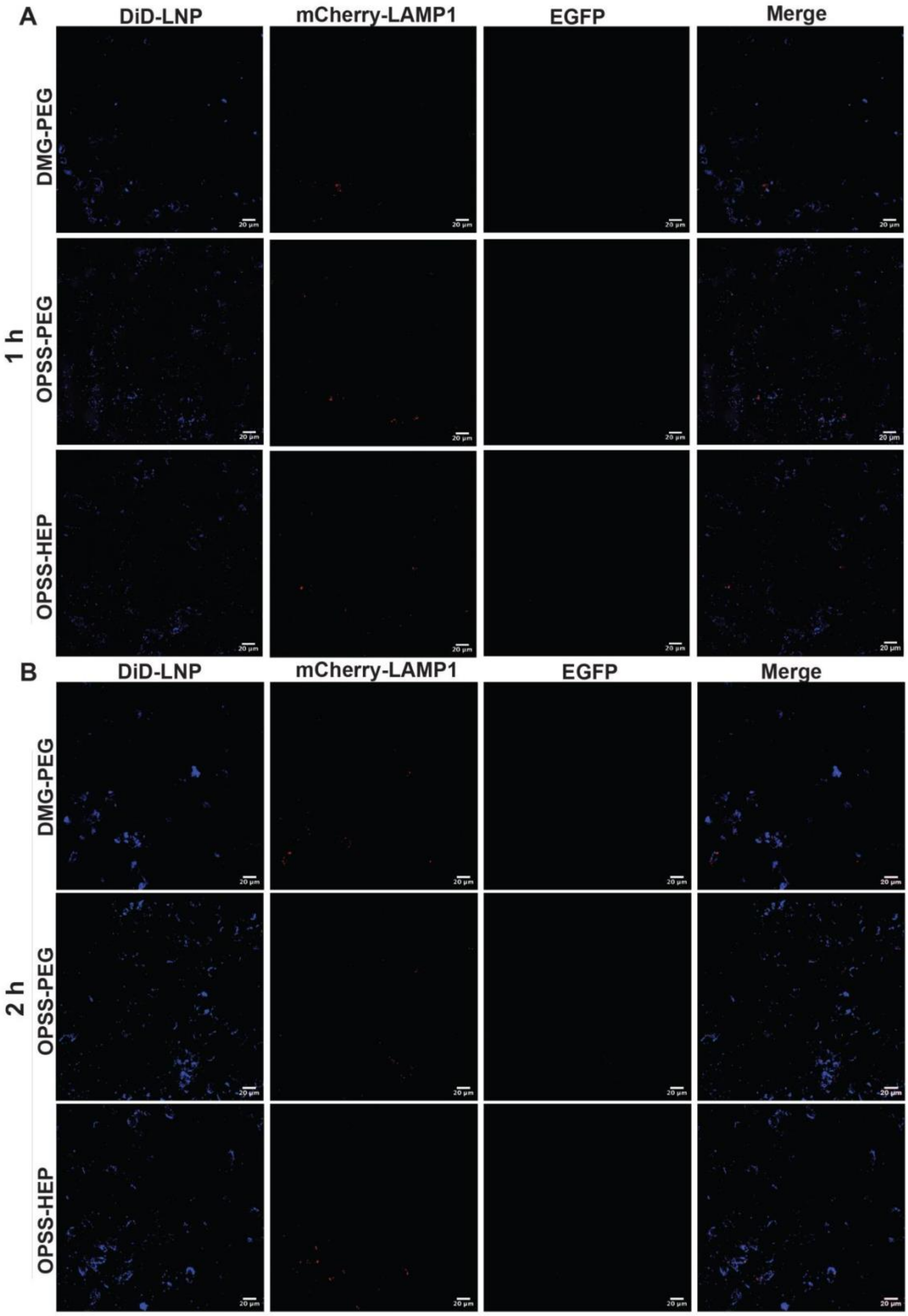

Figure S13: Live-cell imaging and intracellular localization of DiD-labeled EGFP mRNA-LNPs with different surface modifications in DC2.4 cells during lysosome transport (1 hour and 2 hours). (A) Representative CLSM images of DC2.4 cells expressing mCherry-

LAMP1 (lysosomal marker) at 1 h following treatment with DiD-labeled EGFP mRNA-LNPs coated with DMG-PEG2000, OPSS-PEG, or OPSS-HEP. Scale bars represent 20 µm. **(B)** Representative CLSM images showing mCherry-LAMP1-associated intracellular trafficking at 2 h post-treatment. Scale bars represent 20 µm.

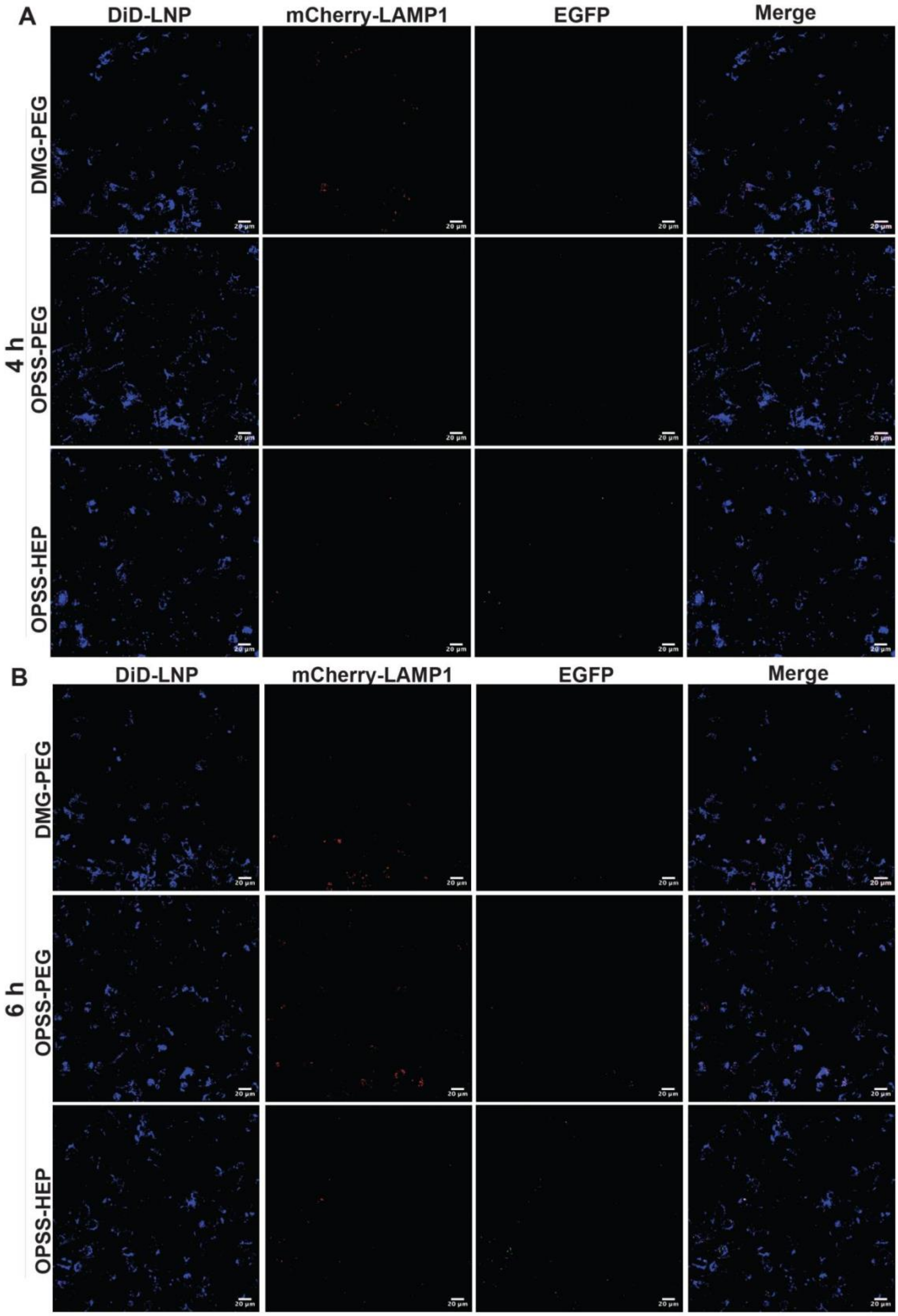

Figure S14: Live-cell imaging and intracellular localization of DiD-labeled EGFP mRNA-LNPs with different surface modifications in DC2.4 cells during lysosome transport (4

hours and 6 hours). (A) Representative CLSM images of DC2.4 cells expressing mCherry-LAMP1 (lysosomal marker) at 4 h following treatment with DiD-labeled EGFP mRNA-LNPs coated with DMG-PEG2000, OPSS-PEG, or OPSS-HEP. Scale bars represent 20 μm. **(B)** Representative CLSM images showing mCherry-LAMP1-associated intracellular trafficking at 6 h post-treatment. Scale bars represent 20 μm.

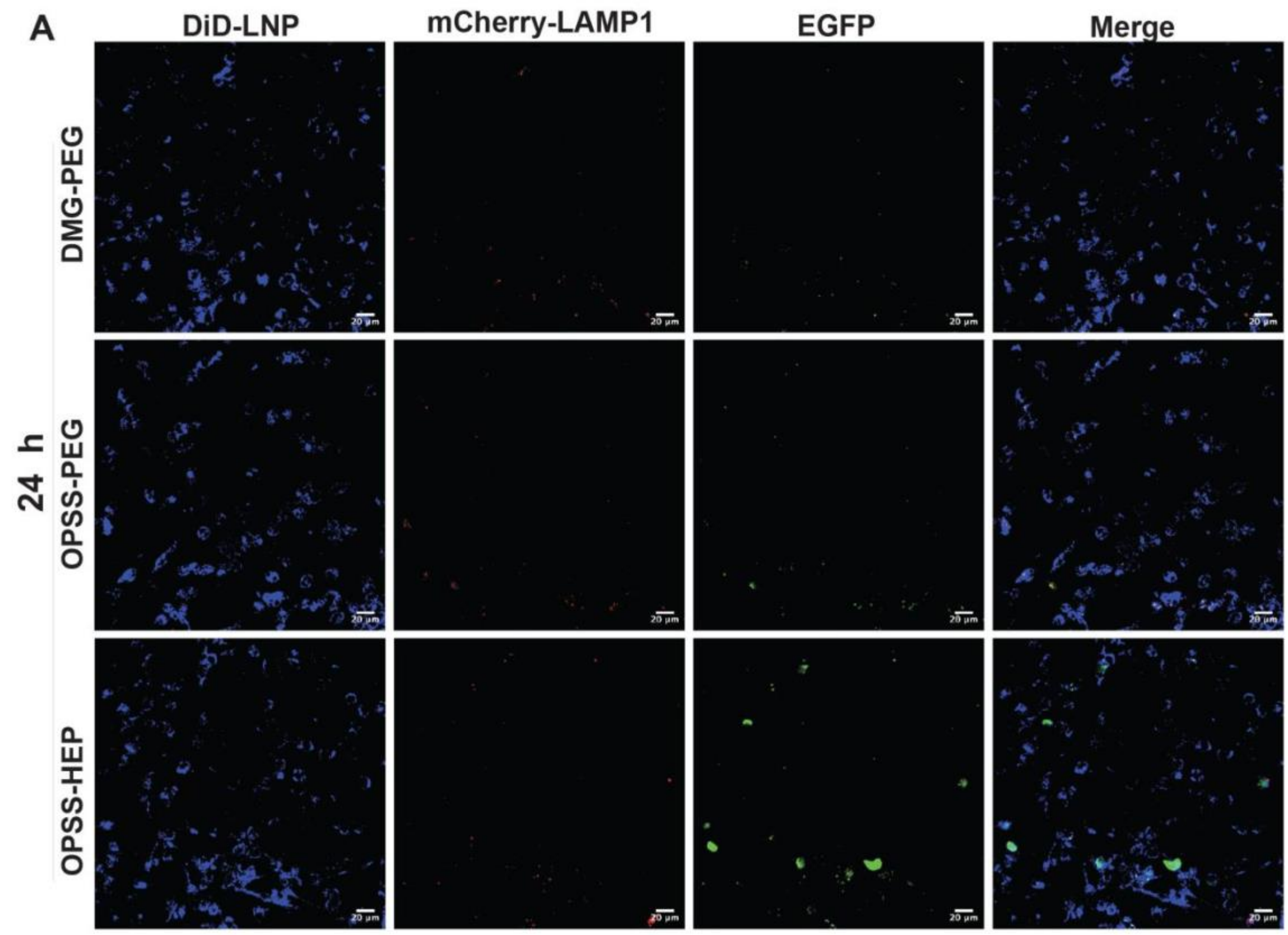

Figure S15: Live-cell imaging and intracellular localization of DiD-labeled EGFP mRNA-LNPs with different surface modifications in DC2.4 cells during lysosome transport (24 hours). (A) Representative CLSM images showing mCherry-LAMP1-associated intracellular trafficking at 24 h following treatment with DiD-labeled EGFP mRNA-LNPs coated with DMG-PEG2000, OPSS-PEG, or OPSS-HEP. Scale bars represent 20 µm.

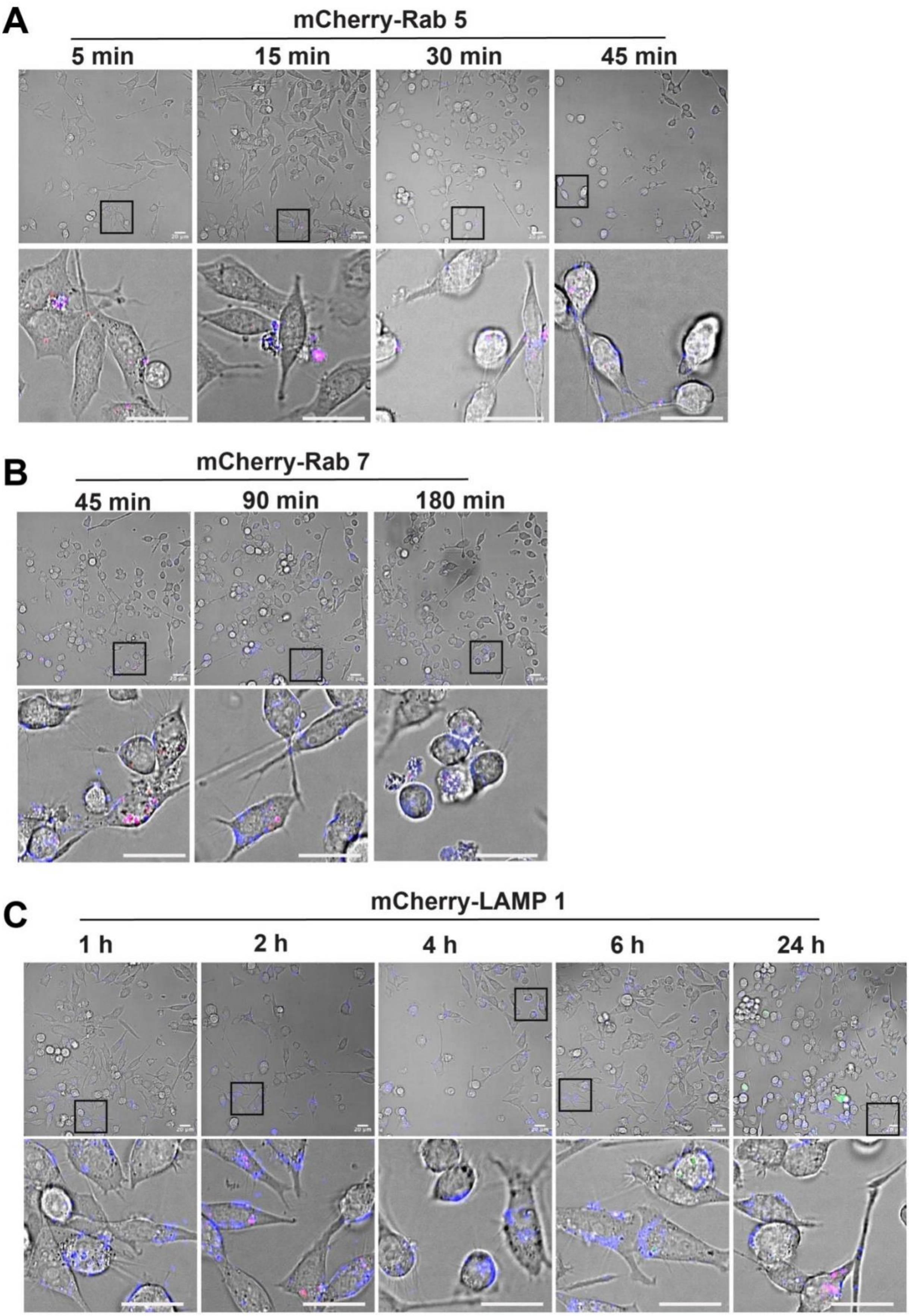
A
mCherry-Rab 5
5 min
15 min
30 min
45 min
B
mCherry-Rab 7
45 min
90 min
180 min
C
mCherry-LAMP 1
1 h
2 h
4 h
6 h
24 h

Figure S16: Time-lapse live-cell imaging of intracellular trafficking and localization of OPSS-HEP-modified mRNA-LNPs in DC2.4 dendritic cells. Representative time-lapse CLSM images showing the intracellular trafficking behavior and subcellular localization of DiD-labeled EGFP mRNA-LNPs in DC2.4 cells over a 24 h period following treatment with OPSS-HEP-modified LNPs. **(A)** mCherry-Rab5 labels are shown in red, DiD-labeled mRNA-LNPs are shown in blue. **(B)** mCherry-Rab7 labels are shown in red, DiD-labeled mRNA-LNPs are shown in blue. **(C)** mCherry-LAMP1 labels are shown in red, DiD-labeled mRNA-LNPs are shown in blue, and EGFP is shown in green. The corresponding bright-field images are included to visualize cell morphology and monitor intracellular nanoparticle movement throughout the imaging period. Scale bars represent 20 μm.

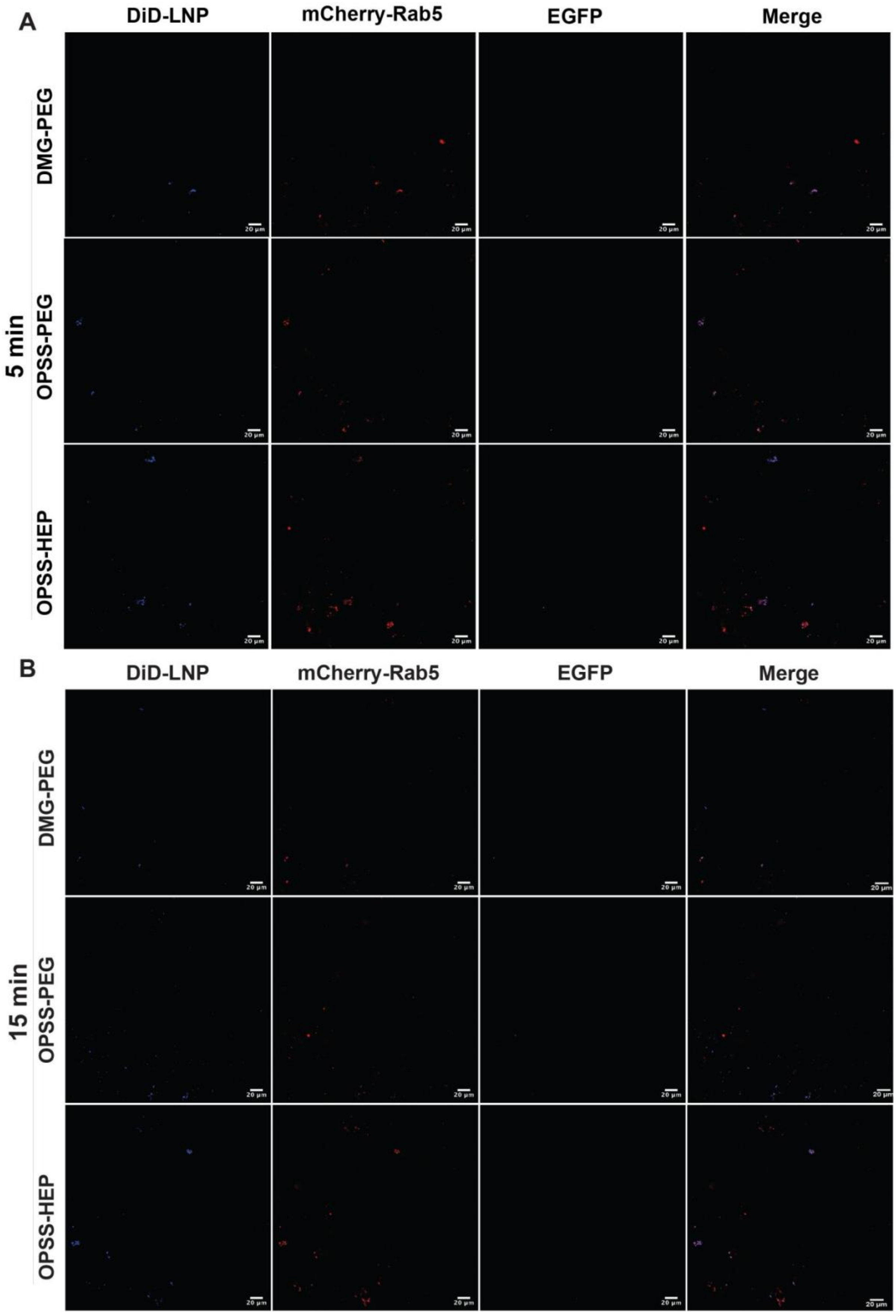
A
DiD-LNP
mCherry-Rab5
EGFP
Merge
5 min
DMG-PEG
OPSS-PEG
OPSS-HEP
20 µm
B
DiD-LNP
mCherry-Rab5
EGFP
Merge
15 min
DMG-PEG
OPSS-PEG
OPSS-HEP
20 µm

Figure S17: Live-cell imaging and intracellular localization of DiD-labeled EGFP mRNA-LNPs with different surface modifications in RAW 264.7 cells during early endosomal transport (5 minutes and 15 minutes). (A) Representative CLSM images of RAW 264.7 cells expressing mCherry-Rab5 (early endosome marker) at 5 min following treatment with DiD-labeled EGFP mRNA-LNPs coated with DMG-PEG2000, OPSS-PEG, or OPSS-HEP. Scale bars represent 20 µm. **(B)** Representative CLSM images showing mCherry-Rab5-associated intracellular trafficking at 15 min post-treatment. Scale bars represent 20 µm.

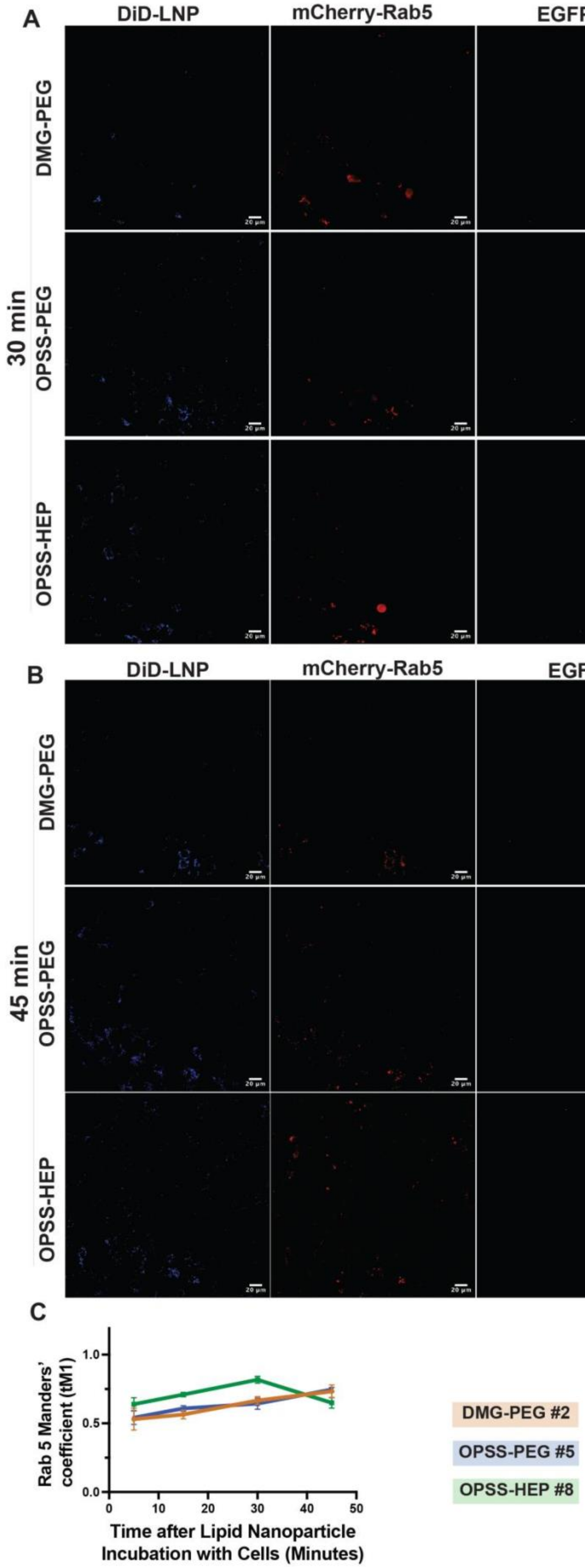
A
DiD-LNP
mCherry-Rab5
EGFP
Merge
DMG-PEG
30 min
OPSS-PEG
OPSS-HEP
B
DiD-LNP
mCherry-Rab5
EGFP
Merge
DMG-PEG
45 min
OPSS-PEG
OPSS-HEP
C
Rab 5 Manders' coefficient (tM1)
1.0
0.5
0.0
0
10
20
30
40
50
Time after Lipid Nanoparticle Incubation with Cells (Minutes)
DMG-PEG #2
OPSS-PEG #5
OPSS-HEP #8

Figure S18: Live-cell imaging and intracellular localization of DiD-labeled EGFP mRNA-LNPs with different surface modifications in RAW 264.7 cells during early endosomal transport (30 minutes and 45 minutes). (A) Representative CLSM images showing mCherry-Rab5-associated intracellular trafficking at 30 min following treatment with DiD-labeled EGFP mRNA-LNPs coated with DMG-PEG2000, OPSS-PEG, or OPSS-HEP. Scale bars represent 20 µm. **(B)** Representative CLSM images showing mCherry-Rab5-associated intracellular trafficking at 45 min post-treatment. Scale bars represent 20 µm. **(C)** Quantification of co-localization between DiD-labeled LNPs and mCherry-Rab5-positive early endosomes using Manders' coefficient (tM1) from 5 min to 45 min. Data represent mean ± SD (n=3), the solid lines are a guide to the eye.

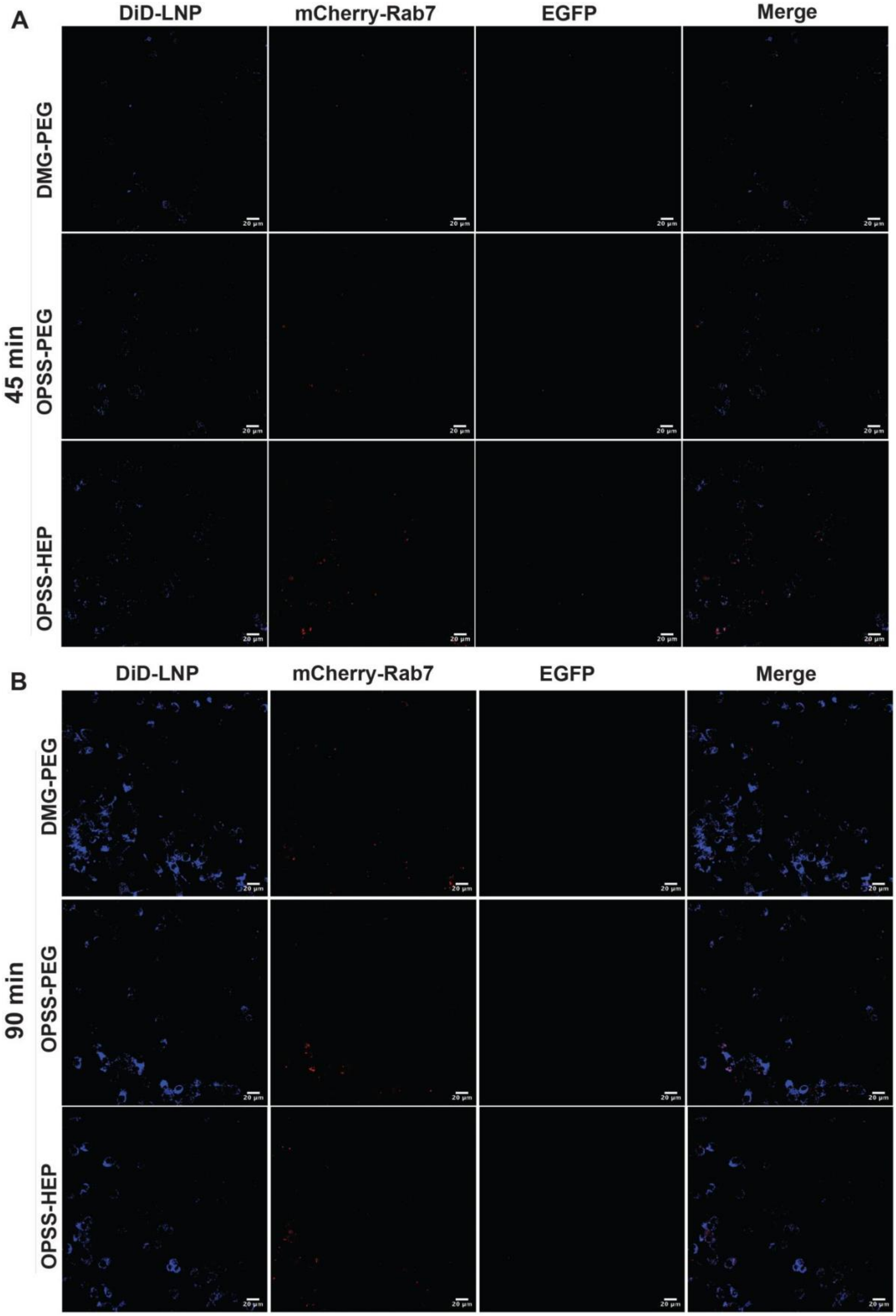
A
DiD-LNP
mCherry-Rab7
EGFP
Merge
45 min
DMG-PEG
OPSS-PEG
OPSS-HEP
B
DiD-LNP
mCherry-Rab7
EGFP
Merge
90 min
DMG-PEG
OPSS-PEG
OPSS-HEP

Figure S19: Live-cell imaging and intracellular localization of DiD-labeled EGFP mRNA-LNPs with different surface modifications in RAW 264.7 cells during late endosomal transport (45 minutes and 90 minutes). (A) Representative CLSM images of RAW 264.7 cells expressing mCherry-Rab7 (late endosome marker) at 45 min following treatment with DiD-labeled EGFP mRNA-LNPs coated with DMG-PEG2000, OPSS-PEG, or OPSS-HEP. Scale bars represent 20 μm. **(B)** Representative CLSM images showing mCherry-Rab7-associated intracellular trafficking at 90 min post-treatment. Scale bars represent 20 μm.

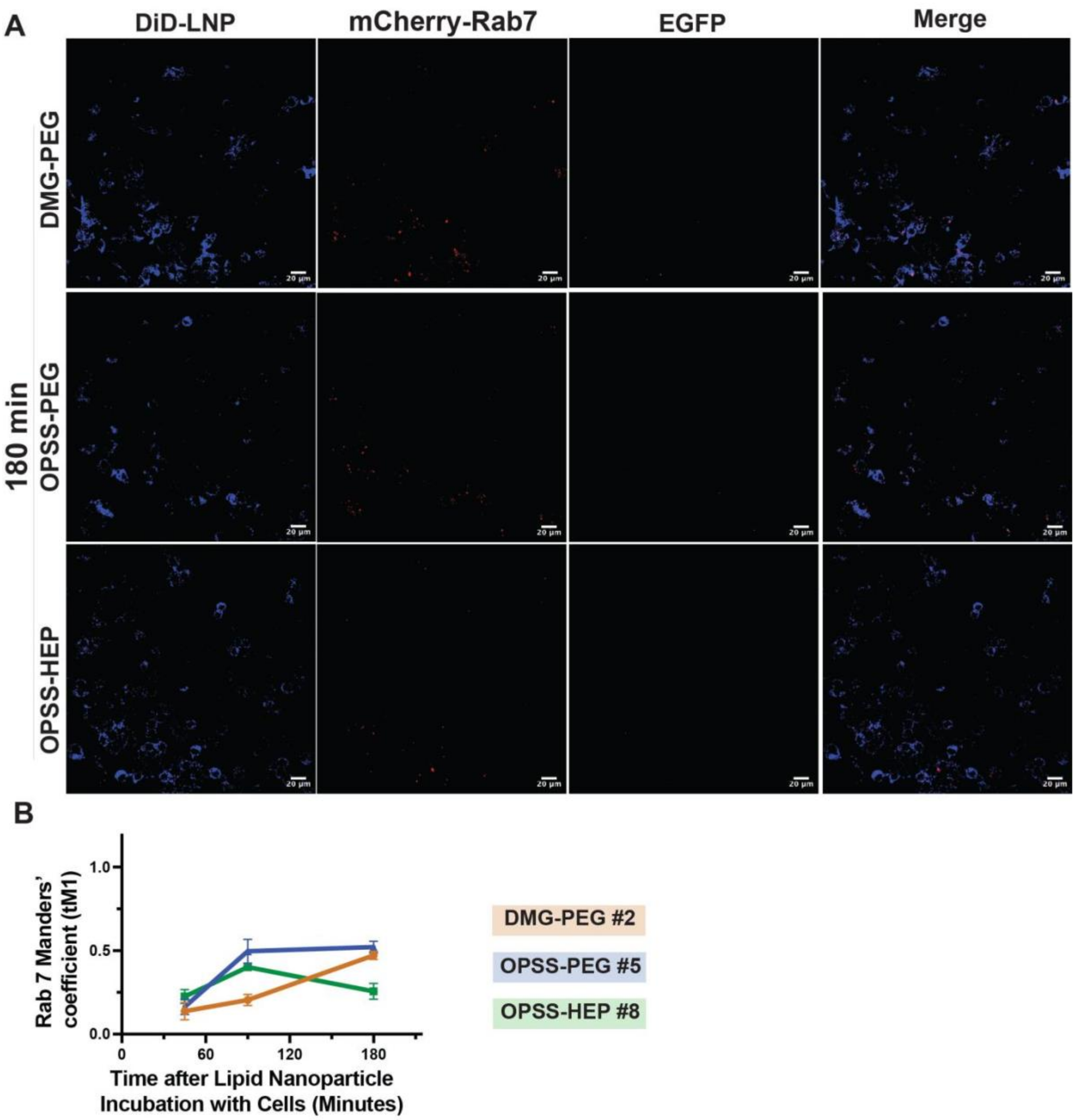

Figure S20: Live-cell imaging and intracellular localization of DiD-labeled EGFP mRNA-LNPs with different surface modifications in RAW 264.7 cells during late endosomal transport (180 minutes). (A) Representative CLSM images showing mCherry-Rab7-associated intracellular trafficking at 180 min following treatment with DiD-labeled EGFP mRNA-LNPs coated with DMG-PEG2000, OPSS-PEG, or OPSS-HEP. Scale bars represent 20 µm. **(B)** Quantification of colocalization between DiD-labeled LNPs and mCherry-Rab7-positive late endosomes using Manders' coefficient (tM1) from 45 min to 180 min. Data represent mean ± SD (n=3), the solid lines are a guide to the eye.

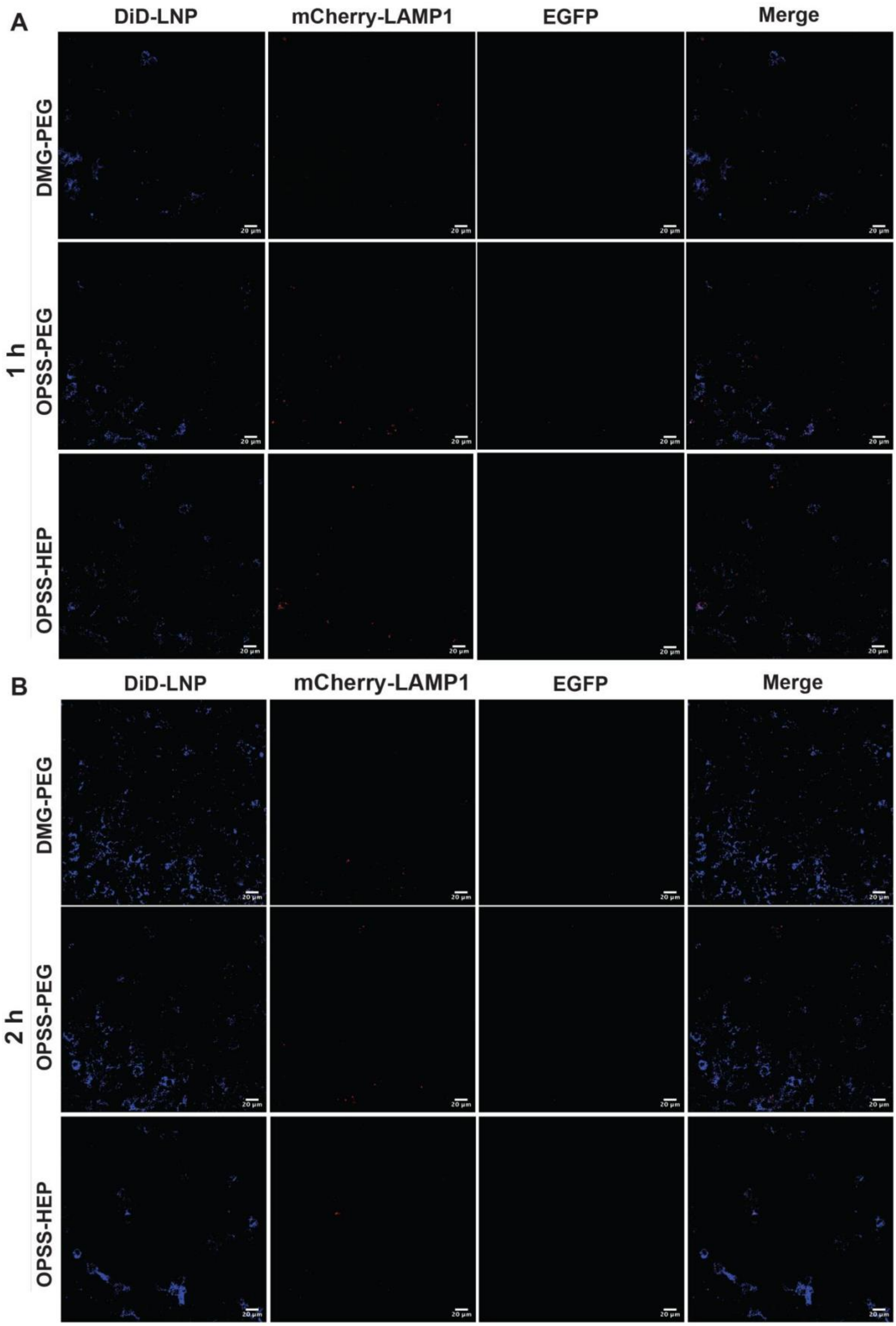
A
DiD-LNP
mCherry-LAMP1
EGFP
Merge
DMG-PEG
OPSS-PEG
OPSS-HEP
1 h
20 μm
B
DiD-LNP
mCherry-LAMP1
EGFP
Merge
DMG-PEG
OPSS-PEG
OPSS-HEP
2 h
20 μm

Figure S21: Live-cell imaging and intracellular localization of DiD-labeled EGFP mRNA-LNPs with different surface modifications in RAW 264.7 cells during lysosome transport (1 hour and 2 hours). (A) Representative CLSM images of RAW 264.7 cells expressing mCherry-LAMP1 (lysosomal marker) at 1 h following treatment with DiD-labeled EGFP mRNA-LNPs coated with DMG-PEG2000, OPSS-PEG, or OPSS-HEP. Scale bars represent 20 μm. **(B)** Representative CLSM images showing mCherry-LAMP1-associated intracellular trafficking at 2 h post-treatment. Scale bars represent 20 μm.

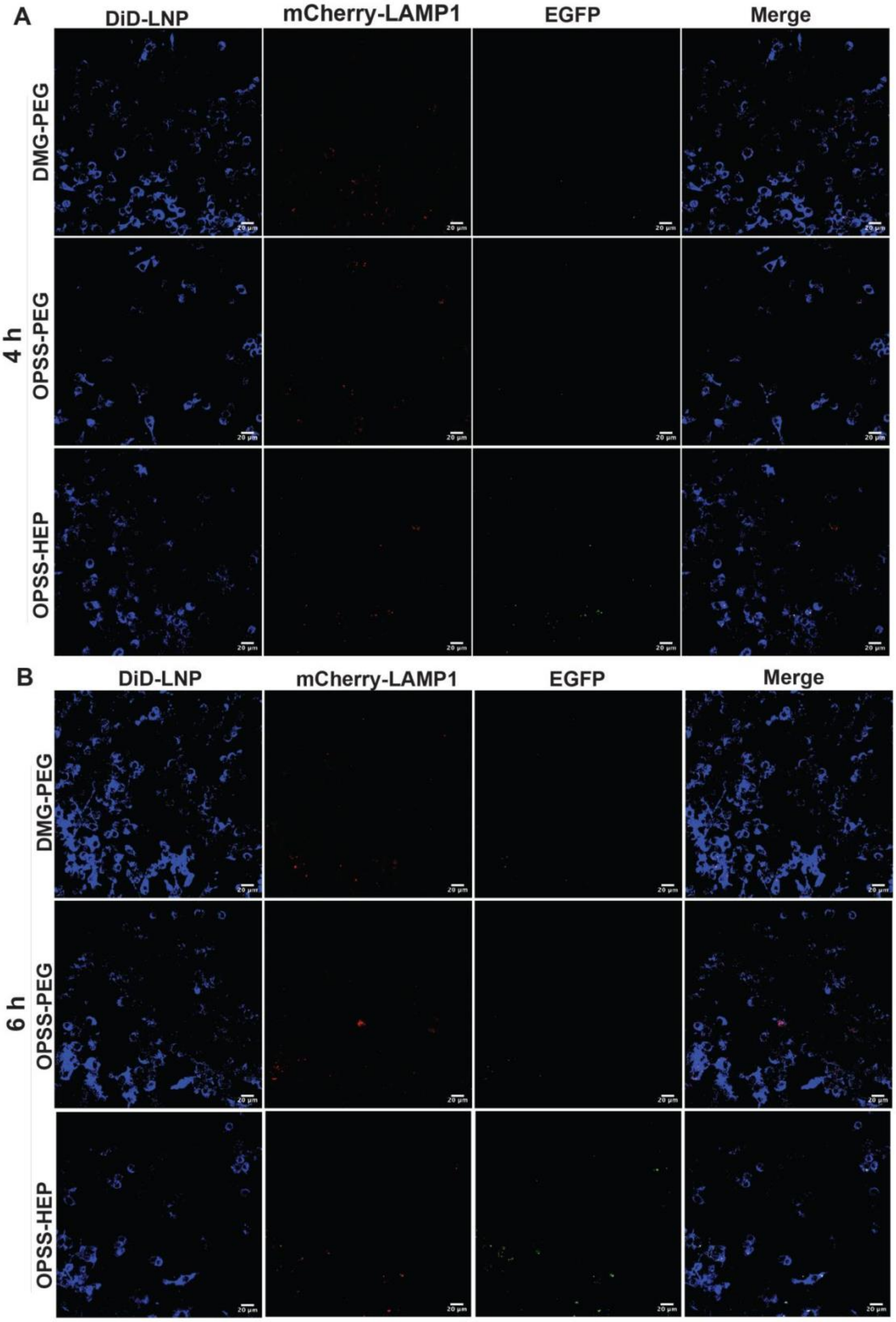

Figure S22: Live-cell imaging and intracellular localization of DiD-labeled EGFP mRNA-LNPs with different surface modifications in RAW 264.7 cells during lysosome transport (4 hours and 6 hours). (A) Representative CLSM images of RAW 264.7 cells expressing

mCherry-LAMP1 (lysosomal marker) at 4 h following treatment with DiD-labeled EGFP mRNA-LNPs coated with DMG-PEG2000, OPSS-PEG, or OPSS-HEP. Scale bars represent 20 μm. **(B)** Representative CLSM images showing mCherry-LAMP1-associated intracellular trafficking at 6 h post-treatment. Scale bars represent 20 μm.

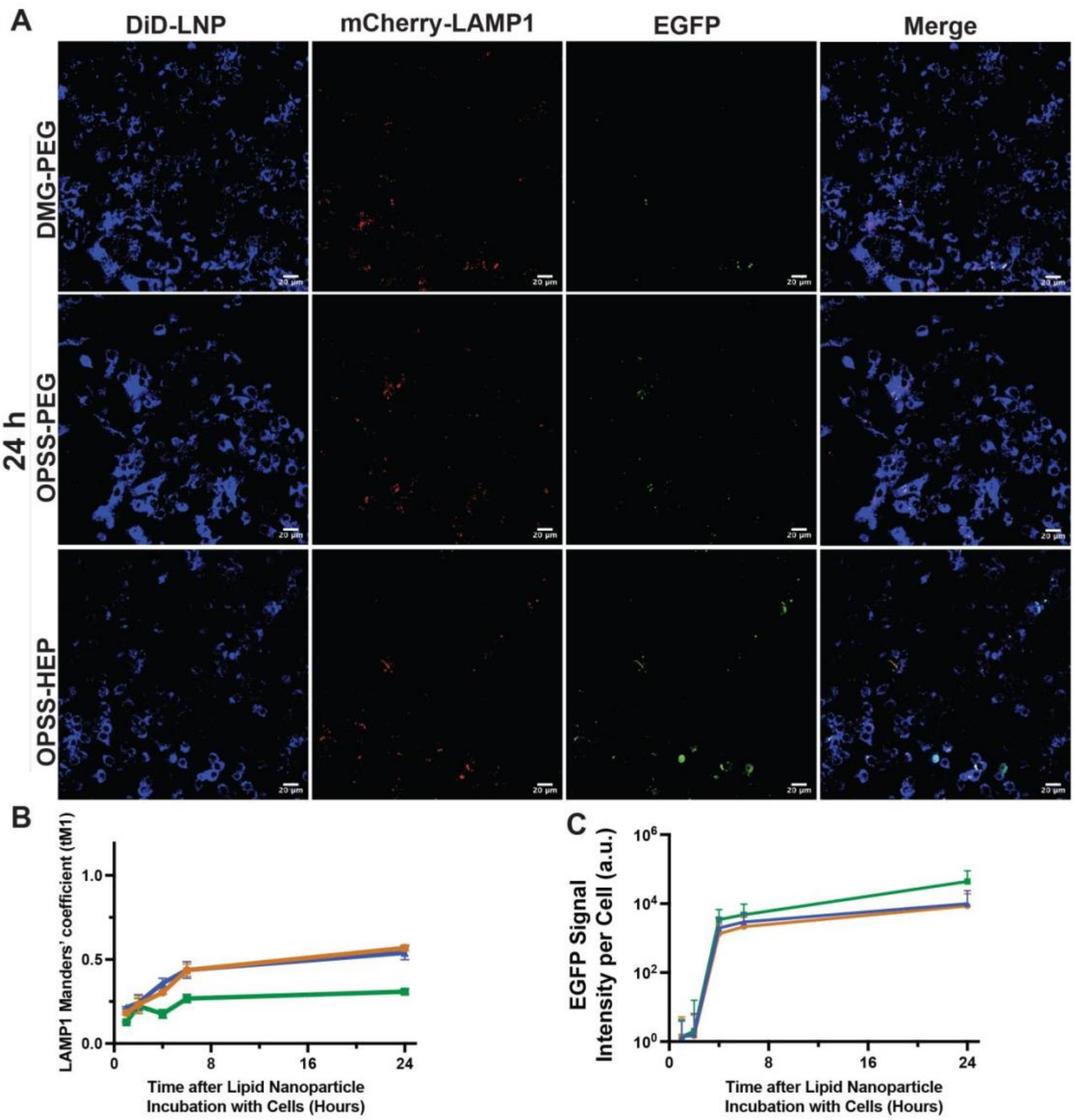

Figure S23: Live-cell imaging and intracellular localization of DiD-labeled EGFP mRNA-LNPs with different surface modifications in RAW 264.7 cells during lysosome transport. **(A)** Representative CLSM images showing mCherry-LAMP1-associated intracellular trafficking at 24 h following treatment with DiD-labeled EGFP mRNA-LNPs coated with DMG-PEG2000, OPSS-PEG, or OPSS-HEP. Scale bars represent 20 µm. **(B)** Quantification of co-localization between DiD-labeled LNPs and mCherry-LAMP1-positive lysosome using Manders' coefficient (tM1) from 1 h to 24 h. Data represent mean ± SD (n=3) **(C)** Time-dependent EGFP expression in RAW 264.7 cells over 24 h following transfection with surface-modified mRNA-LNP formulations. Data represent mean ± SD (n=20). The solid lines are a guide to the eye.

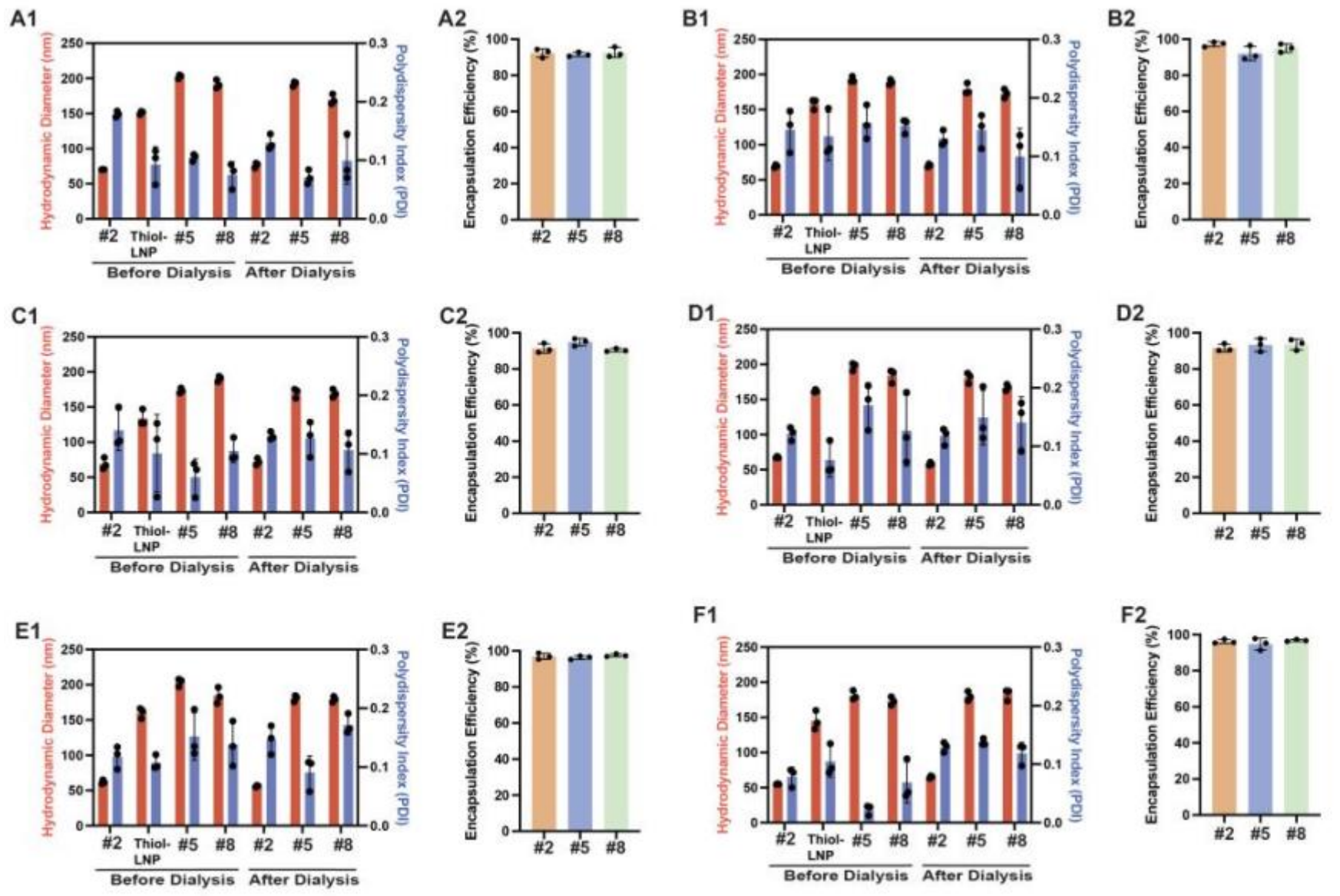

G

Field Flow Fractionation
Multi-Angle Light Scattering
(FFF-MALS)

	Sample ID	Conc (LNPs/mL)
	DMG-PEG #2	4.03×10^{11}
A	OPSS-PEG #5	1.55×10^{11}
	OPSS-HEP #8	1.47×10^{11}
	DMG-PEG #2	5.02×10^{11}
B	OPSS-PEG #5	1.74×10^{11}
	OPSS-HEP #8	1.01×10^{11}
	DMG-PEG #2	5.13×10^{11}
C	OPSS-PEG #5	2.06×10^{11}
	OPSS-HEP #8	2.12×10^{11}
	DMG-PEG #2	5.03×10^{11}
D	OPSS-PEG #5	1.98×10^{11}
	OPSS-HEP #8	2.01×10^{11}
	DMG-PEG #2	4.88×10^{11}
E	OPSS-PEG #5	1.96×10^{11}
	OPSS-HEP #8	1.41×10^{11}
	DMG-PEG #2	4.74×10^{11}
F	OPSS-PEG #5	1.37×10^{11}
	OPSS-HEP #8	1.36×10^{11}

Figure S24: Physicochemical characterization of DMG-PEG2000-, OPSS-PEG-, and OPSS-HEP-modified firefly luciferase mRNA-LNPs used for IVIS studies at an N/P ratio of 8. (A1–F1) HDD and PDI of the indicated mRNA-LNP formulations measured by DLS. **(A2–F2)** %EE of mRNA within the corresponding LNP formulations. Data represent mean ± SD (n=3). **(G)** LNP concentrations of mRNA-LNPs determined by FFF-MALS.

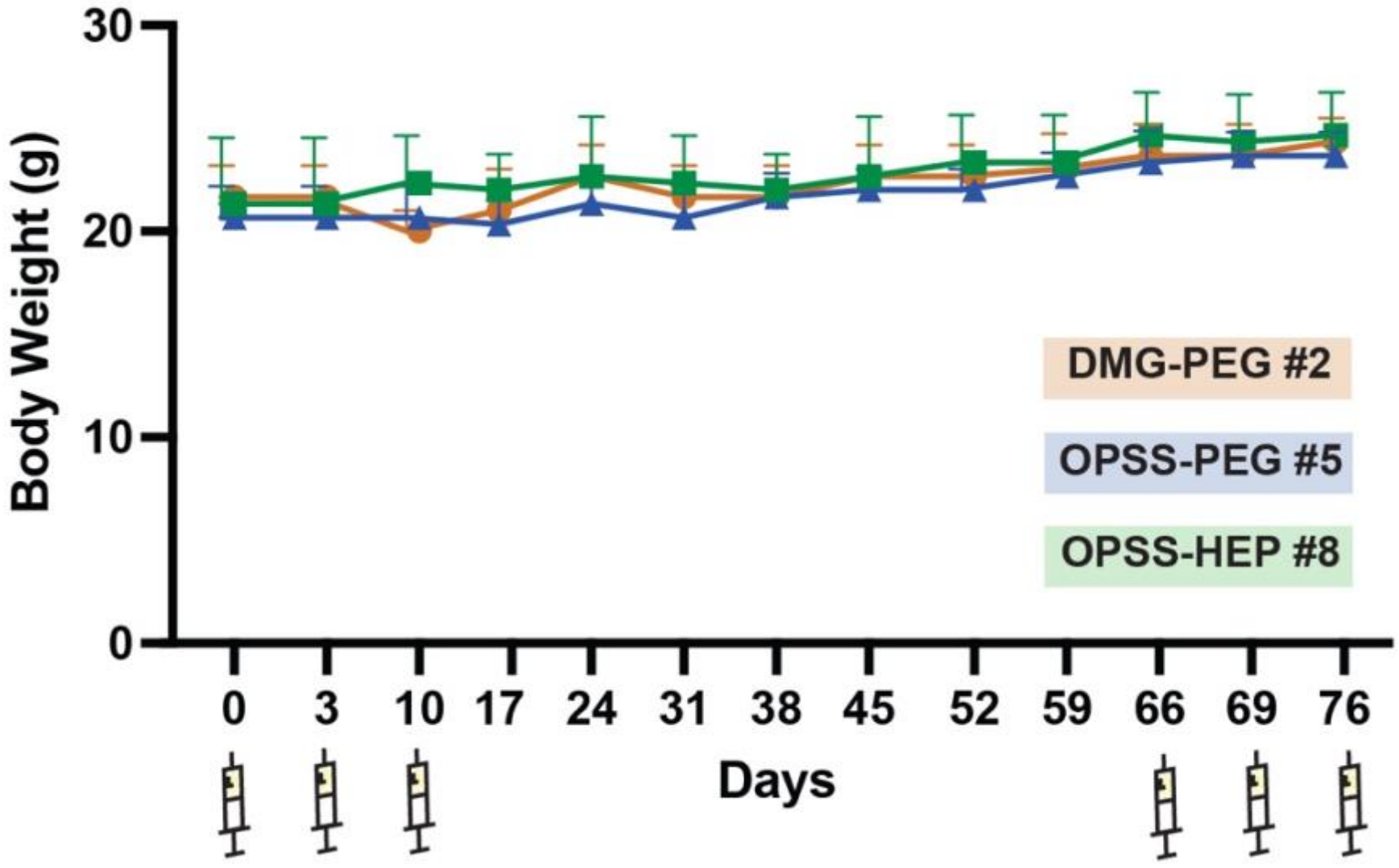

Figure S25: Body weight monitoring of mice following repeated administration of firefly luciferase mRNA-LNPs modified by DMG-PEG2000-, OPSS-PEG-, or OPSS-HEP. C57BL/6 mice were subcutaneously injected with 2 µg of firefly luciferase mRNA-LNPs per mouse on Days 0, 3, 10, 66, 69, and 76, as indicated by syringe icons. Body weights were recorded throughout the 76-day study period to assess potential treatment-related effects. Data represent mean ± SD (n=3), the solid lines are a guide to the eye.

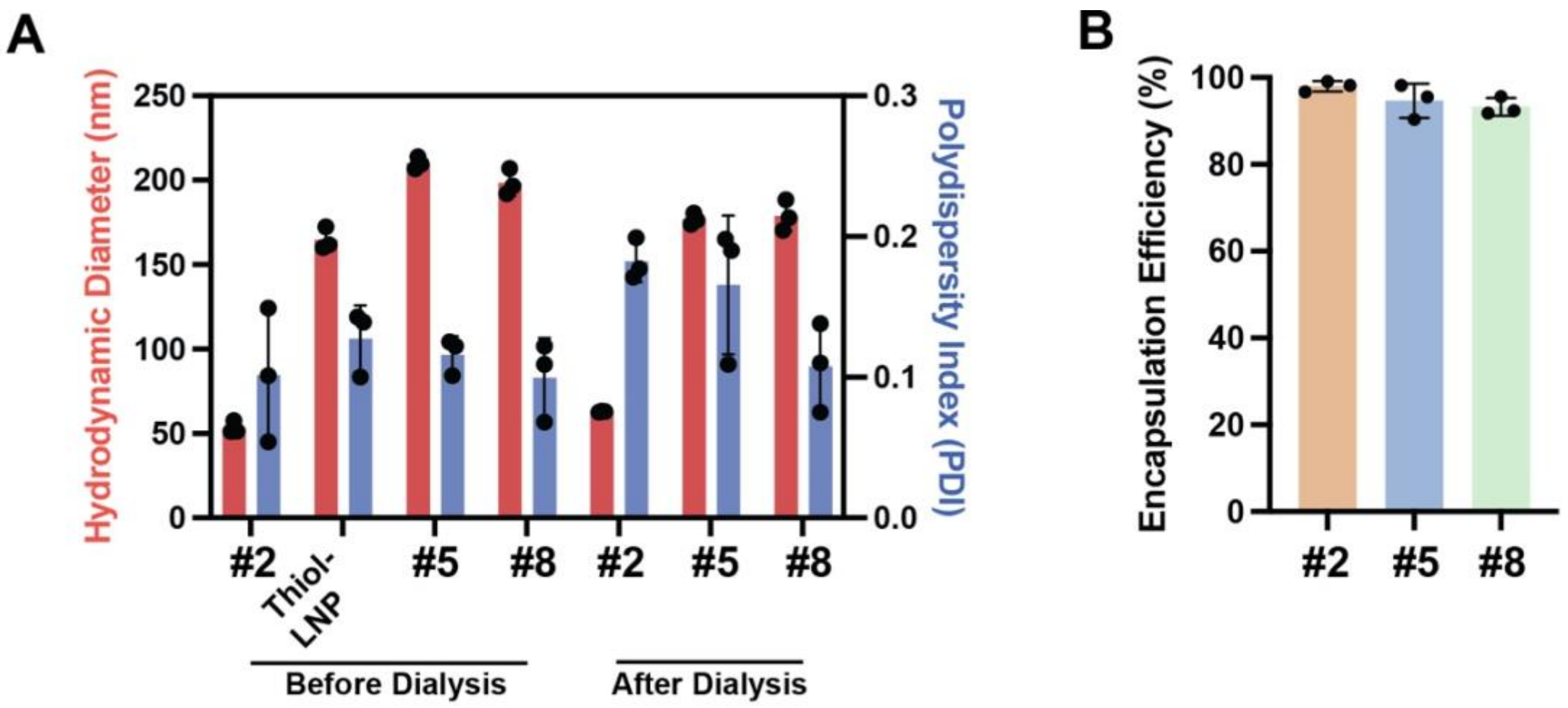

C

Field Flow Fractionation Multi-Angle Light Scattering (FFF-MALS)

Sample ID	Nanoparticle Concentration (LNPs/mL)
DMG-PEG #2	6.12×10^{11}
OPSS-PEG #5	2.13×10^{11}
OPSS-HEP #8	2.08×10^{11}

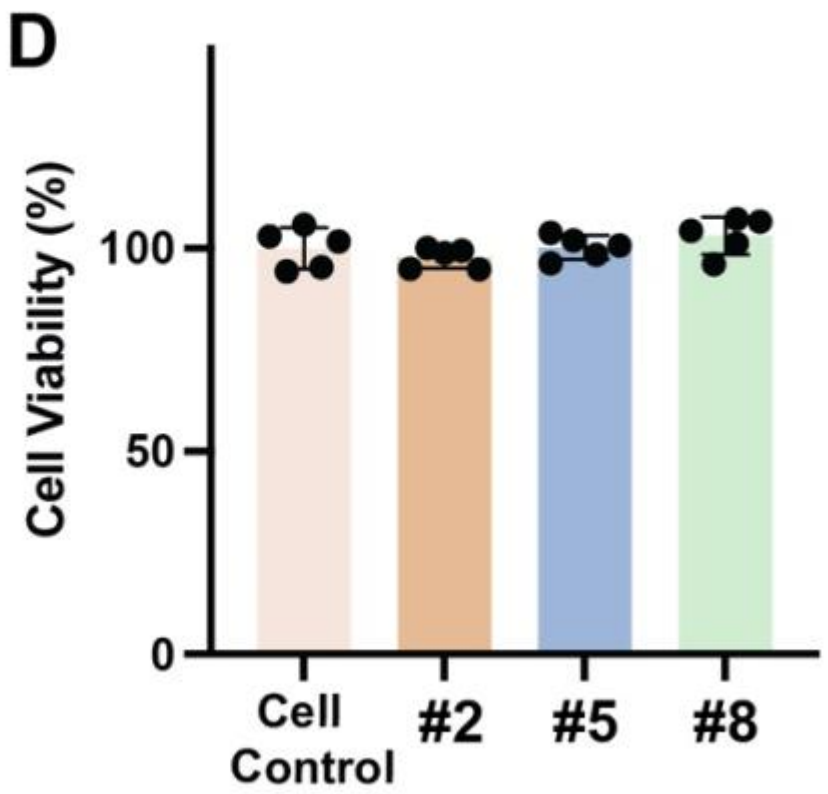

Figure S26: Physicochemical characterization and *in vitro* cytotoxicity assessment of SARS-CoV-2 mRNA-LNPs at an N/P ratio of 8. DMG-PEG2000-, OPSS-PEG-, and OPSS-HEP-modified mRNA-LNPs were evaluated. **(A)** HDD and PDI measured by dynamic light scattering (DLS). **(B)** Encapsulation efficiency (%EE) of mRNA within the LNPs. Data represent mean ± SD (n=3). **(C)** LNP concentration of mRNA-LNPs determined by FFF-MALS. **(D)** Cell viability of RAW 264.7 macrophages following treatment with mRNA-LNPs (0.8 µg mRNA per well) for 24 h, assessed by XTT assay. Data represent mean ± SD (n=5). Statistical analysis was performed using one-way ANOVA; no significant differences were observed among groups (p>0.05).

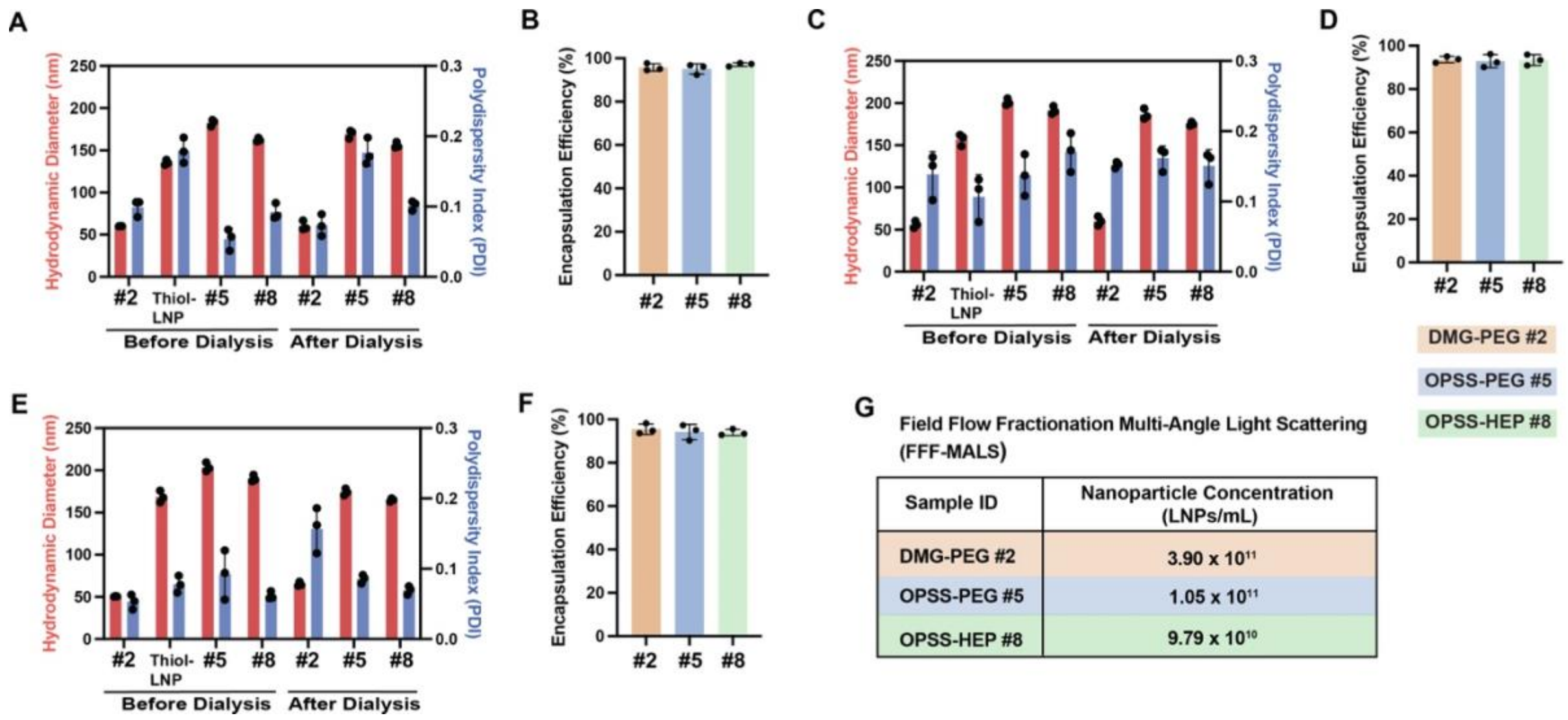

Figure S27: Physicochemical characterization of SARS-CoV-2 spike S-2P mRNA mRNA-LNPs used for three-dose *in vivo* studies at an N/P ratio of 8. DMG-PEG2000 (#2) -, OPSS-PEG (#5) -, and OPSS-HEP (#8) -modified mRNA-LNPs were evaluated. **(A,C,E)** HDD and PDI of the indicated formulations measured by DLS. **(B,D,F)** %EE of mRNA within the corresponding LNP formulations. Data represent ± SD (n=3). **(D)** LNP concentration of mRNA-LNPs determined by FFF-MALS, shown for samples from (A).

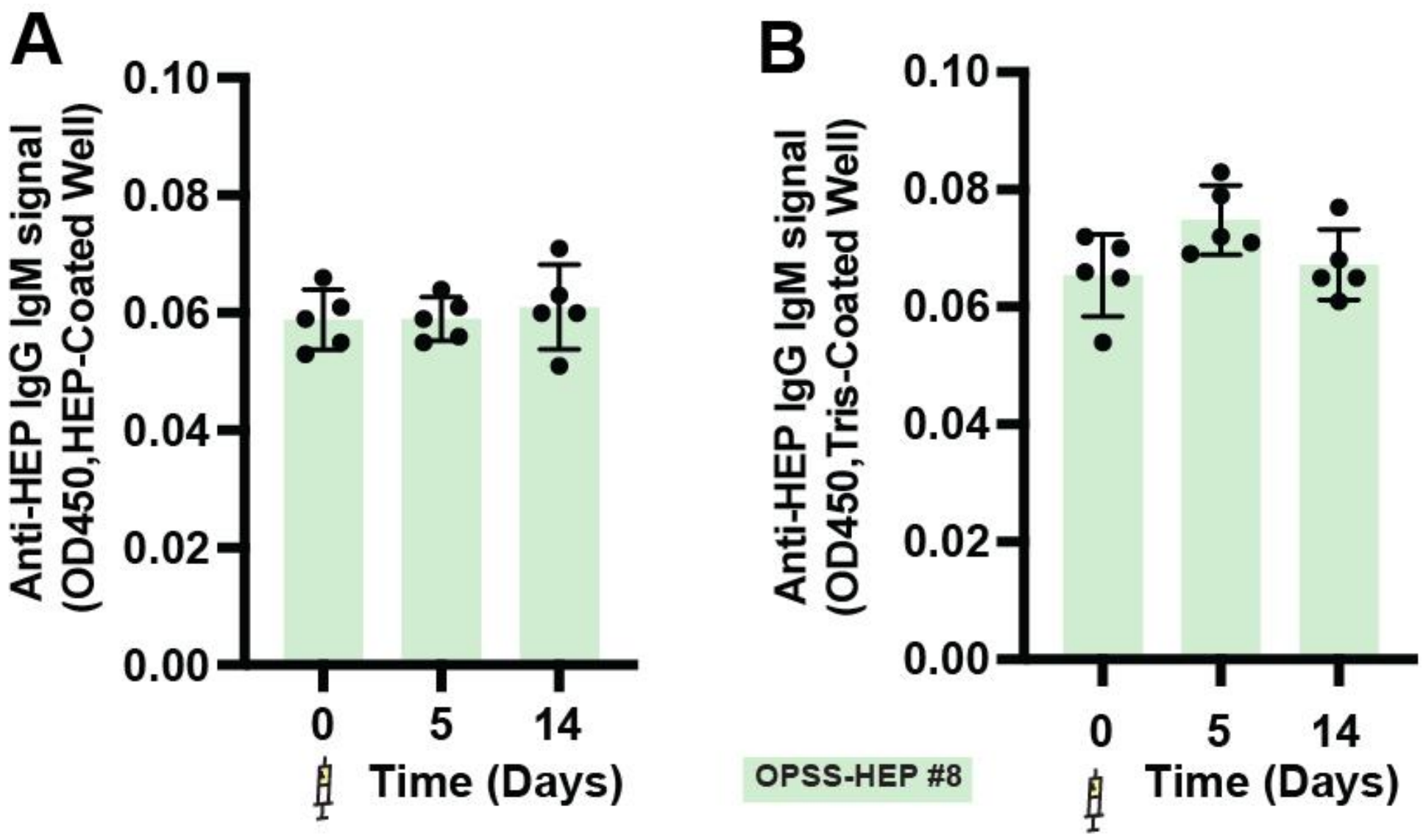

Figure S28: Quantification of potential anti-HEP antibodies in serum from SARS-CoV-2 spike S-2P mRNA OPSS-HEP (#8) mRNA-LNPs-treated mice. The potential presence of anti-HEP IgG and IgM signals in sera (5 µL/well) was measured by ELISA (OD_{450}) using **(A)** HEP-coated 96-well plates or **(B)** Tris-coated plates (no antigen, background control). There were no significant differences in signal intensities, indicating no anti-HEP polysaccharide immune response. Data are presented as mean ± SD (n=5).

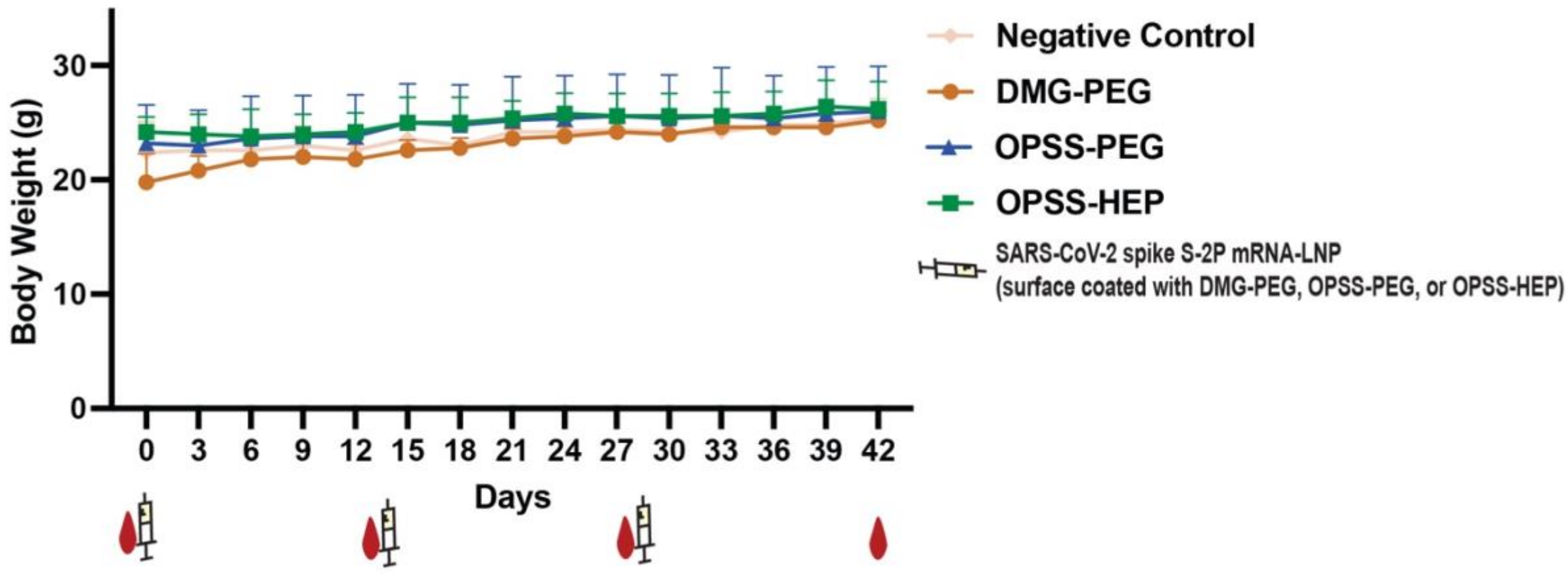

Figure S29: Body weight monitoring of mice following repeated administration of SARS-CoV-2 spike S-2P mRNA-LNPs. C57BL/6 mice were subcutaneously injected with 2 µg of SARS-CoV-2 spike S-2P mRNA-LNPs per mouse formulated with DMG-PEG2000 (#2), OPSS-PEG (#5), or OPSS-HEP (#8) on Days 0, 14, and 28, as indicated by syringe icons. Body weights were recorded throughout the 42-day study period to assess potential treatment-related effects. Blood samples were collected via the submandibular vein prior to each injection and at the study endpoint (Days 0, 14, 28, and 42). Data represent mean ± SD (n=5), the solid lines are a guide to the eye.

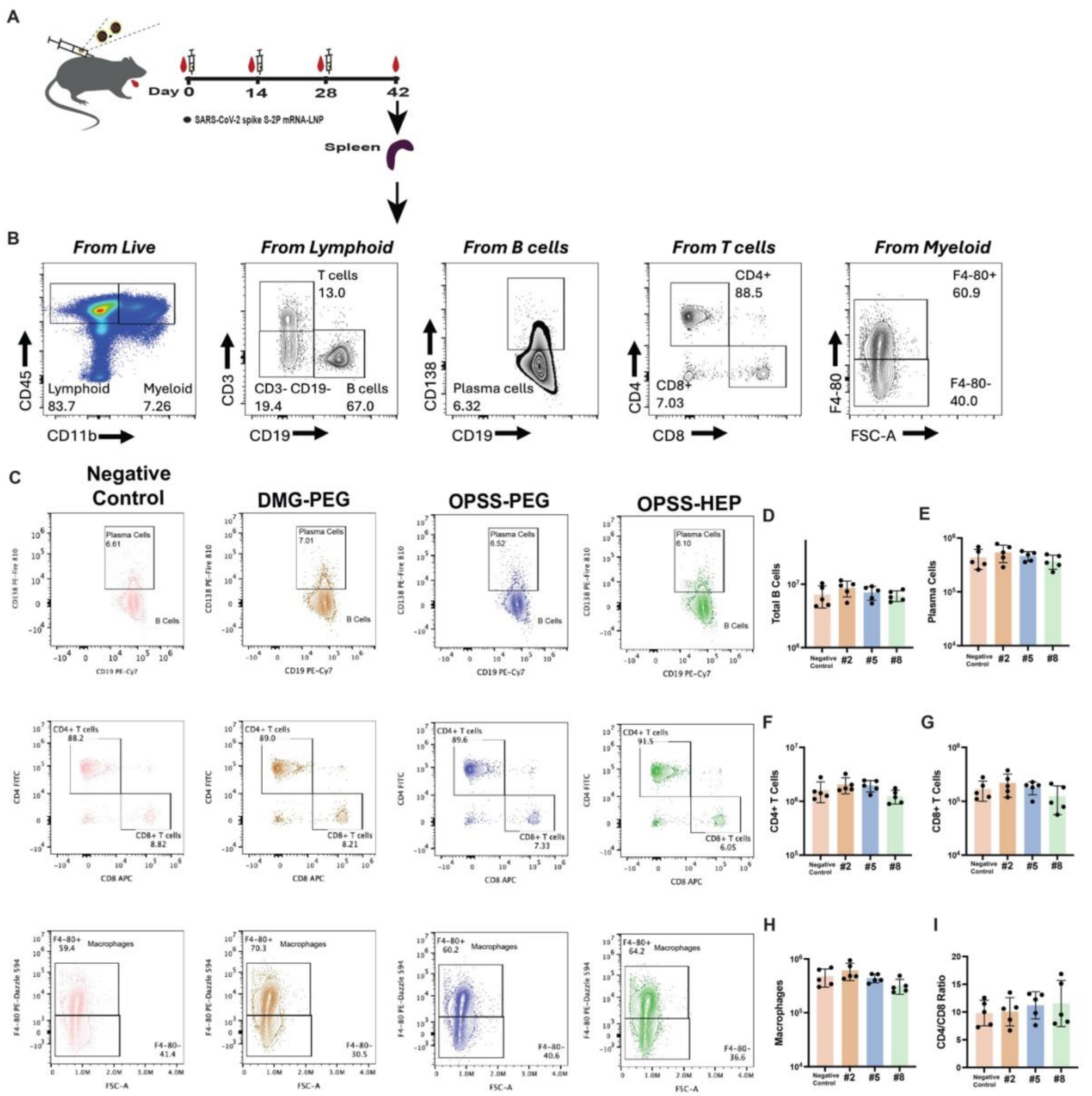

Figure S30: Spleen immune profiling of C57BL/6 mice following repeated subcutaneous administration of SARS-CoV-2 spike S-2P mRNA-LNPs with different surface modifications. (A) Experimental timeline illustrating the immunization schedule and spleen collection timepoint. Mice received three subcutaneous injections of SARS-CoV-2 spike S-2P mRNA-LNPs prior to spleen harvest and analysis. (B) Flow cytometry gating strategy for analysis of immune cells. (C) Flow cytometry analysis of splenic immune cell populations following treatment with DMG-PEG2000 (#2) -, OPSS-PEG (#5) -, or OPSS-HEP (#8) -coated mRNA-LNPs, including plasma cells within the B-cell population, $CD4^+$ T cells, $CD8^+$ T cells, and macrophages (D-I) Quantification of cell numbers for total B cells (D), plasma cells (E), $CD4^+$ T cells (F), $CD8^+$ T cells (G), and macrophages (H). (I) Ratio of $CD4^+$ to $CD8^+$ T cells. Data represent mean ± SD (n=5). Statistical analysis was performed using one-way ANOVA. No statistically significant differences were observed ($p>0.05$).

Table S2: Summary of Histopathological Findings.

	PBS Group	DMG-PEG LNP Group	OPSS-PEG LNP Group	OPSS-HEP LNP Group
SARS-CoV-2 spike (S) protein mRNA<br>mRNA Dosage (㎍/mouse)<br>×3 injections	0	2 ㎍×3	2 ㎍×3	2 ㎍×3
Tissue<br>and observed content	Incidence (a/b)*			
**Liver**				
No abnormalities detected	5/5	5/5	5/5	5/5
**Spleen**				
No abnormalities detected	5/5	5/5	5/5	5/5
**Bone Marrow/ Tibia**				
No abnormalities detected	5/5	5/5	5/5	5/5
**Kidney**				
No abnormalities detected	5/5	5/5	5/5	5/5
**Lung**				
No abnormalities detected	5/5	5/5	5/5	5/5
**Injection site**				
No abnormalities detected	5/5	5/5	5/5	5/5
**Draining Lymph Node (Left Axillary Lymph Node)**				
No abnormalities detected	3/5	4/5	5/5	5/5
Unidentified lymph node	2/3	1/5	0/5	0/5

Summary of histopathological findings in SARS-CoV-2 mRNA-LNP–treated mice. Histopathological changes induced by DMG-PEG2000-, OPSS-PEG-, or OPSS-HEP-modified SARS-CoV-2 mRNA-LNPs were compared with PBS-treated controls across major organs, including liver, spleen, tibial bone marrow, kidney, lung, injection site, and left axillary draining lymph node. Tissues were analyzed by hematoxylin and eosin (H&E) staining, and observed findings were recorded as incidence.

**Incidence is expressed as a/b, where a represents the number of affected samples and b denotes the total number of replicates.*

Mice received three subcutaneous injections of SARS-CoV-2 mRNA-LNPs (2 µg mRNA in 100 µL 1×PBS per dose) or 1xPBS alone, administered at 2-week intervals. Organs were collected 2 weeks after the third injection.
